# Animal behaviour in a human world: A crowdsourcing study on horses that open door and gate mechanisms

**DOI:** 10.1371/journal.pone.0218954

**Published:** 2019-06-26

**Authors:** Konstanze Krueger, Laureen Esch, Richard Byrne

**Affiliations:** 1 Department Zoology and Evolutionary Biology, University of Regensburg, Regensburg, Germany; 2 Department Equine Economics, Section of Agriculture, Economics and Management, Nürtingen-Geislingen University, Nürtingen, Germany; 3 Department of Animal Welfare, Ethology, Animal Hygiene and Animal Husbandry Section of Veterinarian Medicine, Ludwig Maximilian University Munich, München, Germany; 4 Centre for Social Learning & Cognitive Evolution, School of Psychology & Neuroscience, University of St Andrews, St Andrews, Scotland; Institute of Animal Science, CZECH REPUBLIC

## Abstract

Anecdotal reports of horses opening fastened doors and gates are an intriguing way of exploring the possible scope of horses’ problem-solving capacities. The species’ natural environment has no analogues of the mechanisms involved. Scientific studies on the topic are missing, because the rate of occurrence is too low for exploration under controlled conditions. Therefore, we compiled from lay persons case reports of horses opening closed doors and gates. Additionally, we collected video documentations at the internet platform YouTube, taking care to select raw data footage of unedited, clearly described and clearly visible cases of animals with no distinct signs of training or reduced welfare. The data included individuals opening 513 doors or gates on hinges, 49 sliding doors, and 33 barred doors and gateways; mechanisms included 260 cases of horizontal and 155 vertical bars, 43 twist locks, 42 door handles, 34 electric fence handles, 40 carabiners, and 2 locks with keys. Opening was usually for escape, but also for access to food or stable-mates, or out of curiosity or playfulness. While 56 percent of the horses opened a single mechanism at one location, 44 percent opened several types of mechanism (median = 2, min. = 1, max. = 5) at different locations (median = 2, min. = 1, max. = 4). The more complex the mechanism was, the more movements were applied, varying from median 2 for door handles to 10 for carabiners. Mechanisms requiring head- or lip-twisting needed more movements, with significant variation between individuals. 74 horses reported in the questionnaire had options for observing the behaviour in stable mates, 183 did not, which indicates that the latter learned to open doors and gates either individually or from observing humans. Experience favours opening efficiency; subjects which opened several door types applied fewer movements per lock than horses which opened only one door type. We failed to identify a level of complexity of door-fastening mechanism that was beyond the learning capacity of the horse to open. Thus, all devices in frequent use, even carabiners and electric fence handles, are potentially vulnerable to opening by horses, something which needs to be considered in relation to keeping horses safely.

## Introduction

It is unlikely that skills specific to opening door and gate mechanisms have evolved in equids: non-prehensile, hoofed animals which feed on easily accessible, distributed resources **[1]** and roam in open habitats **[2,3]** where there are no analogues of these human-devised fastenings. Any success in dealing with such human-made devices must therefore be based on general learning capacities **[4]**. The present study aimed to evaluate whether the horse has the cognitive capacity to unfasten human-made shutting devices and, if so, whether there are specific preconditions and limits in the animals’ capacity. The question is of practical importance: the ability of horses to open door and gate mechanisms potentially threatens the safety of both horses and humans, and serious accidents have been caused by escaped horses **[5,6]**.

So far, the opening of fastening mechanisms has only been investigated in animals with claws and paws. Reported motivations and aims for opening were various: to gain access to food (chicken **[7])**, comfort areas (chickens **[7]**, blue foxes, *Vulpes lagopus* **[8]**), free movement (blue foxes **[8]**); to free conspecifics (rats **[9]**) by pushing or pulling gates and doors open; or to free themselves to reach food (dogs, cats and chickens **[10]**). Anecdotal reports, however, tell of ungulates, such as horses, donkeys, mules, cattle, and goats, opening mechanically fastened doors and gates. Equids are a good model organism for a detailed study on whether and how a social animal with a strong drive for free movement **[2,11–13]** can deal with closed doors and gates in a human-regulated environment. Horses might acquire the requisite skills individually **[14]** or socially **[15–17]**, i.e. through observing conspecifics **[18–19]** or humans **[20]**. Some studies consider horses’ learning in a social environment to include social learning, mostly through local and stimulus enhancement **[18–20]**; others agree that learning in horses is affected by social circumstances **[21]**, but prefer to reserve the term "social learning" only for cases when observers display an action, which was previously not in their own repertoire, after observing a demonstration **[22]**.

To study infrequent behaviour, analysing a comprehensive collection of anecdotes offers a good starting point. For instance, crowdsourcing studies have analysed the range of flexibility of animal problem-solving abilities **[4]**, play behaviour in dogs and horses **[23]**, and the impact of training in dogs **[24]**. Previous survey studies used several methods: amassing reports written by bird **[25–26]**, primate **[27–31]**, elephant **[32],** wildlife **[33]**, dog **[24]** and horse **[34]** enthusiasts; searching journals for key words such as “unusual” or “novel” **[25–31]**; asking trained personnel and researchers for contemporary reports **[32]**; or searching the internet platform YouTube for video material about rare animal behaviour, as applied in a study on human responses on tail chasing in dogs **[35]** and play behaviour in dogs and horses **[23]**.

Data-mining of this kind runs the risk of collecting biased and occasionally false reports **[25,36,37]**. Responses may be biased by overrepresentation of reports from highly motivated respondents **[38]**, reports about socially desirable items (such as a “clever animal”) or even the respondents’ moods **[37]**. However, the approach potentially provides a large data set of rare observations, which could not possibly be collected by a single research team **[23,26,33,38–40]**. A large sample size increases the credibility of reports **[32]**, whereas a single report, however richly detailed, is scientifically valueless. Sufficiently replicated anecdotes may become reliable data **[36]**, especially if effort is made to exclude unreliable or biased reports **[23,25]**, and when confirmatory data such as pictures or videos are available **[23,29,35,39,40]**. If carefully chosen, YouTube videos can provide high quality, raw footage data without any professional, postproduction editing **[23,39]**.

We employed crowdsourcing **[32]** to search for cases where horses have opened doors and gates: we contacted the owners and caretakers directly or offered to contact us via internet at the web site (https://innovative-behaviour.org). Responders were invited to report about their horses’ “unusual” behaviours, with a general, open-ended questionnaire. The cases of door and gate opening revealed in this instrument provided information for the development of a more specific questionnaire **[36]** on door and gate opening behaviour in horses, which invited a new group of people to report about their animals using the same website. Catch questions were included in both questionnaires to test the reliability of the reports **[34]**. Both questionnaires asked for anecdotal reports, pictures, videos and various other details of any positive cases. In addition, we searched the internet platform YouTube for raw footage videos, without postproduction editing, of horses opening doors and gates **[23,35]**. From both the questionnaire data and the YouTube collection, we deleted any cases that were not clearly described or based on events that had not been clearly visible cases, and cases of animals that had been trained or showed signs of reduced welfare.

In analysing this data, we aimed to evaluate how and why horses opened door and gate mechanisms of different sorts. We asked whether animals learn to open locked doors and gates individually, or socially by observing stable mates **[14,18,19,41]**; whether skills were influenced by the horses’ age, sex or breed **[14,19,42]**; whether opening was influenced by human management conditions **[4,43–45]** or reported motivations and aims, such as gaining access to conspecifics **[9]**, food **[7]**, or free movement **[8]**; and whether there was any limit in terms of cognitive “complexity” of the mechanisms (including the directions and planes in which door and gate mechanisms opened, the specific of movements used or the length of the sequence of movements needed) on horse’s opening abilities. This would provide further insight into the horses’ cognitive capacities and information about appropriate shutting mechanisms to keep horses safely.

## Materials and methods

### Study location and website

We invited owners and caretakers to report on door and gate opening by horses, mules, and donkeys, by means of a website we set up (https://innovative-behaviour.org), contacting potential responders via horse journals, Facebook, various private websites, and at conferences and public talks in Germany, Austria, France, Hungary, Switzerland, the U.K., and the U.S.A. Reports could be submitted in either English, German, or French. In the first phase (from July 2012 to April 2016), a questionnaire asked for reports of “unusual” behaviour with no particular focus (https://innovative-behaviour.org/en/questionary_innovative_behaviour_in_horses). Then (from May 2016 to February 2017), based on preliminary analysis of the reports submitted, we amplified the original questionnaire with more focussed questions on door and gate opening in equids (https://innovative-behaviour.org/en/Questionnaire_horses_that_open_doors_or_gates) (for full questionnaires see S1 and S2 Files; data see S1 and S4 Tables). The data collection was closed in February 2017. In collecting the reports on door and gate opening for the present study we used both questionnaires, ensuring that none of the reports on door and gate opening in the general questionnaire were duplicated in the specific questionnaire.

In addition, we sampled video material from the internet platform YouTube which we found with the key words “open door”, “open gate”, “escape”, “run-away”, “clever”, “horse”, “donkey”, and “mule”. These videos were analysed without downloading them (data and video links see S2 and S4 Tables).

### Ethics statement

We obtained informed consent from all persons who answered the questionnaire. On the website, all responders agreed to the anonymous publication of their data, including pictures and videos for scientific purposes: reasonable requests for access to anonymous agreements can be obtained from the corresponding author. Only the agreed information on the equids and no data of the reporting persons was used for the present study. Some videos were published on YouTube with a Creative Commons CC BY licence (https://support.google.com/youtube/answer/2797468?hl=en&ref_topic=2778546). They are available and can be used without any restriction. Other videos were published with the standard YouTube license. These can be looked at and links can be forwarded without any restriction, which was the default setting for all uploads (see YouTube Terms of Service (https://www.youtube.com/t/terms). Videos at You tube and Facebook are not shown in the study. Links are given for viewing the videos at the providers own web site, which is in line with the copy right terms of the providers. Furthermore, no human data is given in the study. All procedures performed in the study involving human participants were in accordance with the ethical standards of the institutional research committee at Nuertingen-Geislingen University and with the 1964 Helsinki declaration and its later amendments or comparable ethical standards.

### Data and data selection

In total, we found 419 cases in which equids opened doors or gates. The cases ran through a selection process (S1 Fig). We excluded 7 of the 342 cases which came from our website. Four cases were excluded because they reported about trained behaviour (i.e. people confirmed that they trained the behaviour or reinforced the behaviour verbally or with food), and 3 because they were possibly the result of reduced welfare (i.e. opening a mechanism simply by chance while showing stereotypic, repetitive behaviour). None of the other reports provided any indication that horses were underfed, injured or unsound. In addition, we excluded mule (N = 1) and donkey (N = 1) survey cases from analysis because of their low sample size. The remaining 333 cases from the website included 45 reports accompanied by pictures of the door and gate mechanisms, and 9 reports with videos showing the techniques used to open doors and gates.

Material from YouTube added 68 videos, giving 77 videos in total. Videos were similarly run through the selection process (S1 Fig): only 69 were finally analysed. Four did not show the behaviour clearly, and the sample size of mule (N = 1) and donkey (N = 3) videos was considered too small to allow for analysis.

The remaining cases were rated by three independent persons, one professor and two bachelors in equine science, as to whether further reports and videos should be excluded from the study based on reduced welfare, and they agreed in all but one case (inter observer agreement: κ = 0.98). The case in question was therefore excluded as well (for remaining raw data see S1–S4 Tables).

### Questionnaire on opening doors and gates

We used a quantitative–qualitative mixed questionnaire approach **[46]**. Catch questions were included in both questionnaires to test the reliability of the reports **[34]**. The first, general questionnaire invited people to report about their horses “unusual” behaviours, including an open question for hypothesis testing (https://innovative-behaviour.org/en/questionary_innovative_behaviour_in_horses); see complete questionnaire at S1 File). The reported cases of door and gate opening provided information for the development of the additional questionnaire **[36]** which focused specifically on door and gate opening behaviour in equids (https://innovative-behaviour.org/en/Questionnaire_horses_that_open_doors_or_gates); see complete questionnaire at S2 File). We went into more details in raising data for evaluating hypotheses on whether door and gate opening was observed in stable mates, on the reported motivation or aim to open a door or gate and on the complexity of the locks horses were capable to master.

The specific questionnaire on door and gate opening asked three open questions, two semi-closed questions, and 25 closed questions in a semi-random order to prevent order biases in the responses **[46]**. Two questions asked whether other horses in the same stable showed the behaviour before or after the behaviour was observed in the focus animal. Six questions related to whether the behaviour was demonstrated or reinforced by humans. Four questions related to the management of the animals: a) the type of housing—were animals kept in single or group housing; b) whether the animals had daily access to pasture or on a limited number of days per week; c) whether they were in permanent or temporary contact with other equids; and d) whether they received limited or unlimited roughage. Five questions concerned what the individual animals did after opening doors and gates: a) stayed in the stable, b) visited other equids, c) moved around freely, d) broke into other places, such as feed storage rooms or human houses, or e) freed other equids. Three closed questions requested information on the number of mechanisms the animals mastered, the frequency of the reported behaviour, and whether the process of developing the behaviour was observed. With a semi-open question, we asked for the upload of pictures and videos of the mechanisms and/or the door and gate opening behaviour. The open questions asked for a description, drawings or pictures of the mechanisms the animals opened at the doors or gates and for further suggestions or questions regarding the project. Three questions asked the breed, sex and age of the particular equid. Finally, we asked for the email address of the reporting person, for permission to use their email address for further enquiries, and whether they agreed with the use of their reports for scientific purposes and publications.

### Animals

The 402 horses reported were of various breeds which we summarised in breed types, as used in genetic studies **[47,48]**: Thoroughbred horses (N = 4), Draught horses (N = 22), Arabian horses (N = 41), Ponies (N = 48), and Warmblood horses (N = 240). In 46 cases the breed was not reported or was not obviously visible in the videos. Subjects were 111 females, 230 castrated males, 19 uncastrated males and 46 equids for which we could not discern the sex. The mean age at which horses were reported to have started opening doors and gates was 10.14 years (SD = 6.27).

### Doors, gates and mechanisms

In principle, individual animals might open doors or gates at only one location or at several (e.g. their own or other animals’ box doors, feed room doors, house doors, or pasture and paddock gates), and the fastening mechanisms might be of the same or of different types.

**Fig 1:**
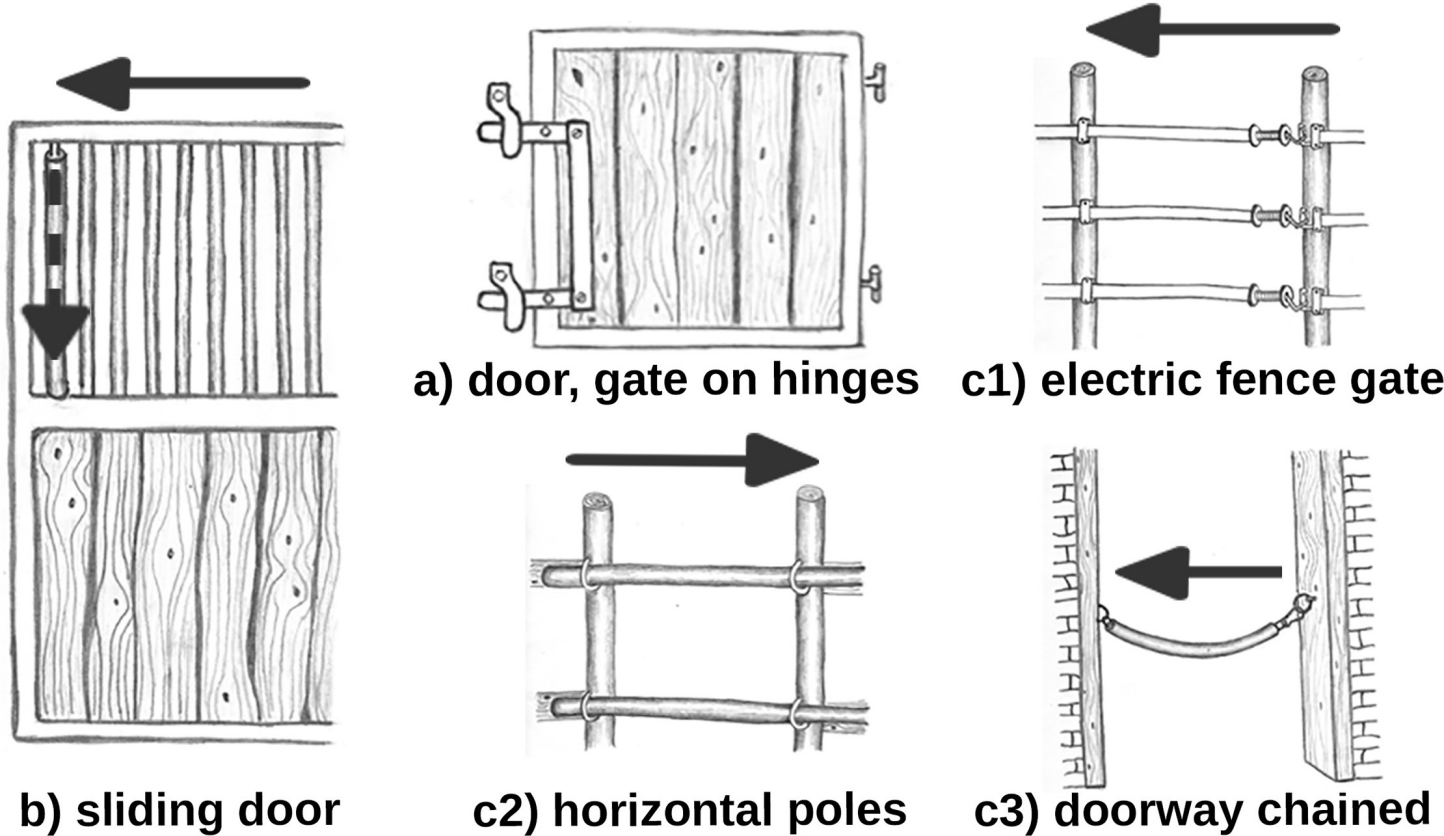
Door and gate types opened by horses. The arrows indicate orthogonal door and gate opening directions. Doors and gates on hinges were opened in the same or opposite direction to the animals’ movement direction. The doors and gates c1)–c3) are subcategories of the category c) barred door or gateways. At b), horses had to grasp and pull down a pipe with their mouth at the dotted arrow and then move the door into the direction of the continuous arrow.

We categorized doors and gates into 3 types (Fig 1, see video links in S2 Table):

- door or gate suspended on hinges and pushed open
- sliding door, suspended on wheels above the horses’ heads, and rolled open
- barred door or gateways, subcategorized in:
  1. electric fence gate, gate supplied with electric power, consisting of electric fencing and power-free handles which have to be unhooked from the fence to create a gateway
  2. gate with horizontal wooden poles, wooden fencing with wooden poles that slide out of an impression or frame to create a gateway
  3. doorway chained so that chain blocks an opening, usually by attachment to the other side of a fence or stall door with a carabiner

**Fig 2:**
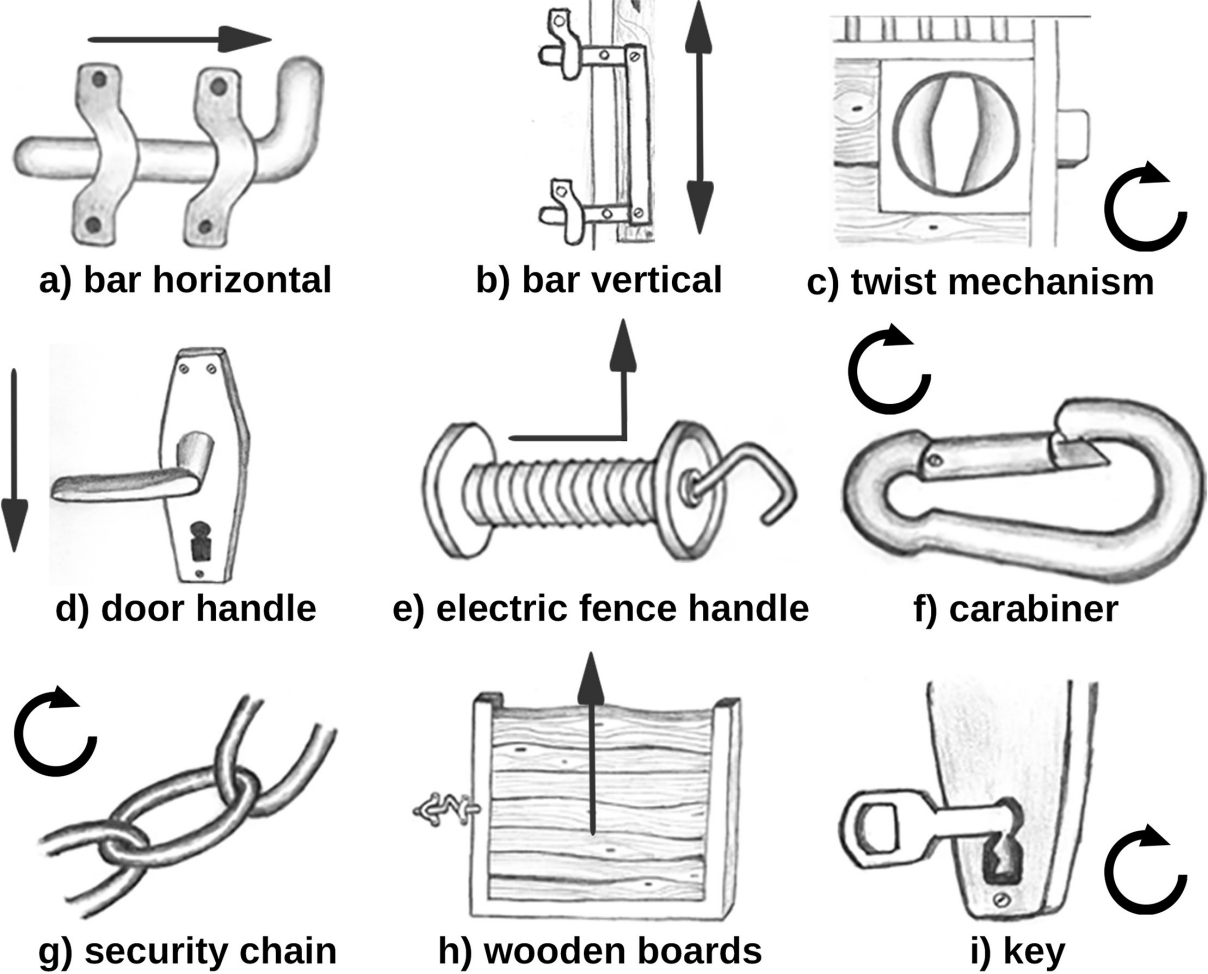
Door and gate mechanism types opened by horses. Arrows indicate the direction of the animals’ head movements.

Mechanism types were categorized as follows (Fig 2, see video links in S2 Table):

- horizontal bar, which had to be moved backwards and forwards, including wooden gate poles
- vertical bar, which had to be moved up or down, including bars which secured sliding doors
- twist mechanisms controlling a bolt, where a disc had to be turned to the left or right to close or open the bolt
- door handle
- electric fence handle
- carabiner, which connects chains or secures other devices. Carabiners could have clip or screw mechanisms
- security chain, which secures gates or other mechanism types
- wooden boards that were pulled out of a door frame
- locks with keys, including padlock with key

### Door, gate and mechanism complexity (video material only, N = 69)

We evaluated the “complexity” of the doors, gates and mechanisms in three ways, with respect to: the direction of overall movement; the plane of head movement; and the number of head movements.

**Fig 3:**
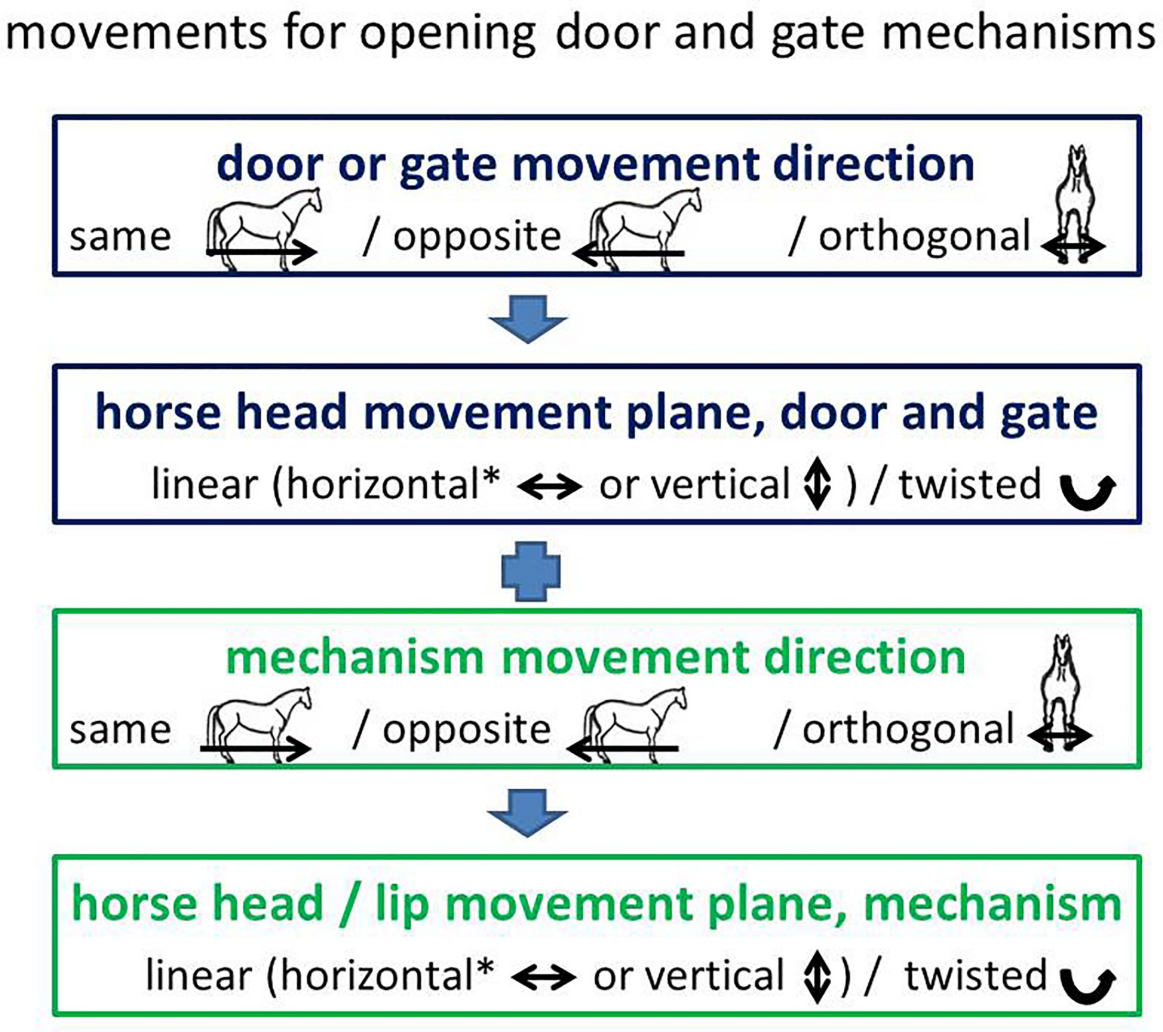
The horses’ movements for opening door and gate mechanisms. *The horizontal movement plane was further distinguished into left-right and backwards–forwards.

We categorized the direction in which the doors, gates and mechanisms opened in relation to the animals’ movement direction after the opening (see Fig 3): a) in the same direction, b) in the opposite direction, or c) orthogonal to the movement direction.

**Fig 4:**
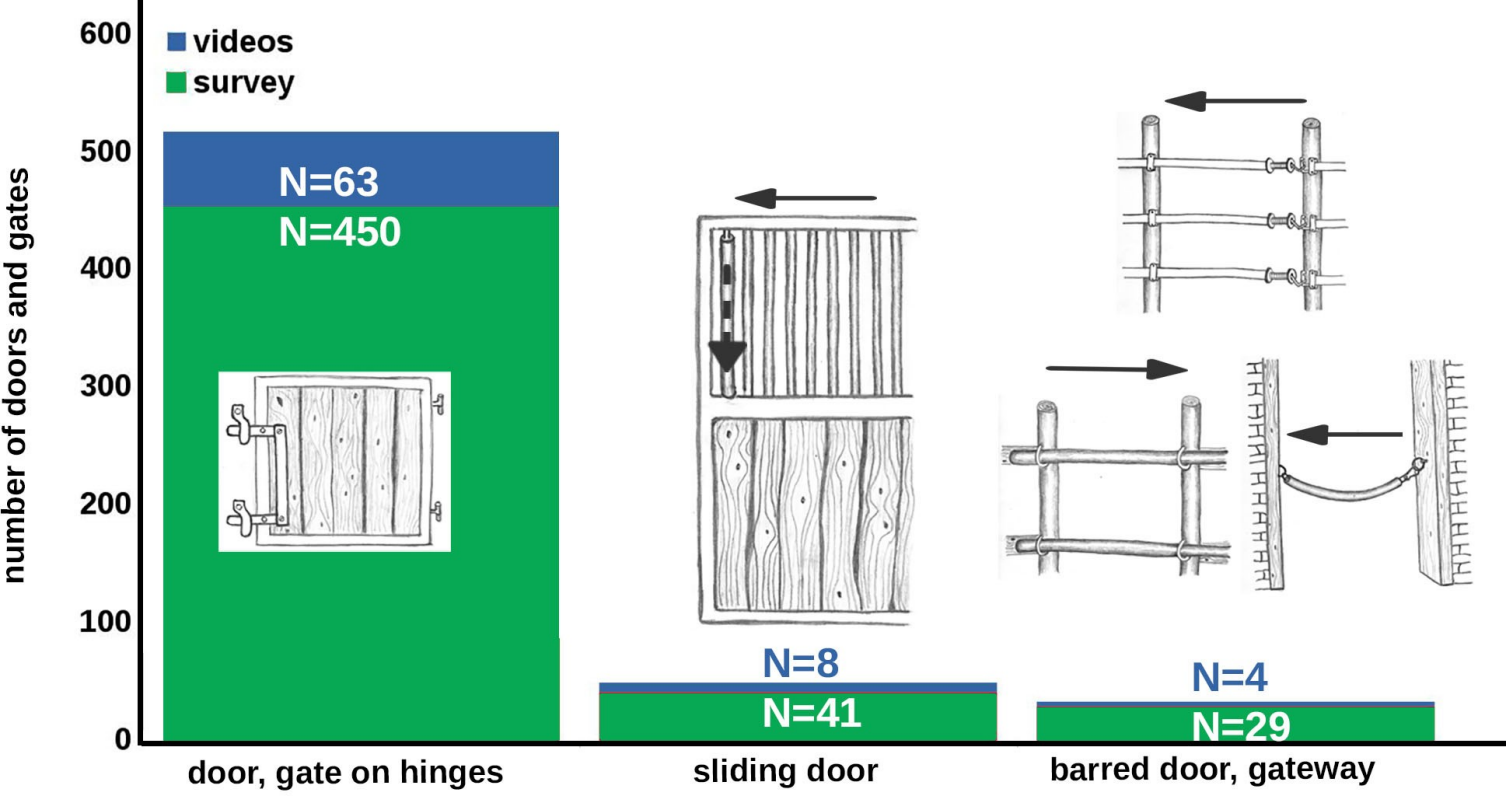
Frequency of door and gate types opened by horses.

**Fig 5:**
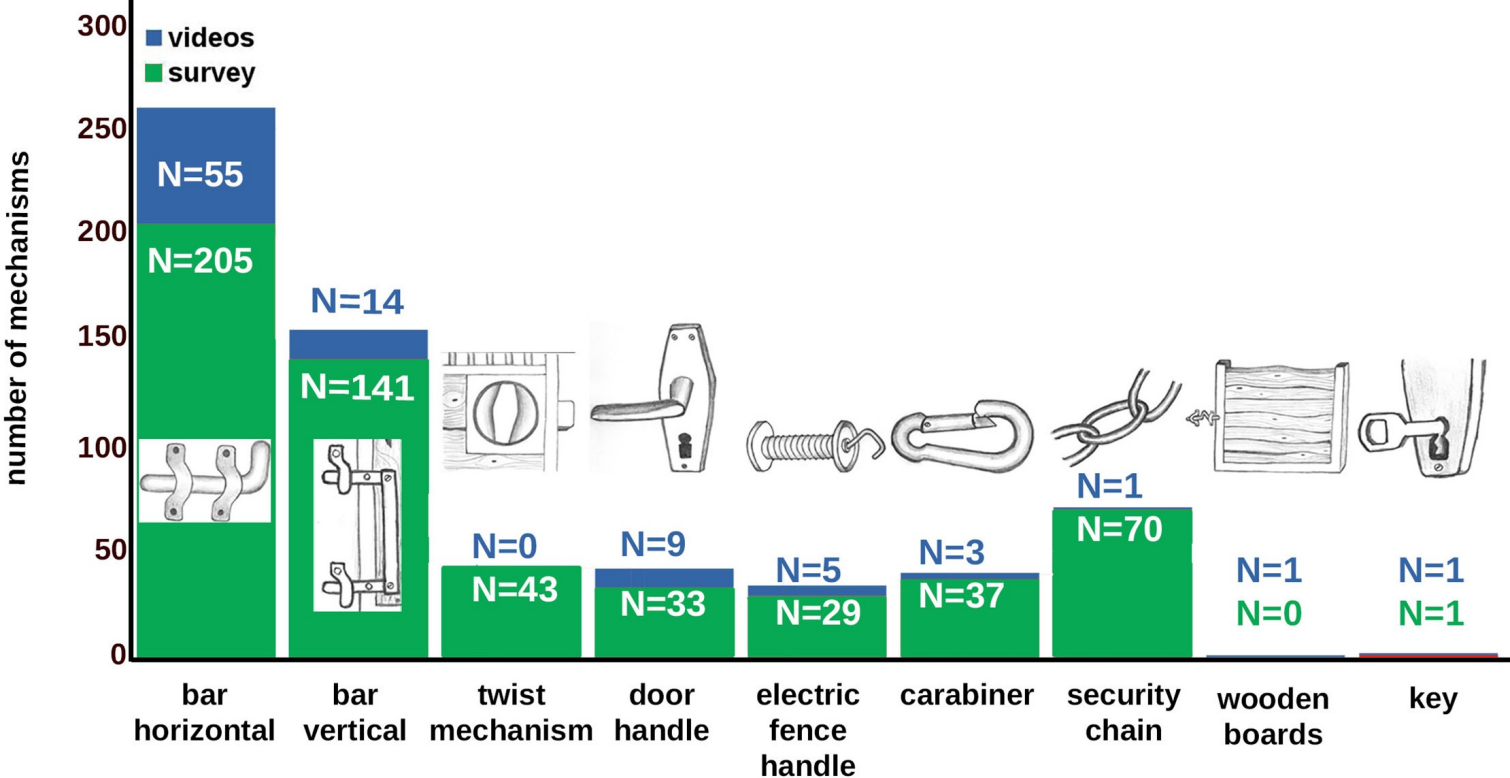
Frequency of door and gate mechanism types opened by horses.

From preliminary data analysis it was evident that all the horses opened the locked doors and gates with their mouths. We categorized the movement of the animal’s head or lips (for simplicity, “head movements”) according to linear movement planes and whether twisting was involved (see Figs 3–5). Five categories were distinguished: a) left and right (horizontal), b) back and forth (horizontal), c) up and down (vertical), d) twisting the head once or several times for at least 45° to the vertical, and/or e) twisting their lips (if visible) once or several times for at least 30° to the main head-axis. Any fine motor skills which the horses needed for manipulating the mechanisms with their tongues were not visible in the videos of the present study and therefore were not considered.

**Fig 6:**
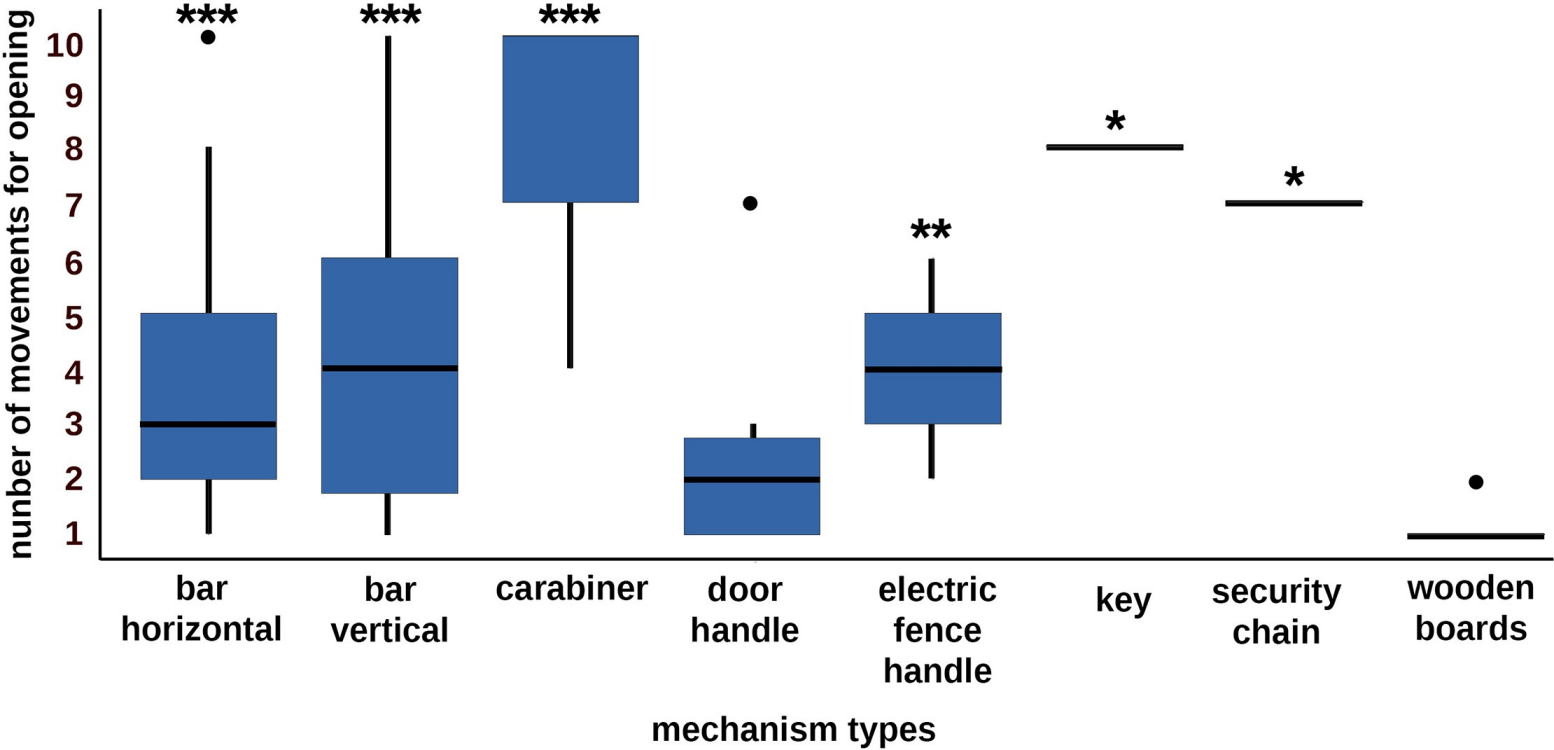
Numbers of movements horses used for opening mechanism types at the videos. Opening carabiners (median = 10, min. = 4, max. = 10, GLM: N = 89, Std.E. = 2.27, z = 3.91, p < 0.001), locks with keys (median = 8, min = 8, max = 8, GLM: N = 89, Std.E. = 2.85, z = 2.41, p = 0.02), security chains (median = 7, min. = 7, max. = 7, GLM: N = 89, Std.E. = 2.67, z = 2.12, p = 0.03), and bolts which had to be pulled vertically (median = 4, min. = 1, max. = 10, GLM: N = 89, Std.E. = 0.63, z = 4.43, p < 0.001) and electric fence handles (median = 3.5, min. = 2, max. = 6, GLM: N = 89, Std.E. = 0.97, z = 2.96, p = 0.003) needed more movements than opening handles (median = 2, min. = 1, max. = 7, GLM: N = 89, Std.E. = 0.62, z = 1.76, p = 0.08) and locks with bolts which had to be moved horizontally (median = 3, min. = 1, max. = 10, GLM: N = 89, Std.E. = 0.46, z = 6.19, p < 0.001). Twist mechanisms (c) were not documented. The boxplots visualize the quartiles of the data per lock type. The box comprises 50% and the lower and upper whisker 25% of the variability each. The dots visualize outliers. *** = p < 0.001, ** = p < 0.01, * = p ≤ 0.05.

For each opening of the doors, gates and mechanisms we counted:

- The total number of head movements the individuals had to apply to open the different doors, gates, and mechanism types (Figs 4 and 6). We counted all movements when the horse was in contact with doors, gates or mechanisms until final opening; each movement was considered finished when motion ceased or changed direction.
- The frequency in which horses displayed series of head movements. We considered responses to form a series when the individual was in contact with a door, gate or mechanism and displayed one or more continuous movements until it turned away from the door, gate or mechanism to at least 30° and /or interrupted its movements for at least 3 seconds. A series of actions was counted as part of the same door or gate opening instance, if the horse continued with further manipulations of the door, gate or mechanism within 1 minute of the preceding sequence. Movements within a series were mostly, but not always, ordered in sequence—some animals displayed opening movements which were not necessarily needed to open a door, gate or mechanism.
- The efficiency of the movements. We measured the number of head movements performed to open the doors, gates and mechanisms, and compared it to the minimum number of movements which would be needed by a person (efficiency = minimum number of movements / actual number of movements).

Doors, gates and mechanisms that opened in one direction, in one plane and with not more than two movements were termed “simple”. Doors, gates and mechanisms which required movement in several directions of the same movement plane, or on several movement planes, or twisting head or lips were considered more “complex”

### Data analysis

For statistical analysis and the depiction of the data we used the package R commander of the R-Project statistical environment, Libre Office 4.3.3.2 and Photo shop CC2017. Most of the data were not normally distributed (Kolmogorov-Smirnov test). Therefore, we applied tests suitable for non-parametric data throughout. In cases where clear cut descriptive data were offered, we did not apply inferential statistics. For more complex data and research questions, we applied Generalized Linear Models (GLM) and Generalized Linear Mixed Models (GLMM).

We applied GLMs when only fixed effects were calculated. GLMs set at ‘family Poisson’ were calculated for the dependent variables a) door and gate types, b) the mechanism types, and c) the mechanism positions; fixed factors were age of the horses and whether other horses showed the same behaviour. A separate GLM was set at ‘family Binomial’ for the now dependent variable whether other horses showed the same behaviour; fixed factors were door and gate types, the mechanism types. Further GLMs set at ‘family Binomial’ were analysed for the depended variables horses a) stayed in the stable, b) visited other equids, c) moved around freely, d) broke into other places and e) freed other equids; fixed factors were the management of the animals, i.e. single or group housing, daily access to pasture or on a limited number of days per week, permanent or temporary contact with other equids, and limited or unlimited roughage.

We applied GLMMs when several mechanisms opened by particular horses were part of the analysis and considered the ID of the horse as random factor. Dependent variables of the GLMMS were a) the total number of head movements, b) the frequency in which horses displayed series of head movements, and c) the efficiency of the movements. Fixed factors were the door and gate types, the mechanism types, the number of head and mouth movements, and the number of head movement directions (for definition see above). The GLMMs were set at family ‘Poisson’.

The model with the best fit (with the lowest AIC index) was chosen after stepwise removal of factors. In cases reduced models were used the AIC of the particular model is given in the results and complete and reduced models are provided at S3 File. All tests were two-tailed and the significance level was set at 0.05.

## Results

### Opportunity to observe door and gate opening (survey N = 333)

In 257 of the 335 cases, people reported on whether the animals had the option to observe the behaviour in stable mates and whether the behaviour was shown by further stable mates after the focus animal displayed it. Seventy-four animals had the option to observe the behaviour in a stable mate. In 61 reported cases the behaviour was later shown by others in the same stable. Whether animals were reported to have the option for observing the door and gate lock manipulations in stable mates did not have any effect on how many different doors and gates, how many different locks, and how many locks in different locations were opened (GLM: N = 333, all p > 0.05). Subjects which had the option to observe the manipulation in conspecifics did not open any particular door, gate or lock types preferentially (GLM: N = 333, all p > 0.05); only twist locks were opened more often by animals which had no option to observe the opening (GLM: N = 333, SE = 0.51, t = -2.08, p = 0.04). Five animals (7%) with the option to observe conspecifics at the same lock type and 29 (16%) with no option to observe the manipulation opened twist locks.

### Type of door and gate opening horses (survey N = 333)

We found no effect of the animals’ age (mean = 10.14, SD = 6.27) on the number of doors and gates or number of mechanism types that were opened, or on the number of locations in which the mechanisms were opened (GLM: N = 333, all p > 0.05). Many of the gate and door openers were castrated male animals (N = 212) and many of them were warmblood horses (N = 207), but as the sex and breed distribution may be biased by preferences of the persons who responded to our query, we do not consider the breed and the sex for further comparisons.

### Management conditions of horses opening doors and gates (survey N = 333)

Management conditions were approximately equally distributed for the reported animals. People kept 52% of the animals in single housing and 48% in group housing; 56% of the horses had daily access to pasture, 44% access to pasture on a limited number of days per week; 57% were in unrestricted contact with other horses, 43% in restricted contact; 47% of the horses received roughage ad libitum, 53% received restricted roughage.

### Motivation and aims for doors and gate opening (survey N = 333)

After opening the doors and gates of a box, enclosure or pasture, 87% of the animals walked out, 62% ran around in the area surrounding their stable, 22% went into other horse boxes or stables, 15% freed other horses, and 22% broke into other places such as feed storage rooms or human houses. Horses tended to stayed in their boxes or pastures after opening the door or gates when other horses in the same stable showed the same behaviour before (GLM, AIC = 303.36: N = 333, SE = 0.24, z = -1.82, p = 0.07) and were more likely to have daily access to pasture than those that exited the boxes or pastures (GLM, AIC = 303.36: N = 333, SE = 0.28, z = -2.515, p = 0.01). Furthermore, those that went into feed storage rooms were more likely to have daily access to pasture (GLM, AIC = 312.21: N = 333, SE = 0.27, z = 2.41, p = 0.02) and were older (mean: 11.5 years, SD = 6.9) than horses that did not open feed storage rooms (mean: 9.6 years, SD = 5.8). All other management restrictions were unrelated to actions after opening doors and gates (GLM: N = 333, all p > 0.05).

### Types of door and gate mechanisms (survey N = 333, videos N = 69)

The case reports describe the opening of 520 door or gate types, and the videos show the opening of 75 doors and gates (for frequencies see Fig 4); the case reports describe the opening of 559 mechanisms, and the videos 89 mechanisms (for frequencies see Fig 5). Some reports did not provide details of the mechanism types.

Most reports described the opening of one door or gate per animal (median, min. = 1, max. = 4). In the survey, the animals typically opened one door or gate type (median, min. = 1, max. = 2) and two mechanism types (median, min. = 1, max. = 5), at two locations (median, min. = 1, max. = 4). In the videos, the animals opened a median of one door or gate type (min. = 1, max. = 2), one mechanism type (min. = 1, max. = 3), at one location (min. = 1, max. = 6).

### Complexity of door and gate mechanisms (videos N = 69)

#### Horses’ movements for opening doors, gates and mechanisms

Most of the analysed doors and gates (N = 75) opened in the direction of travel of the horse manipulating the mechanism (52 away from the horse = *same* direction, 11 towards the horse = *opposite* direction, see Fig 3). Other types of gate required movements *orthogonal* to the horses’ direction of travel (8 sliding doors, 4 barred doorways or gateways). Moving the head in a linear movement plane was sufficient to open most of the doors and gates but twisting of the head was needed for 6 doors or gates on hinges, 3 sliding doors and 1 barred door- or gateway.

All mechanisms (N = 89) had to be opened orthogonally to the travel directions of the animals, except one bar which had to be pulled in the direction opposite to that of travel. Most mechanisms were opened with a linear head movement: 19 bars were moved horizontally (14 left and right, 5 backwards and forwards); 20 bars were moved vertically (upwards or downwards); 32 bars were moved horizontally and vertically, 9 door handles were pulled vertically downwards; 5 electric fence handles were moved horizontally left or right towards the supporting pole of the electric fence and then upwards out of wire loops at the pole; 8 wooden boards were pulled vertically upwards out of a door frame. However, some mechanisms had to be opened with twisting movements: 4 sideways and 5 upwards/downwards moving bolts, 3 carabiners, one security chain, and one key. The 3 carabiners had to be twisted circularly and 1 key had to be turned in a padlock and the padlock then lifted vertically upwards.

#### Number of movements and efficiency in opening the doors, gates and mechanisms

To open doors or gates (Figs 1 and 3) the animals performed a median of 1 movement (min = 1, max. = 15) in 1 movement sequence (median, min. = 1, max. = 11). The animals’ efficiency in opening doors and gates was 0.9 (median, min. = 0.33, max. = 1).

For opening barred doorways horses performed fewer movements than for opening sliding doors (GLMM: N = 75, SE = 2.89, z = -3.3, p < 0.001) or doors on hinges (GLMM: N = 75, SE = 2.75, z = -3.93, p < 0.001). Thus, horses used the most movement sequences to open barred doorways, fewer for sliding doors (GLMM: N = 75, SE = 2.34, z = -3.14, p = 0.002) and fewer still when the doors were on hinges (GLMM: N = 75, SE = 2.24, z = -3.43, p < 0.001). Individuals differed significantly in the number of movements (GLMM: N = 75, SE = 0.05, z = -2.13, p = 0.03) and number of movement sequences they performed (GLMM: N = 75, SE = 0.04, z = -2.48, p = 0.01) to open barred door and gates.

The efficiency of the horses in opening did not differ between door and gate types (GLMM: N = 75, all p > 0.05) and was very close to the best efficiency possible for humans (efficiency = 1). However, subjects which opened several door and gate types needed fewer movements (GLM: AIC = 395.97, N = 89, z = - 2.23, p = 0.03), fewer movement sequences (GLM: AIC = 287.09, N = 89, z = -1.99, p = 0.05) than horses which opened only one door or gate type.

To open the mechanisms (Figs 2 and 3), individuals performed a median of 3 movements (min = 1, max. = 10) in 2 movement sequences (median, min. = 1, max. = 8). The animals’ efficiency in opening mechanisms was 0.5 (median, min. = 0.1, max. = 1).

**Fig 7:**
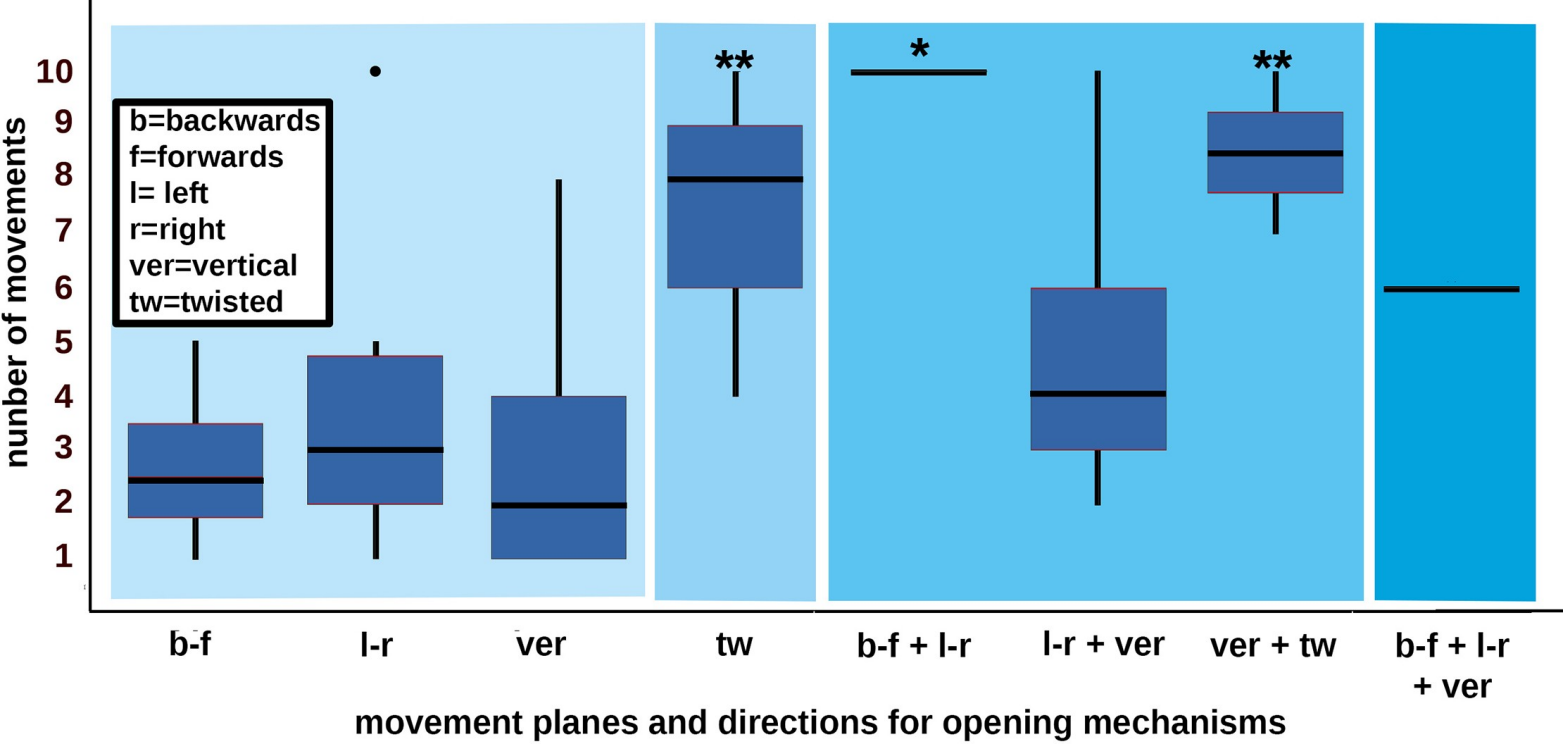
Numbers of movements horses performed for opening mechanisms on different movement planes. The animals needed more movements to open a lock when they had to twist them (median = 8, min. = 4, max. = 10; GLM: N = 89, Std.E. = 1.77, z = 2.59, p = 0.0096), or move them in several directions within one movement plane (left–right, back—forth, vertical) or on several movement planes (linear and twisted): i.e. back or forth and left or right (median = 10, min. = 10, max. = 10; GLM: N = 89, Std.E. = 3.27, z = 2.22, p = 0.03), left or right and vertical (median = 4, min. = 2, max. = 10; GLM: N = 89, Std.E. = 0.89, z = 1.67, p = 0.09), vertical and twisted (median = 8.5, min = 7, max. = 10, GLM: N = 89, Std.E. = 2.22, z = 2.59, p = 0.009), and back or forth and left or right and vertical (median = 6, min. = 6, max. = 6; but this is not significant: GLM: N = 89, Std.E. = 2.59, z = 1.26, p = 0.2), rather than moving them only left or right (median = 3, min. = 1, max. = 10), only back or forth (median = 2.5, min. = 1, max. = 5) or only vertically (median = 2, min. = 1, max. = 8) (GLM: N = 89, all p > 0.05). The boxplots visualize the quartiles of the data per lock type. The box comprises 50% and the lower and upper whisker 25% of the variability each. The dots visualize outliers. ** = p < 0.01 and * = p ≤ 0.05.

Mechanisms differed in the number of movements the horses elicited to open them (GLMM: N = 89, SE = 0.16, z = -2.24, p = 0.02, Fig 6, S3 File), as they tended to differ in length of movement sequence (GLMM: N = 89, SE = 0.16, z = 1.74, p = 0.08). The number of movements the horses used to open the mechanism types were higher when the mechanism had to be opened in two or three rather than in only one movement direction (GLMM: N = 89, SE = 0.5, z = 2.99, p = 0.003, Fig 7). Furthermore, mechanisms which required twisting movements elicited a higher number of movements from the horses (median = 8, min = 4, max. = 10) than mechanisms which could be opened on linear movement planes (median = 2, min = 1, max = 10, GLMM: N = 89, SE = 0.94, z = 3.46, p < 0.001, Fig 7). The number of movements applied to mechanisms which needed twisting movements differed between individuals (GLMM: N = 89, SE = 0.03, z = 4.78, p < 0.001). Furthermore, the animals applied more movements and more movement sequences to open a door or gate when they had to move the mechanism in several directions within one movement plane (Fig 7, statistical data see S3 File). However, the opening efficiency of the horses did not differ between mechanism types, or according to whether horses had to apply different numbers of movement directions or planes (GLMM: N = 89, all p > 0.05).

## Discussion

Crowdsourcing resulted in a large sample of cases of door and gate opening in horses. Most of the horses opened only one door, gate or mechanism type at a single location. However, some individuals opened the same type of door or gate mechanism at several locations, some operated several types of mechanisms, and some were even able to open doors and gates secured with several mechanism types at different positions. These horses seemed to have understood **[4]** and generalized the concept of “locked doors” **[49]**,

In 74 out of 257 cases, other animals in the same stable demonstrated the same behaviour, offering options for learning the manipulation of the same types of locked doors and gates through observation of conspecifics **[19,41]**. In 61 cases, further animals in the stable started displaying the same door and gate opening behaviour shown by the focal animal. Some individual animals therefore may have learned the behaviour through observing conspecifics. However, in general subjects performed equally successfully in opening locked doors and gates no matter whether they could have observed conspecifics or not. Twist locks were an exception: here, horses with no option to observe the behaviour in other horses were more successful in opening the locks. The exact manipulation of twist locks may have been difficult to observe in conspecifics, as they were opened by twisting lips and the head **[17,50]**. In general, we cannot exclude individual trial and error learning as the main mechanism for learning how to open locked doors and gates **[10,14,50]**. Also, horses may have learned to handle the locking devices through observing humans doing so **[20]**; if so, the subjects were innovative in acquiring door opening techniques from observing humans, as they would have to use different body parts and to approach the locking mechanisms from different angles than the observed persons **[17,25,51]**. Interestingly, animals which had prior opportunity to observe the door opening procedure in stable mates were over-represented among horses which remained in a stable even after opening its locked door. This result calls for a follow up study on observational learning of door opening behavior and on potential social learning mechanisms in use by the horses: horses may display door opening behavior as a result of social stimuli or observing other horses or humans, without necessarily reflecting unsatisfactory management conditions **[4,42–45]** or primary goals, but simply for play **[52]**.

A beneficial effect of experience was found: animals which opened more locked door and gate types applied fewer head movements and fewer movement sequences to opening locks than horses which opened only one door or gate type.

Horses favoured their mouth for prehension of door and gate mechanisms, which were, of course, made for human hand use. Similarly, most horses chose to use their mouth when pressing a button to open a feeding apparatus **[19,20]**. In contrast, dogs, cats and chickens have been found to use their claws and paws to open cage locking devices **[10]**, and dogs pulled towels with food out of a container with their paw, no matter whether they observed other dogs using their mouth or paw, or humans using their hands **[53]**.

In our survey, most horses were reported to open doors and gates on hinges, with bars or handles which could be opened on one plane with only a few head movements. However, an impressive number of horses handled more complicated mechanisms, which required movements in more than one plane and specific sequences of actions to be applied. In the main, horses applied similar numbers of movements to those needed by humans to open doors and gates, but twice as many when opening locks. The locks included carabiners, twist mechanisms, keys at doors and padlocks, and electric fence handles; the latter also needed to be handled precisely if possible electric shocks were to be avoided. Horses applied more movements the more complex the mechanism was, but their efficiency, in terms of the number of actions applied compared to the minimum necessary, was similar for opening simple and more complex mechanisms. The range of fastening devices that horses have learnt to open apparently spans the gamut of devices in frequent use in the nations participating in the study: thus, we found no obvious limit to the complexity which horses can learn to master **[4,14,41]**.

Door and gate opening in horses is not generally associated with the quality of human-imposed environmental conditions **[4,43–45]**: cases came from both single and group housing, and where horses were given access to pasture, conspecifics, and roughage equally often. In some cases, a desire for free movement, known to be caused by unsatisfactory management conditions **[4,42–45]** and reported in environmental enrichment studies for several species **[8,54–56]**, may have motivated the reported escape. However, some horses stayed after opening a door or gate and some broke into feed storage rooms even though they had daily access to pasture. In such cases, curiosity, playfulness or a desire for tasty food **[52,57,58]** seem possible motivations. Horse owner and caretaker reports and video documentations indicate that horses do open boxes, feed storage rooms and houses to gain access to preferred food **[19,20,52,58]**, and that they free other group members, as has been reported in rats, which freed caged companions **[9]** and released soaked conspecifics **[59]**.

We found individual differences in the number of movements and the efficiency in dealing with barred doors and gateways and mechanisms which needed twisting movements, which may have been the result of construction variations between the different mechanisms. In addition, the horses may have differed in their past opportunities to practice opening techniques **[14,60–62]**, because horses opening several door and gate types applied less movements to open the locks. The fact that horses which opened feed storage rooms were older and had more access to pasture supports the role of opportunity in learning novel techniques. Nevertheless, individual personality **[51,52]**, especially the horses’ activity and emotionality **[63]**, may also account for differences in manipulative skills.

The low number of reports on trained door opening horses in the present study was not unexpected, as animal escapes may have serious consequences for the animal, the animal owners, caretakers and the environment, for example when they hit the traffic **[5,6]**. As many persons wrote that they tried to prevent their animals from escaping, but were inefficient, we suppose that the number of horses trying but being successfully prevented from opening human locking devices is much higher than reported.

### Limitations and chances of crowdsourcing for collecting data

As with any questionnaire, the present study ran the risk of collecting biased reports **[25,36,37]**. “Owning a clever animal” is undoubtedly seen as desirable by some owners and caretakers **[37]** so that highly motivated respondents are likely to be over-represented in our data **[38]**. Nevertheless, we doubt whether the 333 highly motivated horse owners and caretakers who completed the questionnaires intentionally provided false information in their responses. However, although the people who filmed the actions had tried hard to prevent developing unconscious cues in the first place, they might have unconsciously given off cues that aided the horse at the time of filming or observing. Horses are highly sensitive to human demeanour **[64–67]** and orientate on human attention **[68,69]**, as in the case of the famous “counting” horse Clever Hans **[70]**. Unconscious cues may be inevitable for data raised by crowdsourcing, as even trained persons unconsciously interact with their test animals **[22]**, but we do not see how the cues given while documenting door and gate opening could have helped the horse in developing the behaviour. Future studies may ask for personal data of the animal owners and caretakers to gain more insight into the generalizability of the animal sample, or attempt specifically to collect negative as well as positive data.

## Conclusions

Horses open a far wider range of human-made mechanical devices on doors and gates than previously reported, generally handling the mechanisms with their mouths. Although most horses are confined by simple bolts or handles, and most reports were of opening such devices, a surprising range of fastenings, including carabiners and electric fence handles, proved vulnerable to opening by horses confined by them. Indeed, within the range of locking devices in frequent use for restraining horses, we found no clear cognitive limit to horses’ ability to open them and some evidence for experience improving the horses’ skills. The ability of horses and other ungulates to open human-made fastenings therefore needs to be reconsidered to minimise damage caused by escapes.

## Supporting information

S1 Fig From data request to case selection. a) data request: people were requested to send information on unusual behaviour (general questionnaire; S1 File) and door opening (questionnaire door opening; S2 File) worldwide. b) case documentation: people observed their animals and collected reports, pictures and videos. c1) data transfer: information, pictures and videos on the behaviour and information on the individual animal and its management were reported at the website (https://innovative-behaviour.org) and c2) videos on door opening were published at the internet platform YouTube. d) case selection: the research group selected reported door and gate opening cases in equids from the website and from the internet platform YouTube and deleted questionable data. a) and c1) screen prints were made from the web site (https://innovative-behaviour.org), c2) screen prints from YouTube. Clip arts were either drawn by the author or downloaded from the website https://openclipart.org (i.e. 100% open domain platform).
(PDF)

S1 File Questionnaire “Innovative behaviour in horses”. Please enlarge Pdf for viewing the data.
(PDF)

S2 File Questionnaire “Horses that open doors or gates”. Please enlarge Pdf for viewing the data.
(PDF)

S3 File Statistical data, complete and reduced GLM and GLMM. (PDF)

S1 Table Data survey. Please enlarge Pdf for viewing the data.
(PDF)

S2 Table Data videos. Please enlarge Pdf for viewing the data.
(PDF)

S3 Table Data movement counts videos. Please enlarge Pdf for viewing the data.
(PDF)

S4 Table Data mules and donkeys door opening. Please enlarge Pdf for viewing the data.
(PDF)
