# Supplementary material for: Animal behaviour in a human world: A crowdsourcing study on horses that open door and gate mechanisms
Source: PLoS One. 2019 Jun 26;14(6):e0218954. doi: 10.1371/journal.pone.0218954 (PMC6594629; doi:10.1371/journal.pone.0218954)
Supplement: S1 Fig — a) data request: people were requested to send information on unusual behaviour (general questionnaire; S1 File) and door opening (questionnaire door opening; S2 File) worldwide. b) case documentation: people observed their animals and collected reports, pictures and videos. c1) data transfer: information, pictures and videos on the behaviour and information on the individual animal and its management were reported at the website (https://innovative-behaviour.org) and c2) videos on door opening were published at the internet platform YouTube. d) case selection: the research group selected reported door and gate opening cases in equids from the website and from the internet platform YouTube and deleted questionable data. a) and c1) screen prints were made from the web site (https://innovative-behaviour.org), c2) screen prints from YouTube. Clip arts were either drawn by the author or downloaded from the website https://openclipart.org (i.e. 100% open domain platform). (PDF) [file pone.0218954.s001.pdf]

Supporting information, S1 Fig

Animal behaviour in a human world: A crowd sourcing study on horses that open locked doors and gates

Krueger K. *, Esch L. Byrne R.

* Corresponding author:

Konstanze Krueger,

Konstanze.Krueger@hfwu.de

tel: 0049 7022 201 331

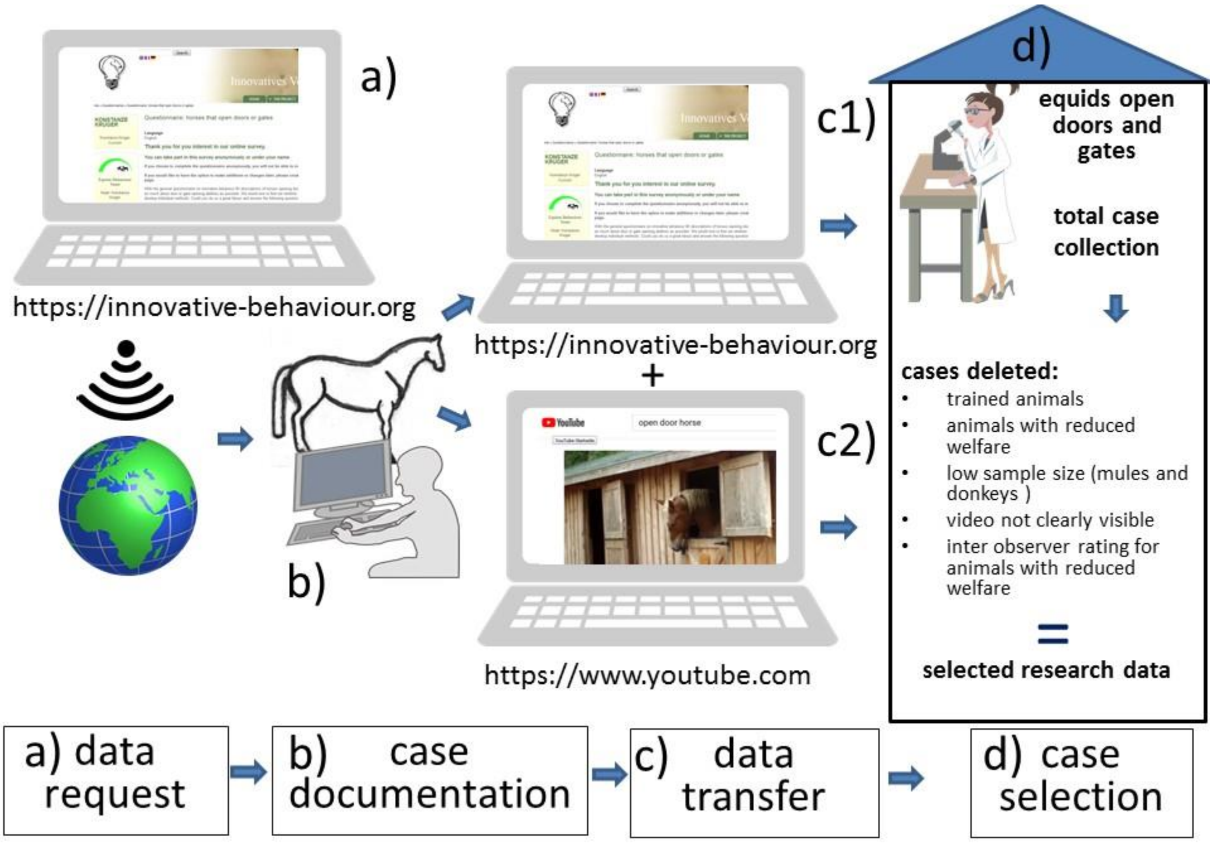

S1 Fig. From Data request to case selection. a) data request: people were requested to send information on unusual behaviour (general questionnaire; S1 File) and door opening (questionnaire door opening; S2 File) worldwide. b) case documentation: people observed their animals and collected reports, pictures and videos. c1) data transfer: information, pictures and videos on the

behaviour and information on the individual animal and its management were reported at the website (https://innovative-behaviour.org) and c2) videos on door opening were published at the internet platform YouTube. d) case selection: the research group selected reported door and gate opening cases in equids from the website and from the internet platform YouTube and deleted questionable data. a) and c1) screen prints were made from the web site (https://innovative-behaviour.org), c2) screen prints from YouTube. Clip arts were either drawn by the author or downloaded from the website https://openclipart.org (i.e. 100% open domain platform).
