# Supplementary material for: Animal behaviour in a human world: A crowdsourcing study on horses that open door and gate mechanisms
Source: PLoS One. 2019 Jun 26;14(6):e0218954. doi: 10.1371/journal.pone.0218954 (PMC6594629; doi:10.1371/journal.pone.0218954)
Supplement: S1 File — Please enlarge Pdf for viewing the data. (PDF) [file pone.0218954.s002.pdf]

Supporting information, S1 File

Animal behaviour in a human world: A crowd sourcing study on horses that open door and gate mechanisms

Krueger K, Esch L, Byrne R

Questionnaire Innovative Behaviour

Language
English

Thank you for you interest in our online survey.

Please create a log-in using the box on the right of this page. This will allow you to change your answers after your questionnaire has been submitted and will enable us to contact you with any further questions or queries. If you do not wish to do this, you can continue without a log-in and complete the questionnaire anonymously.

If your horse does something that is different from behavior generally shown by horses, please tell us about it at the "innovative behavior questionnaire". Please do not use this questionnaire for reports on door or gate opening. You are welcome to tell us about door or gate openers at the questionnaire "open doors or gates". We appreciate your input very much.

Thank you for participating in our online survey.

- The term "horse" is used for all equids (mules, donkeys and zebras) in the following questionnaire. Details of the breed can be given in the breed question or comment field on follow-up pages.
- Please describe the behaviour of **one** horse. If the behaviour is shown by several horses, it can be specified in a later section of the questionnaire.
- Please note, that all questions marked with a *, need to be answered to proceed.

Which style of management does the horse live in? *

- (●) group stabling
- ( ) single box housing

Group stabling

- ( ) Open stable
- ( ) Active stable
- ( ) on pasture

An Open stable is characterized through diversion of functional areas like, feeding, resting and paddock An Active stable is a housing system for a group of horses with automated individual feeding and an incentive to move

Which style of management does the horse live in? *

- ( ) group stabling
- (●) single box housing

Single housing

- ( ) Inside Box
- ( ) Outside Box
- ( ) Paddock Box

Inside Box means a box inside a stable without a paddock or open window to the outside. Outside Box is a box with an open window to the outside. Paddock Box is a box with an attached continously opened paddock.

- ( ) 1-2 times per week
- ( ) 3-4 times per week
- ( ) 5-6 times per week
- ( ) every day

How many roughage (hay, straw, grass) does the horse receive daily? *

- (•) none
- ( ) rationed: no more than 3 kg hay/straw or 3 hours on the pasture
- ( ) rationed: between 3 and 7 kg hay/straw or up to 6 hours on the pasture
- ( ) rationed: more than 7 kg hay/straw or longer than 6hours on the pasture
- ( ) continuously free availibility of roughage or pasture

How often has this behaviour been observed? *

- (•) once
- ( ) 2-10 times
- ( ) 11-20 times
- ( ) more than 20 times
- ( ) daily

Do other horses in the same stabling show this behaviour, too? *

- ( ) no
- ( ) yes, one other horse
- (•) yes, more than one other horse

What exactly does the horse do? *

Which body parts does it use? *

Does it manipulate items *

- (•) yes
- ( ) no
- ( ) unknown

How does it manipulate items

Did the horse appear to be copying something it had seen you, or another person, doing? *

- (•) yes
- ( ) no
- ( ) unknown

Do you react to the described behaviour when you witness it? *

- (x) yes
- ( ) no
- ( ) unknown

How do you react to the described behaviour when you witness it?

Please note:

For a comparable and exact analysis of the horses behaviour the discription should include:

- Under what circumstances and in what situation has the behaviour been shown?
- When does it happen?
- What do you think the behaviour achieves?
- What do you think was the horse's motivation to develop this behaviour?
- Do other horses take part? If so, how many, and which one showed the behaviour first?

Please insert a description of the behaviour you were watching here *

--- Please insert a discription of the behaviour you were watching here ---

Please feel free to attach pictures or clips

Add a new file

Browse no file selected Upload

Files must be less than **10 MB**.

Allowed file types: **gif jpg jpeg png bmp eps tif pict psd pdf avi mov mp3 ogg wav gz rar tar zip mp4 swf flv mkv**.

Name of the horse

anonym

Sex *

- ( ) mare
- ( ) gelding
- ( ) stallion

Age (when observed) *

- Select -

Horse's Breed *

- Select -

e-mail *

Do you have any suggestions or concerns regarding to our project, innovative behaviour in general, or your observations?

I agree *

- to the usage of my information for the project on obeservation and analysis of innovative behaviour in horses at the Nürtingen University (HfWU Nürtingen-Geislingen). I permit, the publication in journals in anonymized form.
- to the usage of my information for the project on obeservation and analysis of innovative behaviour in horses at the Nürtingen University (HfWU Nürtingen-Geislingen).

The Information will not be given to others.

Use of e-mail adress *

- I permit my e-mail adress to be used for further inquiries concerning this project.
- I do not permit my e-mail adress to be used for further inquiries concerning this project.

None of your information will be given to others.
