# Supplementary material for: Animal behaviour in a human world: A crowdsourcing study on horses that open door and gate mechanisms
Source: PLoS One. 2019 Jun 26;14(6):e0218954. doi: 10.1371/journal.pone.0218954 (PMC6594629; doi:10.1371/journal.pone.0218954)
Supplement: S3 File — (PDF) [file pone.0218954.s004.pdf]

Supporting information, S3 File

# Animal behaviour in a human world: A crowd sourcing study on horses that open door and gate mechanisms

Krueger K, Esch L, Byrne R

Complete statistical data, GLM and GLMM

GLMs for subchapters:

**How did animals learn to open door and gate mechanisms (survey N = 333)?**

**Were door and gate openers more often of a certain type (survey N = 333)?**

GLM, effect of the horses' age and options to observe the opening on the number of different door and gate types individual horses were reported to open

```
Call:
glm(formula = nr.of.different.doors.opened ~ age.in.years + other.horse.show.same.behaviour.yes.no, family = poisson(iden
tity), data = Dataset)

Deviance Residuals:
    Min       1Q   Median       3Q      Max
-1.7982  -0.5223  -0.5157   0.2910   1.5781

Coefficients:
                                       Estimate Std. Error z value Pr(>|z|)
(Intercept)                            1.605660   0.165193   9.720   <2e-16 ***
age.in.years                           0.001105   0.013182   0.084    0.933
other.horse.show.same.behaviour.yes.no 0.094009   0.184517   0.509    0.610
---
Signif. codes:  0 '***' 0.001 '**' 0.01 '*' 0.05 '.' 0.1 ' ' 1

(Dispersion parameter for poisson family taken to be 1)
```

```
    Null deviance: 81.447  on 243  degrees of freedom
Residual deviance: 81.181  on 241  degrees of freedom
  (1048329 observations deleted due to missingness)
AIC: 660.88
```

GLM, effect of the horses' age and options to observe the opening on the number of different mechanism types individual horses were reported to open

```
Call:
glm(formula = nr.of.differnt.mechanisms.opened ~ age.in.years + other.horse.show.same.behaviour.yes.no, family = poisson(
identity), data = Dataset)

Deviance Residuals: 
     Min        1Q    Median        3Q       Max  
-0.77757  -0.61269   0.05758   0.21368   2.00541  

Coefficients:
                                       Estimate Std. Error z value Pr(>|z|)    
(Intercept)                            1.648748   0.171073   9.638   <2e-16 ***
age.in.years                           0.009496   0.013892   0.684    0.494    
other.horse.show.same.behaviour.yes.no 0.104751   0.191656   0.547    0.585    
---
Signif. codes:  0 '***' 0.001 '**' 0.01 '*' 0.05 '.' 0.1 ' ' 1

(Dispersion parameter for poisson family taken to be 1)

    Null deviance: 107.35  on 243  degrees of freedom
Residual deviance: 106.64  on 241  degrees of freedom
  (1048329 observations deleted due to missingness)
AIC: 699.6
```

GLM, effect of the horses' age and options to observe the opening on the number of different mechanism positions individual horses were reported to open.

```
Call:
glm(formula = nr.of.mechanism.positions ~ age.in.years + other.horse.show.same.behaviour.yes.no, family = poisson(identit
y), data = Dataset)

Deviance Residuals:
     Min        1Q    Median        3Q       Max
-0.82861  -0.66391  -0.00253   0.14978   1.80235

Coefficients:
                                       Estimate Std. Error z value Pr(>|z|)
(Intercept)                            1.781983   0.175550  10.151   <2e-16 ***
age.in.years                           0.004537   0.014144   0.321    0.748
other.horse.show.same.behaviour.yes.no 0.176222   0.198561   0.887    0.375
---
Signif. codes:  0 '***' 0.001 '**' 0.01 '*' 0.05 '.' 0.1 ' ' 1

(Dispersion parameter for poisson family taken to be 1)

    Null deviance: 111.04  on 244  degrees of freedom
Residual deviance: 110.18  on 242  degrees of freedom
  (1048328 observations deleted due to missingness)
AIC: 717.89
```

GLM, effect of the option to observe the opening on the frequency of opening certain door, gate or mechanism types (full model)

```
Call:
glm(formula = other.horse.show.same.behaviour.yes.no ~ door.on.hinges +
    sliding.doors + barred.door..or.gateway + bolt.sideways +
    bolt.upwards...downwards + carabina + handle + opens.security.chain...rope +
    twist.mechanism, family = binomial(logit), data = Dataset)

Deviance Residuals:
    Min       1Q   Median       3Q      Max
-1.1340  -0.8800  -0.7329   1.2729   2.1814

Coefficients:
                            Estimate Std. Error z value Pr(>|z|)
(Intercept)                 -1.66388    0.61026  -2.727   0.0064 **
door.on.hinges               0.94218    0.66394   1.419   0.1559
sliding.doors               -0.18556    0.45571  -0.407   0.6839
barred.door..or.gateway      0.14699    0.81367   0.181   0.8566
bolt.sideways               -0.02721    0.37631  -0.072   0.9424
bolt.upwards...downwards    -0.24295    0.31880  -0.762   0.4460
carabina                     0.11432    0.44315   0.258   0.7964
handle                       0.24944    0.35233   0.708   0.4790
opens.security.chain...rope  0.52714    0.33953   1.553   0.1205
twist.mechanism             -1.10457    0.51964  -2.126   0.0335 *
---
Signif. codes:  0 '***' 0.001 '**' 0.01 '*' 0.05 '.' 0.1 ' ' 1

(Dispersion parameter for binomial family taken to be 1)

    Null deviance: 306.5  on 253  degrees of freedom
Residual deviance: 295.1  on 244  degrees of freedom
  (1048319 observations deleted due to missingness)
AIC: 315.1
```

GLM, effect of the option to observe the opening on the frequency of opening certain door, gate or mechanism types (reduced model)

```
Call:
glm(formula = other.horse.show.same.behaviour.yes.no ~ twist.mechanism +
    door.on.hinges, family = binomial(logit), data = Dataset)

Deviance Residuals:
    Min       1Q   Median       3Q      Max
-0.9043  -0.9043  -0.6737   1.4777   1.9511

Coefficients:
               Estimate Std. Error z value Pr(>|z|)
(Intercept)     -1.3674     0.3958  -3.455 0.000551 ***
twist.mechanism -1.0591     0.5085  -2.083 0.037252 *
door.on.hinges   0.6844     0.4251   1.610 0.107345
---
Signif. codes:  0 '***' 0.001 '**' 0.01 '*' 0.05 '.' 0.1 ' ' 1

(Dispersion parameter for binomial family taken to be 1)

    Null deviance: 307.19  on 254  degrees of freedom
Residual deviance: 299.97  on 252  degrees of freedom
  (1048318 observations deleted due to missingness)
AIC: 305.97
```

Subchapter:

**What aims did the horses have when they opened doors and gates (survey N = 333)?**

GLM on whether horses walked out of boxes and pastures was affected by the horses' management, their age or options to observe the opening of doors, gates and mechanisms (full model)

```
Call:
glm(formula = stays.in.the.stable ~ access.to.pasture + amount.of.feed +
    single...group.stabling + social.contact + age.in.years +
    other.horses.in.stable.show.same.behaviour, family = binomial(logit),
    data = Dataset)

Deviance Residuals:
    Min       1Q   Median       3Q      Max
-1.2728  -0.9618  -0.7919   1.1696   1.9422

Coefficients:
                                           Estimate Std. Error z value Pr(>|z|)
(Intercept)                                 1.17969    0.66024   1.787   0.0740
access.to.pasture                          -0.48492    0.33964  -1.428   0.1534
amount.of.feed                              0.02519    0.28936   0.087   0.9306
single...group.stabling                     0.14357    0.92820   0.155   0.8771
social.contact                             -0.58776    0.91201  -0.644   0.5193
age.in.years                               -0.01990    0.02267  -0.878   0.3801
other.horses.in.stable.show.same.behaviour -0.44718    0.23983  -1.865   0.0622

---
Signif. codes:  0 '***' 0.001 '**' 0.01 '*' 0.05 '.' 0.1 ' ' 1

(Dispersion parameter for binomial family taken to be 1)

    Null deviance: 304.06  on 229  degrees of freedom
Residual deviance: 291.42  on 223  degrees of freedom
  (1048343 observations deleted due to missingness)
AIC: 305.42
```

GLM on whether horses walked out of boxes and pastures was affected by the horses’ management, their age or options to observe the opening of doors, gates and mechanisms (reduced model)

```
Call:
glm(formula = stays.in.the.stable ~ access.to.pasture + amount.of.feed +
    age.in.years + other.horses.in.stable.show.same.behaviour,
    family = binomial(logit), data = Dataset)

Deviance Residuals:
    Min       1Q   Median       3Q      Max
-1.2265  -0.9420  -0.7773   1.2097   1.8930

Coefficients:
                                           Estimate Std. Error z value Pr(>|z|)
(Intercept)                                 0.88246    0.61420   1.437   0.1508
access.to.pasture                          -0.71616    0.28487  -2.514   0.0119 *
amount.of.feed                              0.01178    0.28694   0.041   0.9673
age.in.years                               -0.02115    0.02248  -0.941   0.3466
other.horses.in.stable.show.same.behaviour -0.43581    0.23893  -1.824   0.0681 .
---
Signif. codes:  0 '***' 0.001 '**' 0.01 '*' 0.05 '.' 0.1 ' ' 1

(Dispersion parameter for binomial family taken to be 1)

    Null deviance: 304.06  on 229  degrees of freedom
Residual deviance: 293.36  on 225  degrees of freedom
  (1048343 observations deleted due to missingness)
AIC: 303.36
```

GLM on whether horses run around freely without any restriction after opening doors, gates or mechanisms was affected by the horses’ management, their age or options to observe the door, gate and mechanism opening (full model)

```
Call:
glm(formula = runs.around.freely ~ access.to.pasture + amount.of.feed +
    single...group.stabling + social.contact + age.in.years +
    other.horses.in.stable.show.same.behaviour, family = binomial(cloglog),
    data = Dataset)

Deviance Residuals:
    Min       1Q   Median       3Q      Max
-2.1683   0.5341   0.6273   0.7061   0.8221

Coefficients:
                                            Estimate Std. Error z value Pr(>|z|)
(Intercept)                                -0.115785   0.383897  -0.302    0.763
access.to.pasture                           0.161503   0.199532   0.809    0.418
amount.of.feed                              0.099819   0.167627   0.595    0.552
single...group.stabling                     0.117131   0.529239   0.221    0.825
social.contact                             -0.056468   0.522798  -0.108    0.914
age.in.years                                0.008164   0.013027   0.627    0.531
other.horses.in.stable.show.same.behaviour  0.126552   0.133171   0.950    0.342

(Dispersion parameter for binomial family taken to be 1)

    Null deviance: 235.63  on 235  degrees of freedom
Residual deviance: 232.11  on 229  degrees of freedom
  (1048337 observations deleted due to missingness)
AIC: 246.11
```

GLM on whether horses run around freely without any restriction after opening doors, gates or mechanisms was affected by the horses' management, their age or options to observe the door, gate and mechanism opening (reduced model)

```
Call:
glm(formula = runs.around.freely ~ other.horses.in.stable.show.same.behaviour +
    age.in.years, family = binomial(cloglog), data = Dataset)

Deviance Residuals:
    Min       1Q   Median       3Q      Max
-2.0503   0.5588   0.6481   0.6889   0.7198

Coefficients:
                                           Estimate Std. Error z value Pr(>|z|)
(Intercept)                                0.386947   0.160585   2.410    0.016 *
other.horses.in.stable.show.same.behaviour 0.116860   0.131720   0.887    0.375
age.in.years                               0.006789   0.012842   0.529    0.597
---
Signif. codes:  0 '***' 0.001 '**' 0.01 '*' 0.05 '.' 0.1 ' ' 1

(Dispersion parameter for binomial family taken to be 1)

    Null deviance: 238.26  on 241  degrees of freedom
Residual deviance: 237.17  on 239  degrees of freedom
  (1048331 observations deleted due to missingness)
AIC: 243.17
```

GLM on whether horses go into other horses boxes after opening doors, gates or mechanisms was affected by the horses' management, their age or options to observe the door, gate and mechanism opening (full model)

```
Call:
glm(formula = goes.into.other.horses.boxes ~ access.to.pasture +
    amount.of.feed + single...group.stabling + social.contact +
    age.in.years + other.horse.show.same.behaviour.yes.no, family = binomial(cloglog),
    data = Dataset)

Deviance Residuals:
    Min       1Q   Median       3Q      Max
-0.8964  -0.7400  -0.6145  -0.5557   1.9726

Coefficients:
                                        Estimate Std. Error z value Pr(>|z|)
(Intercept)                             -2.34785    0.69975  -3.355 0.000793 ***
access.to.pasture                        0.41475    0.35966   1.153 0.248835
amount.of.feed                          -0.05054    0.29729  -0.170 0.864997
single...group.stabling                 15.16555  911.49983   0.017 0.986725
social.contact                         -15.01804  911.49985  -0.016 0.986854
age.in.years                             0.00432    0.02265   0.191 0.848704
other.horse.show.same.behaviour.yes.no   0.30155    0.30797   0.979 0.327511
---
Signif. codes:  0 '***' 0.001 '**' 0.01 '*' 0.05 '.' 0.1 ' ' 1

(Dispersion parameter for binomial family taken to be 1)

    Null deviance: 238.26  on 229  degrees of freedom
Residual deviance: 228.40  on 223  degrees of freedom
  (1048343 observations deleted due to missingness)
AIC: 242.4
```

GLM on whether horses go into other horses boxes after opening doors, gates or mechanisms was affected by the horses' management, their age or options to observe the door, gate and mechanism opening (reduced model)

```
Call:
glm(formula = goes.into.other.horses.boxes ~ access.to.pasture +
    single...group.stabling + social.contact + age.in.years,
    family = binomial(cloglog), data = Dataset)

Deviance Residuals:
    Min       1Q   Median       3Q      Max
-0.8167  -0.8058  -0.6072  -0.5977   1.9214

Coefficients:
                         Estimate Std. Error z value Pr(>|z|)
(Intercept)             -2.280999   0.533277  -4.277 1.89e-05 ***
access.to.pasture        0.499692   0.320859   1.557    0.119
single...group.stabling  0.161787   0.421949   0.383    0.701
social.contact          -0.068587   0.473960  -0.145    0.885
age.in.years            -0.001491   0.019093  -0.078    0.938
---
Signif. codes:  0 '***' 0.001 '**' 0.01 '*' 0.05 '.' 0.1 ' ' 1

(Dispersion parameter for binomial family taken to be 1)

    Null deviance: 320.62  on 298  degrees of freedom
Residual deviance: 315.89  on 294  degrees of freedom
  (1048274 observations deleted due to missingness)
AIC: 325.89
```

GLM on whether horses free other horses after opening doors, gates or mechanisms was affected by the horses' management, their age or options to observe the door, gate and mechanism opening (full model)

```
Call:
glm(formula = frees.other.horses ~ access.to.pasture + amount.of.feed +
    single...group.stabling + social.contact + age.in.years +
    other.horses.in.stable.show.same.behaviour, family = binomial(logit),
    data = Dataset)

Deviance Residuals:
    Min       1Q   Median       3Q      Max
-1.0234  -0.8560  -0.7316   1.3980   2.0314

Coefficients:
                                            Estimate Std. Error z value Pr(>|z|)
(Intercept)                                -2.073309   0.689780  -3.006  0.00265 **
access.to.pasture                           0.168940   0.359672   0.470  0.63857
amount.of.feed                              0.465077   0.296478   1.569  0.11672
single...group.stabling                     1.250859   1.154576   1.083  0.27863
social.contact                             -1.136695   1.141644  -0.996  0.31941
age.in.years                                0.009918   0.022921   0.433  0.66523
other.horses.in.stable.show.same.behaviour -0.044661   0.238289  -0.187  0.85133
---
Signif. codes:  0 '***' 0.001 '**' 0.01 '*' 0.05 '.' 0.1 ' ' 1

(Dispersion parameter for binomial family taken to be 1)

    Null deviance: 282.74  on 234  degrees of freedom
Residual deviance: 277.53  on 228  degrees of freedom
  (1048338 observations deleted due to missingness)
AIC: 291.53
```

GLM on whether horses free other horses after opening doors, gates or mechanisms was affected by the horses' management, their age or options to observe the door, gate and mechanism opening (reduced model)

```
Call:
glm(formula = frees.other.horses ~ access.to.pasture + amount.of.feed +
    age.in.years + other.horse.show.same.behaviour.yes.no, family = binomial(logit),
    data = Dataset)

Deviance Residuals:
    Min       1Q   Median       3Q      Max
-1.0014  -0.8580  -0.7287   1.4008   1.7753

Coefficients:
                                        Estimate Std. Error z value Pr(>|z|)
(Intercept)                            -1.992694   0.650757  -3.062   0.0022 **
access.to.pasture                       0.261333   0.294288   0.888   0.3745
amount.of.feed                          0.442329   0.294940   1.500   0.1337
age.in.years                            0.008226   0.022833   0.360   0.7186
other.horse.show.same.behaviour.yes.no -0.096200   0.326813  -0.294   0.7685
---
Signif. codes:  0 '***' 0.001 '**' 0.01 '*' 0.05 '.' 0.1 ' ' 1

(Dispersion parameter for binomial family taken to be 1)

    Null deviance: 282.74  on 234  degrees of freedom
Residual deviance: 278.94  on 230  degrees of freedom
  (1048338 observations deleted due to missingness)
AIC: 288.94
```

GLM on whether horses go into feed chambers after opening doors, gates or mechanisms was affected by the horses' management, their age or options to observe the door, gate and mechanism opening (full model)

```
Call:
glm(formula = goes.into.feed.or.other.rooms ~ access.to.pasture +
    amount.of.feed + single...group.stabling + social.contact +
    age.in.years + other.horse.show.same.behaviour.yes.no, family = binomial(logit), data = Dataset)


Deviance Residuals:
    Min       1Q   Median       3Q      Max
-1.4058  -0.9781  -0.8033   1.2627   1.7067

Coefficients:
                                       Estimate Std. Error z value Pr(>|z|)
(Intercept)                            -2.08961    0.65974  -3.167  0.00154 **
access.to.pasture                       0.44870    0.33430   1.342  0.17953
amount.of.feed                          0.10734    0.28182   0.381  0.70328
single...group.stabling                 0.09587    0.81098   0.118  0.90590
social.contact                          0.19894    0.80888   0.246  0.80572
age.in.years                            0.03035    0.02171   1.398  0.16214
other.horse.show.same.behaviour.yes.no -0.13434    0.31087  -0.432  0.66564
---
Signif. codes:  0 '***' 0.001 '**' 0.01 '*' 0.05 '.' 0.1 ' ' 1

(Dispersion parameter for binomial family taken to be 1)

    Null deviance: 309.76  on 234  degrees of freedom
Residual deviance: 301.34  on 228  degrees of freedom
  (1048338 observations deleted due to missingness)
AIC: 315.34
```

GLM on whether horses go into feed chambers after opening doors, gates or mechanisms was affected by the horses' management, their age or options to observe the door, gate and mechanism opening (reduced model)

```
Call:
glm(formula = goes.into.feed.or.other.rooms ~ access.to.pasture +
    age.in.years + other.horse.show.same.behaviour.yes.no, family = binomial(logit),  data = Dataset)

Deviance Residuals:
    Min       1Q   Median       3Q      Max
-1.3697  -0.9916  -0.7935   1.2887   1.6948


Coefficients:
                                       Estimate Std. Error z value Pr(>|z|)
(Intercept)                            -1.83455    0.50376  -3.642 0.000271 ***
access.to.pasture                       0.66123    0.27467   2.407 0.016068 *
age.in.years                            0.03178    0.02155   1.475 0.140310
other.horse.show.same.behaviour.yes.no -0.15010    0.30752  -0.488 0.625482
---
Signif. codes:  0 '***' 0.001 '**' 0.01 '*' 0.05 '.' 0.1 ' ' 1

(Dispersion parameter for binomial family taken to be 1)

    Null deviance: 312.51  on 237  degrees of freedom
Residual deviance: 304.21  on 234  degrees of freedom
  (1048335 observations deleted due to missingness)
AIC: 312.21
```

GLMs and GLMMs for subchapter:

**Number of movements and efficiency in opening the doors, gates and mechanisms**

GLMMs,  nr. of movements, nr. of movement sequences and efficiency in opening door and gate types

```
glm(formula = nr.of.movements ~ door_gate_type_num/ID, family = poisson(identity), data = Dataset)

Deviance Residuals:
     Min        1Q    Median        3Q       Max
-1.58217  -0.26435  -0.15017  -0.03146   3.10756

Coefficients:
                       Estimate Std. Error z value Pr(>|z|)
(Intercept)           -0.975879   0.453776  -2.151   0.0315 *
door_gate_type_num     2.315510   0.436958   5.299 1.16e-07 ***
door_gate_type_num:ID -0.004382   0.004488  -0.976   0.3289
---
Signif. codes:  0 '***' 0.001 '**' 0.01 '*' 0.05 '.' 0.1 ' ' 1

(Dispersion parameter for poisson family taken to be 1)

    Null deviance: 95.967  on 83  degrees of freedom
Residual deviance: 36.857  on 81  degrees of freedom
AIC: 230.94
```

```
glm(formula = nr.of.movements ~ door_gate_type/ID, family = poisson(identity),
    data = Dataset)
Deviance Residuals:
    Min       1Q   Median       3Q      Max
-1.5029  -0.1798  -0.1730  -0.1682   2.6057
Coefficients:
                                    Estimate Std. Error z value Pr(>|z|)
(Intercept)                        11.973683   2.735275   4.378 1.20e-05 ***
door_gate_type[T.hinge door/gate] -10.779320   2.745830  -3.926 8.65e-05 ***
door_gate_type[T.slide door]       -9.550826   2.891138  -3.303 0.000955 ***
door_gate_typebarred door/gate:ID  -0.117572   0.055171  -2.131 0.033085 *
door_gate_typehinge door/gate:ID   -0.000218   0.005083  -0.043 0.965792
door_gate_typeslide door:ID        -0.011463   0.020299  -0.565 0.572265
---
Signif. codes:  0 '***' 0.001 '**' 0.01 '*' 0.05 '.' 0.1 ' ' 1

(Dispersion parameter for poisson family taken to be 1)

    Null deviance: 95.967  on 83  degrees of freedom
Residual deviance: 23.206  on 78  degrees of freedom
AIC: 223.29

glm(formula = nr.of.movement.sequences ~ door_gate_type_num/ID, family = poisson(identity), data = Dataset)

Deviance Residuals:
     Min        1Q    Median        3Q       Max
-1.28004  -0.10870  -0.02518   0.10233   3.07632
Coefficients:
                       Estimate Std. Error z value Pr(>|z|)
(Intercept)           -0.133785   0.368401  -0.363 0.716492
door_gate_type_num     1.278293   0.351218   3.640 0.000273 ***
door_gate_type_num:ID -0.004108   0.003976  -1.033 0.301619
---
Signif. codes:  0 '***' 0.001 '**' 0.01 '*' 0.05 '.' 0.1 ' ' 1

(Dispersion parameter for poisson family taken to be 1)

    Null deviance: 49.919  on 83  degrees of freedom
Residual deviance: 24.265  on 81  degrees of freedom
AIC: 204.3
```

```
glm(formula = nr.of.movement.sequences ~ door_gate_type/ID, family = poisson(identity),
    data = Dataset)
Deviance Residuals:
   Min      1Q  Median      3Q     Max
-1.961   0.000   0.000   0.000   2.079
Coefficients:
                                    Estimate Std. Error z value Pr(>|z|)
(Intercept)                        8.683e+00  2.230e+00   3.893 9.88e-05 ***
door_gate_type[T.hinge door/gate] -7.683e+00  2.241e+00  -3.428 0.000607 ***
door_gate_type[T.slide door]      -7.337e+00  2.337e+00  -3.140 0.001692 **
door_gate_typebarred door/gate:ID -1.044e-01  4.216e-02  -2.476 0.013303 *
door_gate_typehinge door/gate:ID   3.195e-17  4.669e-03   0.000 1.000000
door_gate_typeslide door:ID       -6.379e-03  1.513e-02  -0.422 0.673320
---
Signif. codes:  0 '***' 0.001 '**' 0.01 '*' 0.05 '.' 0.1 ' ' 1

(Dispersion parameter for poisson family taken to be 1)

    Null deviance: 49.919  on 83  degrees of freedom
Residual deviance: 11.082  on 78  degrees of freedom
AIC: 197.11

glm(formula = X..of.movements.neccessary ~ door_gate_type_num/ID,
    family = gaussian(identity), data = Dataset)
Deviance Residuals:
     Min        1Q    Median        3Q       Max
-0.72812   0.06440   0.06775   0.07259   0.35489
Coefficients:
                        Estimate Std. Error t value Pr(>|t|)
(Intercept)            1.1314532  0.0552635  20.474  < 2e-16 ***
door_gate_type_num    -0.1953484  0.0478413  -4.083 0.000104 ***
door_gate_type_num:ID -0.0001353  0.0006879  -0.197 0.844534
---
Signif. codes:  0 '***' 0.001 '**' 0.01 '*' 0.05 '.' 0.1 ' ' 1
(Dispersion parameter for gaussian family taken to be 0.04185601)

    Null deviance: 4.3815  on 83  degrees of freedom
Residual deviance: 3.3903  on 81  degrees of freedom
AIC: -23.249
```

```
glm(formula = X..of.movements.neccessary ~ door_gate_type/ID,
    family = gaussian(identity), data = Dataset)

Deviance Residuals:
     Min        1Q    Median        3Q       Max
-0.74955   0.04052   0.05390   0.07419   0.39496

Coefficients:
                                    Estimate Std. Error t value Pr(>|t|)
(Intercept)                        0.8195934  0.1738116   4.715 1.04e-05 ***
door_gate_type[T.hinge door/gate]  0.0960041  0.1793260   0.535    0.594
door_gate_type[T.slide door]      -0.1945879  0.2133977  -0.912    0.365
door_gate_typebarred door/gate:ID -0.0048815  0.0040048  -1.219    0.227
door_gate_typehinge door/gate:ID   0.0005755  0.0009346   0.616    0.540
door_gate_typeslide door:ID       -0.0003767  0.0028270  -0.133    0.894
---
Signif. codes:  0 '***' 0.001 '**' 0.01 '*' 0.05 '.' 0.1 ' ' 1

(Dispersion parameter for gaussian family taken to be 0.04007749)

    Null deviance: 4.3815  on 83  degrees of freedom
Residual deviance: 3.1260  on 78  degrees of freedom
AIC: -24.066
```

GLMs, nr. of movements, nr. of movement sequences and efficiency in opening mechanism types ~ nr. of door and gates types opened, nr. of lock types opened, nr. of lock positions

```
Call:
glm(formula = nr.of.movements.for..lock.opening ~ nr.diff.doors....barrier.types +
    nr.diff.locks.types + nr_lock_.positions + lock.type, family = poisson(identity),
    data = Dataset)

Deviance Residuals:
     Min        1Q    Median        3Q       Max
-2.11906  -1.08595  -0.01119   0.50635   2.54412

Coefficients:
                                          Estimate Std. Error z value Pr(>|z|)
(Intercept)                                3.85826    1.62992   2.367  0.01793 *
nr.diff.doors....barrier.types            -4.27363    1.80133  -2.372  0.01767 *
nr.diff.locks.types                        2.34477    1.47854   1.586  0.11277
nr_lock_.positions                         0.07061    0.34210   0.206  0.83648
lock.type[T.bosd]                          1.95157    1.44535   1.350  0.17694
lock.type[T.boupdo]                        2.81746    1.52225   1.851  0.06419 .
lock.type[T.carabiner]                     7.96482    2.65124   3.004  0.00266 **
lock.type[T.carabiner on chain / no door]  2.00000    2.44949   0.816  0.41422
lock.type[T.ehandle]                       1.87665    1.75950   1.067  0.28616
lock.type[T.handle]                        0.56352    1.50242   0.375  0.70761
lock.type[T.key]                           1.31046    4.32948   0.303  0.76213
lock.type[T.secch]                         5.00000    3.00000   1.667  0.09558 .
---
Signif. codes:  0 '***' 0.001 '**' 0.01 '*' 0.05 '.' 0.1 ' ' 1

(Dispersion parameter for poisson family taken to be 1)

    Null deviance: 133.747  on 88  degrees of freedom
Residual deviance:  95.642  on 77  degrees of freedom
  (12 observations deleted due to missingness)
AIC: 395.97
```

```
Call:

glm(formula = Nr.ordered.sequence.lock.opening ~ nr.diff.doors....barrier.types + 
    nr.diff.locks.types + nr_lock_.positions + lock.type, family = poisson(identity), 
    data = Dataset)

Deviance Residuals: 
     Min        1Q    Median        3Q       Max  
-1.21198  -0.80143  -0.01997   0.35521   2.25189  

Coefficients:
                                          Estimate Std. Error z value Pr(>|z|)  
(Intercept)                              2.036e+00  1.188e+00   1.714   0.0866 .
nr.diff.doors....barrier.types          -2.727e+00  1.385e+00  -1.968   0.0490 *
nr.diff.locks.types                      1.619e+00  1.125e+00   1.439   0.1500  
nr_lock_.positions                       7.197e-02  2.574e-01   0.280   0.7798  
lock.type[T.bosd]                        1.028e+00  1.023e+00   1.006   0.3146  
lock.type[T.boupdo]                      1.359e+00  1.075e+00   1.264   0.2064  
lock.type[T.carabiner]                   2.449e+00  1.661e+00   1.474   0.1404  
lock.type[T.carabiner on chain / no door] -4.140e-16  1.414e+00   0.000   1.0000  
lock.type[T.ehandle]                     1.672e+00  1.314e+00   1.273   0.2031  
lock.type[T.handle]                      5.877e-01  1.076e+00   0.546   0.5850  
lock.type[T.key]                        -2.238e+00  2.839e+00  -0.788   0.4305  
lock.type[T.secch]                       1.000e+00  1.732e+00   0.577   0.5637  
---
Signif. codes:  0 '***' 0.001 '**' 0.01 '*' 0.05 '.' 0.1 ' ' 1

(Dispersion parameter for poisson family taken to be 1)

    Null deviance: 62.655  on 88  degrees of freedom
Residual deviance: 49.548  on 77  degrees of freedom
  (12 observations deleted due to missingness)
AIC: 297.01
```

```
Call:
glm(formula = Nr.ordered.sequence.lock.opening ~ nr.diff.doors....barrier.types +
    nr.diff.locks.types, family = poisson(identity), data = Dataset)

Deviance Residuals:
     Min        1Q    Median        3Q       Max
-0.90228  -0.85516  -0.07943   0.57255   2.17819

Coefficients:
                               Estimate Std. Error z value Pr(>|z|)
(Intercept)                      3.0860     0.5209   5.925 3.13e-09 ***
nr.diff.doors....barrier.types  -1.6947     0.7897  -2.146   0.0319 *
nr.diff.locks.types              0.7231     0.6881   1.051   0.2933
---
Signif. codes:  0 '***' 0.001 '**' 0.01 '*' 0.05 '.' 0.1 ' ' 1

(Dispersion parameter for poisson family taken to be 1)

    Null deviance: 62.655  on 88  degrees of freedom
Residual deviance: 57.626  on 86  degrees of freedom
  (12 observations deleted due to missingness)
AIC: 287.09
```

```
Call:
glm(formula = X..neccessarily.needed ~ nr.diff.doors....barrier.types + 
    nr.diff.locks.types + nr_lock_.positions + lock.type, family = poisson(identity), 
    data = Dataset)

Deviance Residuals: 
    Min       1Q   Median       3Q      Max  
-0.7251  -0.2949  -0.1284   0.1853   0.9245  

Coefficients:
                                          Estimate Std. Error z value Pr(>|z|)
(Intercept)                                0.19736    0.85884   0.230    0.818
nr.diff.doors....barrier.types             0.31130    0.74272   0.419    0.675
nr.diff.locks.types                        0.04026    0.44216   0.091    0.927
nr_lock_.positions                        -0.04893    0.16815  -0.291    0.771
lock.type[T.bosd]                          0.02955    0.71558   0.041    0.967
lock.type[T.boupdo]                       -0.16434    0.72526  -0.227    0.821
lock.type[T.carabiner]                    -0.26133    0.78390  -0.333    0.739
lock.type[T.carabiner on chain / no door] -0.25000    0.86603  -0.289    0.773
lock.type[T.ehandle]                       0.40476    0.85954   0.471    0.638
lock.type[T.handle]                        0.09634    0.75022   0.128    0.898
lock.type[T.key]                          -0.45552    1.18617  -0.384    0.701
lock.type[T.secch]                        -0.21000    0.88882  -0.236    0.813

(Dispersion parameter for poisson family taken to be 1)

    Null deviance: 16.812  on 88  degrees of freedom
Residual deviance: 13.441  on 77  degrees of freedom
  (12 observations deleted due to missingness)
AIC: Inf
```

GLMMs, nr. of movements, nr. of movement sequences and efficiency in opening mechanism types

```
Call:
glm(formula = nr.of.movements.for..mechanism.opening ~ (lock_type_num/ID) + 
    (head..mouth.movement.opening/ID) + (nr.movement.directions/ID), 
    family = poisson(identity), data = Dataset)

Deviance Residuals: 
    Min       1Q   Median       3Q      Max  
-2.0197  -0.8367  -0.2552   0.5047   2.6918  

Coefficients:
                                                 Estimate Std. Error z value Pr(>|z|)    
(Intercept)                                      2.708309   0.724460   3.738 0.000185 ***
mechanism_type_num                              -0.362198   0.159578  -2.270 0.023224 *  
head..mouth.movement.opening[T.linear-twist]    23.612574  25.220561   0.936 0.349148    
head..mouth.movement.opening[T.twist]           -3.177077   0.930686  -3.414 0.000641 ***
nr.movement.directions                           1.492446   0.496339   3.007 0.002639 ** 
mechanism_type_num:ID                            0.004688   0.003618   1.296 0.195105    
ID:head..mouth.movement.opening[T.linear-twist] -0.300291   0.413012  -0.727 0.467181    
ID:head..mouth.movement.opening[T.twist]         0.139555   0.029226   4.775  1.8e-06 ***
ID:nr.movement.directions                       -0.019668   0.010697  -1.839 0.065961 .  
---
Signif. codes:  0 '***' 0.001 '**' 0.01 '*' 0.05 '.' 0.1 ' ' 1

(Dispersion parameter for poisson family taken to be 1)

    Null deviance: 149.377  on 94  degrees of freedom
Residual deviance:  97.825  on 86  degrees of freedom
  (2 observations deleted due to missingness)
AIC: 402.48
```

```
Call:
glm(formula = Nr.ordered.sequences.for.mechanism.opening ~ (lock_type_num/ID) +
    (head..mouth.movement.opening/ID) + (nr.movement.directions/ID),
    family = poisson(identity), data = Dataset)

Deviance Residuals:
    Min       1Q   Median       3Q      Max
-1.4143  -0.7026  -0.2556   0.3326   2.4172

Coefficients:
                                                Estimate Std. Error z value Pr(>|z|)
(Intercept)                                     1.451892   0.555263   2.615  0.00893 **
mechanism_type_num                              0.281707   0.156018   1.806  0.07098 .
head..mouth.movement.opening[T.linear-twist]   16.174285  16.528793   0.979  0.32780
head..mouth.movement.opening[T.twist]          -1.306394   1.013064  -1.290  0.19721
nr.movement.directions                          0.468726   0.368813   1.271  0.20376
mechanism_type_num:ID                          -0.002591   0.003267  -0.793  0.42767
ID:head..mouth.movement.opening[T.linear-twist] -0.245223   0.265294  -0.924  0.35531
ID:head..mouth.movement.opening[T.twist]        0.037015   0.025327   1.461  0.14388
ID:nr.movement.directions                      -0.008287   0.007806  -1.062  0.28844
---
Signif. codes:  0 '***' 0.001 '**' 0.01 '*' 0.05 '.' 0.1 ' ' 1

(Dispersion parameter for poisson family taken to be 1)

    Null deviance: 86.871  on 94  degrees of freedom
Residual deviance: 71.357  on 86  degrees of freedom
  (2 observations deleted due to missingness)
AIC: 333.58
```

```
Call:
glm(formula = X...movements.neccessarily.needed ~ (lock_type_num/ID) +
    (head..mouth.movement.opening/ID) + (nr.movement.directions/ID),
    family = gaussian(identity), data = Dataset)

Deviance Residuals:
     Min        1Q    Median        3Q       Max
-0.60612  -0.22220  -0.03195   0.21471   0.57758

Coefficients:
                                                  Estimate Std. Error t value Pr(>|t|)
(Intercept)                                      0.3367510  0.1123132   2.998 0.003549 **
mechanism_type_num                               0.0989886  0.0259074   3.821 0.000251 ***
head..mouth.movement.opening[T.linear-twist]    -0.6230894  2.4831144  -0.251 0.802466
head..mouth.movement.opening[T.twist]            0.5770972  0.2376006   2.429 0.017229 *
nr.movement.directions                          -0.0282983  0.0728242  -0.389 0.698544
mechanism_type_num:ID                           -0.0013774  0.0005862  -2.350 0.021069 *
ID:head..mouth.movement.opening[T.linear-twist]  0.0021198  0.0412486   0.051 0.959133
ID:head..mouth.movement.opening[T.twist]        -0.0179541  0.0054987  -3.265 0.001572 **
ID:nr.movement.directions                        0.0035179  0.0015684   2.243 0.027470 *
---
Signif. codes:  0 '***' 0.001 '**' 0.01 '*' 0.05 '.' 0.1 ' ' 1

(Dispersion parameter for gaussian family taken to be 0.0843339)

    Null deviance: 10.0530  on 94  degrees of freedom
Residual deviance:  7.2527  on 86  degrees of freedom
  (2 observations deleted due to missingness)
AIC: 45.211
```

GLM, Figure 7

```
Call:
glm(formula = nr.of.movements.for..mechanism.opening ~ lock.type,
    family = poisson(identity), data = Dataset)

Deviance Residuals:
    Min       1Q   Median       3Q      Max
-1.7889  -0.9206  -0.1201   0.4905   2.5511

Coefficients:
                                          Estimate Std. Error z value Pr(>|z|)
(Intercept)                                 1.1250     0.3750   3.000  0.00270 **
mechanism.type[T.bosd]                           2.8568     0.4615   6.190 6.03e-10 ***
mechanism.type[T.boupdo]                         2.8083     0.6347   4.425 9.66e-06 ***
mechanism.type[T.carabiner]                      8.8750     2.2673   3.914 9.06e-05 ***
mechanism.type[T.carabiner on chain / no door]   2.8750     2.0349   1.413  0.15769
mechanism.type[T.ehandle]                        2.8750     0.9699   2.964  0.00303 **
mechanism.type[T.handle]                         1.0972     0.6225   1.763  0.07798 .
mechanism.type[T.key]                            6.8750     2.8532   2.410  0.01597 *
mechanism.type[T.secch]                          5.8750     2.6722   2.199  0.02791 *
---
Signif. codes:  0 '***' 0.001 '**' 0.01 '*' 0.05 '.' 0.1 ' ' 1

(Dispersion parameter for poisson family taken to be 1)

    Null deviance: 153.33  on 96  degrees of freedom
Residual deviance: 105.35  on 88  degrees of freedom
AIC: 414.62
```

GLM, Figure 8

```
Call:
glm(formula = nr.of.movements.for..mechanism.opening ~ movement..mechanism..door,
    family = poisson(identity), data = Dataset)

Deviance Residuals:
    Min       1Q   Median       3Q      Max
-1.5967  -1.2046  -0.4639   0.7185   2.8026

Coefficients:
                                                       Estimate Std. Error z value Pr(>|z|)
(Intercept)                                             2.75000    0.82916   3.317 0.000911 ***
movement..mechanism..door[T.left-right]                      0.78846    0.97964   0.805 0.420906
movement..mechanism..door[T.left-right- forw-back-vertical]  3.25000    2.58602   1.257 0.208841
movement..mechanism..door[T.left-right-forw-back]           7.25000    3.26917   2.218 0.026576 *
movement..mechanism..door[T.left-right-vertical]            1.50000    0.89753   1.671 0.094671 .
movement..mechanism..door[T.twist]                          4.58333    1.76973   2.590 0.009602 **
movement..mechanism..door[T.vertical]                      -0.02027    0.87251  -0.023 0.981465
movement..mechanism..door[T.vertical-twist]                 5.75000    2.22205   2.588 0.009662 **
---
Signif. codes:  0 '***' 0.001 '**' 0.01 '*' 0.05 '.' 0.1 ' ' 1

(Dispersion parameter for poisson family taken to be 1)

    Null deviance: 153.33  on 96  degrees of freedom
Residual deviance: 113.27  on 89  degrees of freedom
AIC: 420.54
```
