# Supplementary material for: Animal behaviour in a human world: A crowdsourcing study on horses that open door and gate mechanisms
Source: PLoS One. 2019 Jun 26;14(6):e0218954. doi: 10.1371/journal.pone.0218954 (PMC6594629; doi:10.1371/journal.pone.0218954)
Supplement: S1 Table — Please enlarge Pdf for viewing the data. (PDF) [file pone.0218954.s005.pdf]

Supporting information, S1 Table. Data survey.
Animal behaviour in a human world: A crowd sourcing study on horses that open door and gate mechanisms
Krueger K, Esch L, Byrne R

Questionaire	sID	pictures / videos attached	single / group stabling	amount of feed	access to pasture	social contact	how often was the behaviour shown	behaviour spreading to other horses	other horses in stable show same behaviour	other horse show same behaviour yes no	nr of diff mechanisms opened	nr of different doors opened	own door	other doors	gate	door on hinges	sliding doors	barred door- or gateway	bolt sideways	bolt upwards / downwards	twist mechanism	opens security chain / rope	handle	carabina	nr of mechasnim positions	goes out of places	stays in the stable	goes into other horses boxes	runs around freely	goes into feed or other rooms	frees other horses	sex	age in years	breed -type
gen.Engl.	1965		2	2	2	2	2	0			2	2	1	1	0	1	0	0	1	0	1	0	0	0	2	1		1				s	1	Warmblood
gen.Engl.	1980		1	2	2	2	4	0			2	2	1	1	0	1	0							0		1		0		0		f	9	Warmblood
gen.Engl.	2003	picture	1	2	2	2	5	1			1	1	1	0	0	1	0									1		0		0		g	8	Haflinger
gen.Engl.	2004		1	1	2	2	3	0			2	2	1	1	0	1	0	0					1	0	2	1		1		1		g	6	Pony
gen.Engl.	2005		1	1	1	1	5	0			1	1	1	0	0	1	0	0	0	1	0	0	0	0	1	1		0		0		g	10	Warmblood
gen.Engl.	2006		1	1	2	2	5	0			1	1	1	0	0	1	0							0		1		0		0		g	8	Warmblood
gen.Engl.	2008		1	1	1	1	4	0			1	1	1	0	0	1	0	0	0	1	0	0	0	0	1	1		0		0		f	6	NA
gen.Engl.	2010		1	1	2	2	5	0			2	1	1	0	0	1	0	0	1	0	0	0	0	1	1	1		0		1		g	8	Pony
gen.Engl.	2011		1	1	2	2	4	0																								f	9	Warmblood
gen.Engl.	2012		1	2	2	2	5	0			3	1	1	0	0	1	0	0	1	1	0		0	0	2	1		0		0		g	13	Warmblood
gen.Engl.	2013		2	2	2	2	2	0			1	1	0	0	1	0	0	1	0	0	0	0	2	0	1	1		1		0		f	4	Warmblood
gen.Engl.	2014		2	2	2	2	4	0			3	1	1	0	0	1	0	0	1	0	1	1	0	0		1		0		0		f	6	Coldblood
gen.Engl.	2016	picture	1	2	2	2	2	0			1	1	1	0	0	1	0	0	0	1	0	0	0	0	1	1		0		0		g	9	Warmblood
gen.Engl.	2018		1	2	1	1	5	0			2	2	1	1	0	1	0	0	1	1	0	0	0	0	2	1		0		0		g	14	Warmblood
gen.Engl.	2019		2	1	2	2	3	0			1	1	0	0	1	0	0	0	0	1	0	0	0	0	1	1		0		0		f	10	Warmblood
gen.Engl.	2021		2	2	2	2	2	0										0														f	6	Warmblood
gen.Engl.	2022		2	2	2	2	4				1	1	0	1	0	1	0	0	0	0	0	0	1	0	1	0		1		1		g	25	Pony
gen.Engl.	2031		1	1	2	1	4	0			2	3	1	1	1	1	0	0	1	1	0	0	1	1	6	1		0		1		g	15	Warmblood
gen.Engl.	2033		1	1	1	1	2	0			3	1	0	0	1	0	0	0	0	1	0	1	0	0	1	1		0		0		g	9	Warmblood
gen.Engl.	2036		2	1	2	2	4	0			3	1	1	0	0	1	0	0	1	1	1	0	0	0		1		0		0		f	12	Warmblood
gen.Engl.	2039	video	1	2	2	2	4	0			1	2	1	1	0	1	0	0	1	0	0	0	0	0	2	1		1		0		g	7	Warmblood
gen.Engl.	2040		1	1	1	1	4	0			3	1	0	0	1	0	0	0	0	1	0	1	0	1	1	1		0		0		f	7	Arabian horse
gen.Engl.	2044		2	1	2	2	5	0				2	1	1	0	1	0	0								0						g	2	Warmblood
gen.Engl.	2062		1	2	2	2	4	0			1	2	1	1	0	1	0	0	1	0	0	0	0	0	1	1		1		1		g	10	Arabian horse
gen.Engl.	2063		1	2	2	2	4	0			1	1	0	0	1	0	0	0	0	0	0	0	0	0		1		0		0		g	5	Arabian horse
gen.Engl.	2064	picture	1	2	2	2	4	1				3	1	1	1	1	0	1								1		1				g	12	Warmblood
gen.Germ.	1947		2	2	2	2	5	1			1	1	0	0	1	0	0	0	0	0	0	0	0	0		1		0		0		f	3	Warmblood
gen.Germ.	1963		1	2	1	1	5	0			2	1	1	0	0	1	0	0	1	1	0	0	0	0	1	1		1		0		f	14	Pony
gen.Germ.	1969		1	1	2	2	5	0			1	1	0	1	0	1	0	0	0	0	0	0	1	0	1	0		0		1		f	13	Warmblood
gen.Germ.	1971		1	1	2	1	4	0			2	1	1	0	0	1	0	0	1	1	0	0	0	0	1	1		0		0		g	6	Warmblood
gen.Germ.	1972		1	2	2	1	2	0			1	1	0	1	0	1	0	0	0	0	0	0	1	0	1	0		0		1		s	30	Pony
gen.Germ.	1977		2	1	2	2	5	0			3	4	1	1	1	1	1	0	1	1	0	0	0	1	3	1		0		0		g	10	Warmblood
gen.Germ.	1982		2	2	2	2	3	0			1	2	1	0	1	1	0	0	1	0	0	0	0	0	2	1		0		1		s	24	Pony
gen.Germ.	1984	video	2	1	2	2		0			3	3	1	1	1	1	0	0	1	1	0	0	1	0	3	1	0	1	0	1	0	g	9	Arabian horse
gen.Germ.	1986		1	1	2	2	5	0			2	3	1	0	1	1	1	0	1	1	0	0	0	0	2	1		0		0		g	7	Warmblood
gen.Germ.	1988	video									1	2	1	0	1	1	0	1	0	0	0	0	2	0	3	1		0		0		g	12	Warmblood
gen.Germ.	1990	video	1	1	1	2	5	0			4	4	1	1	1	1	1	0	1	1	0	0	0	0	9	1		1		1		f	3	Warmblood
gen.Germ.	2000		1		2	2	4	0			2		1			1		0														g	19	Warmblood
gen.Germ.	2069		1	1	1	1	5	0			2	1	0	0	1	0	0	0	1	1	0	0	0	0	2	1		0		0		g	6	Warmblood
gen.Germ.	2123		1				4	2			2	1	1	0	0	1	0	0	1	0	1	0	0	0	1	1		0		0		g		NA
gen.Engl.	2078	picture	2	2	2	2	5	1			3	1	0	0	1	0	0	1	1	0	0	1	0	1	1	1		0		0		g	2	Warmblood
gen.Engl.	2117		1	1	2	2	3	0										0														s		Pony
gen.Engl.	2124		1	1	2	1	3	0										0														s	5	Pony
gen.Engl.	2126		1	2	2	2	4				1	1	1	0	0	1	0	0	0	0	0	0	0	1	1	1		0		0		g	4	Warmblood
gen.Engl.	2127		2	1	2	2	5	0			2	1	0	0	1	0	0	1	0	0	0	1	0	1	1	1		0		0		g	9	Warmblood
gen.Engl.	2146		1	2	2	2	4	0			1	1	1	0	0	1	0	0	1	0	0	0	0	0	1	1		0		0		f	1	Warmblood
gen.Engl.	2148		2	2	2	2	3	0			1	1	0	0	1	0	0	0	0	1	0	0	0	0	1	1		1		0		s	3	Warmblood
gen.Engl.	2150		2	2	2	2	4	0			3	1	1	0	0	1	0	0	1	0	1	1	0	0	1	1		0		0		g	26	Warmblood
gen.Engl.	2152		1	2	2	2	4	0			3	2	1	0	0	1	1	0	1	1	0	0	0	0	3	1		1		1		g	15	Warmblood
gen.Engl.	2153		1	1	2	2	3	0			1	2	1	1	0	1	0	0	0	0	1	0	0	0	1	1		1		0		f	17	Warmblood
gen.Engl.	2163		2	2	2	2	5	1			1	1	0	0	1	0	0	1	1	0	0	0	0	0	1	1		0		0		s	15	Warmblood
gen.Engl.	2168		2	2	2	2		0			1	1	0	1	0	1	0	0	1	0	0	0	0	0	1	0		1		0		g	10	Arabian horse
gen.Germ.	2158		1	1	2	2	5	0			1	1	0	1	0	1	0	0	1	0	0	0	0	0	1	0		0		1		s	10	NA
gen.Germ.	2169		2	2	2	2		0										0														g	7	NA
gen.Germ.	2170		1	1	2	2		1	1	1	3	3	1	1	0	1	1	0	0	1	0	0	1	0	2	1		0		1		f	15	Warmblood
gen.Engl.	2173		2	2	2	2	2	0			2	1	0	0	1	0	0	0	0	1	0	1	0	0	1	1		0		0		g	7	Pony
gen.Germ.	2174		2	2	2	2	2	0			2	1	1	0	0	1	0	0	1	1	0	0	0	0	2	1		0		0		g	16	Warmblood
gen.Germ.	2175		2	2	2	2	5	0			1	1	0	1	0	1	0	0	0	0	0	0	1	0	1	0		0		1		g	7	Warmblood
gen.Germ.	2217	picture	2	2	2	2	5	0			1	2	0	1	0	1	1	0	0	0	0	0	1	0	1	0		0		1		g	5	Arabian horse
gen.Germ.	2233		2	2	2	2	4	1			1	1	0	1	0	1	0	0	1	0	0	0	0	0	1	0		0		1		f	28	Haflinger
gen.Germ.	2238		2	2	2	2	4	0			2	1	0	0	1	0	0	1	0	0	0	0	2	1	2	1		1		1		f	6	Warmblood
gen.Germ.	2251		1	1	1	1	2	0			2	1	1	0	0	1	0	0	0	0	1	0	1	0	2	1		0		0		g	7	Haflinger
gen.Germ.	2297		1	1	2	2	2	1			3	2	1	0	1	1	0	1	1	0	1	0	2	0	3	1		0		0		f	5	Warmblood
gen.Germ.	2404		2	2	2	2	5	0			2	1	0	1	0	1	0	0	1	1	0	0	0	0	2	0		1		0		g	10	Warmblood
gen.Germ.	2405		2	2	2	2	2	0			1	1	0	0	1	0	0	0	0	1	0	0	0	0	1	1		0		0		g	12	Warmblood
gen.Germ.	2470	picture	2	2	2	2	4	0	1	1	3	2	1	0	1	1	0	1	1	0	0	0	2	0	3	1	0	0	1	0	1	g	12	Pony
gen.Germ.	2470a	picture	2	2	2	2	4	1	0	0	1	1	0	0	1	0	0	1	0	0	0	0	2	0	1	1		0	1	0	1	g	10	Pony
gen.Engl.	2265		2	2	2	2	2	0			1	2	1	0	0	1	1	0	0	0	0	1	0	0	1	1		0		1		f	4	Warmblood
gen.Engl.	2439		2	2	2	2	5	0			1	1	0	0	1	0	0	0	0	1	0	1	0	0	1	1		0		0		g	4	Arabian horse
gen.Engl.	2504		2	2	2	2	4	0	0	0	1	1	0	0	1	0	0	1	0	0	0	1	0	0	1	1	1	0	1	1	0	g	8	Arabian horse
gen.Engl.	2506		2	1	2	2	2	0	0	0	1	1	0	0	1	0	0	1	0	0	0	0	0	0	1	1	1	0	1	1	0	f	13	Warmblood
door_gate_Germ.	2182		2	1	1	2	2	1	1	1	2	2	1	0	1	1	0	0	1	1	0	0	0	0	2	1	0	0	1	1	0	g	6	Pony
door_gate_Germ.	2183		2	1	1	2	4	1	1	1	1	1	1	0	0	1	0	0	1	0	0	0	0	0	1	1	0	0	1	1	0	g	15	Haflinger
door_gate_Germ.	2184		2	1	2	2	4	0	0	0	2	2	1	1	0	1	0	0	1	0	1	1	0	1	3	0	0	0	1	1	0	g	14	Coldblood
door_gate_Germ.	2185		1	2	1	2	2	1	1	1	1	2	1	1	0	1	0	0	0	1	0	0	0	0	2	0	0	0	1	1	0	g	10	Warmblood
door_gate_Germ.	2186		1	1	2	2	5	1	1	1	2	2	1	1	0	1	0	0	1	0	0	0	0	1	2	0	0	0	1	0	0	g	20	Warmblood
door_gate_Germ.	2187		1	2	2	2	4				1	1	0	0	1	0	0	1	0	0	0	1	2	0	1	1	0	0	1	0	1	g	5	Warmblood
door_gate_Germ.	2189		1	2	1	2	4	0	0	0	3	2	1	1	0	1	0	0	1	1	0	0	0	0	3	1	0	0	1	0	1	g	7	Haflinger
door_gate_Germ.	2190		1	1	2	1	4	1	1	1	2	2	1	0	1	1	0	0	1	0	0	0	0	0	2	1	0	0	1	0	0	g	5	Haflinger
door_gate_Germ.	2192		1	1	1	1	4	0	0	0	3	3	1	1	0	1	1	0	1	1	0	0	0	0	3	1	0	0	1	0	0	f	11	Warmblood
door_gate_Germ.	2193		2	2	2	2	3	0	1	1	2	2	0	1	1	1	0	0	0	0	0	0	1	0	2	1	0	0	1	0	0	g	4	Pony
door_gate_Germ.	2195		2	2	1	2	4	0	0	0	1	1	1	0	0	1	0	0	0	1	0	0	0	0	1	1	1	0	1	0	0	g	24	Warmblood
door_gate_Germ.	2196		2	2	1	2	2	0	1	1	1	2	1	1	0	1	0	0	1	0	0	0	0	0	2	1	1	0	1	0	0	f	2	Warmblood
door_gate_Germ.	2197		1	1	1	1	3	0	0	0	2	2	1	0	0	1	1	0	0	0	1	1	0	0	1	1	1	0	1	0	0	s	24	Warmblood
door_gate_Germ.	2198		2	1	1	2	4	1	1	1	2	1	1	0	0	1	0	0	1	0	0	0	1	0	2	1	0	0	1	0	0	g	15	Haflinger
door_gate_Germ.	2199		2	1	2	2			1	1	1	1	1	0	0	1	0	0	1	0	0	1	0	0	1							g	8	Warmblood
door_gate_Germ.	2201		1	1	1	1	2	0	0	0	3	3	1	1	1	1	0	0	1	0	1	0	0	0	3	1	1	1	1	0	0	g	5	Haflinger
door_gate_Germ.	2202	picture	2		1	2	4	1	1	1	1	2	1	1	0	1	0	0	1	0	1	0	0	0	2	1	1	0	1	0	1	f	4	Warmblood
door_gate_Germ.	2203		1	2	2	2	2	0	0	0	1	1	1	0	0	1	0	0	1	0	1	0	0	0	1	1	1	0	0	1	0	g	17	Coldblood
door_gate_Germ.	2210		2	2	2	2	5				3	2	0	1	1	1	0	0	1	1	0	0	1	0	3	0	1	0	1	0	0	g	18	Pony
door_gate_Germ.	2211		2	2	2	2	5	1	1	1	2	1	1	0	0	1	0	0	1	0	0	0	0	1	2	1	0	0	1	0	0	g	13	Arabian horse
door_gate_Germ.	2212		1	2	2	1	4	0	0	0	2	2	1	1	0	1	0	0	1	0	0	0	0	1	3	1	0	1	1	0	1	g	3	Warmblood
door_gate_Germ.	2213		2	2	2	2	4	0			2	1	1	0	0	1	0	0	0	0	1	0	1	0	2	1	0	0	1	0	1	g	20	Warmblood
door_gate_Germ.	2214		1	1	1	1	1	0	0	0	1	1	1	0	0	1	0	0	0	1	0	0	0	0	1	1	0	0	1	0	0	g	11	Warmblood
door_gate_Germ.	2215		2	2	1	2	3	0	0	0	4	3	1	1	1	1	0	1	1	1	1	0	3		4	1	0	1	1	0	1	g	15	Warmblood
door_gate_Germ.	2216		2	1	2	2	2	0	1	1	4	3	1	1	1	1	0	0	1	1	0	1	0	0	4	1	0	1	1	1	1	g	14	Warmblood
door_gate_Germ.	2218		2	1	1	2	5	1	2	1	1	1	1	0	0	1	0	0	0	1	0	1	0	0	1	1	1	0	1	0	0	f	9	Warmblood
door_gate_Germ.	2220		2	2	2	2	4	0	0	0	2	1	0	0	1	0	0	1	0	1	0	0	2	0	2	1	0	0	1	0	0	f	6	Warmblood
door_gate_Germ.	2221		2	2	2	2	4	0	1	1	2	1	0	0	1	0	0	0	0	1	0	0	0	0	2	1	0	0	1	0	0	g	17	Warmblood
door_gate_Germ.	2222		2	1	2	2	3	0	1	1	2	3	1	1	0	1	1	0	0	1	0	0	0	0	2	1	0	1	1	1	1	g	10	Pony
door_gate_Germ.	2223	picture	1	1	1	1	4	2	2	1	1	2	1	1	0	1	0	0	1	0	0	0	0	0	2	1	1	0	1	0	0	f	10	Warmblood
door_gate_Germ.	2224		1	2	1	1	4	0	0	0	2	3	1	1	0	1	1	0	0	0	1	0	0	0	2	1	1	0	1	0	0	f	10	Warmblood
door_gate_Germ.	2227		1	1	1	1	3	0	0	0	1	1	1	0	0	1	0	0	1	0	0	0	0	0	1	1	0		1	0	0	g	6	Warmblood
door_gate_Germ.	2228		1	2	1	1	3	1	0	0	3	3	1	1	1	1	0	0	1	1	0	1	0	0	1	1		0		0		s	15	Coldblood
door_gate_Germ.	2229		1	1	1	1	4	1	1	1	1	1	0	1	0	1	0	0	0	0	0	0	1	0	1	0		1		0		g	10	Warmblood
door_gate_Germ.	2230		2	1	2	2	5	0	0	0	2	3	1	1	0	1	1	0	1	0	0	0	0	0	1	0		0		1		s	10	Haflinger
door_gate_Germ.	2232		2	1	2	2	4	0	0	0	4	2	1	1	0	1	0	0	1	1	0	1	0	1								g	7	Haflinger
door_gate_Germ.	2234		2	2	1	2	2	0	0	0	2	2	1	1	0	1	0	0	1	1	0	0	0	0	2	1		0		1		f	15	Warmblood
door_gate_Germ.	2235		1	2	2	1		0	1	1	3	1	1	0	0	1	0	0	1	0	0	1	0	1	1	1		0		0		g	7	Warmblood
door_gate_Germ.	2236		1	1	2	1		0	0	0	4	2	1	1	0	1	0	0	1	1	1	1	0	0	2	1		0		0		g	16	Warmblood
door_gate_Germ.	2237		1	1	2	1	5	0	0	0	3	1	1	0	0	1	0	0	0	1	0	1	0	1	1	0		0		1		g	7	Warmblood
door_gate_Germ.	2239		1	2	2	1	4	0	0	0	2	1	1	0	0	1	0	0	1	0	1	0	0	0	1	0		0		1		g	5	Warmblood
door_gate_Germ.	2240	picture	2	1		2	4	0	0	0	1	2	1	0	1	1	0	0	0	1	0	1	0	0	1	0		0		1		f	28	Pony
door_gate_Germ.	2241		1	1	2	1	4	0	0	0	2	2	1	0	0	1	1	0	0	1	0	1	0	0	2	1		1		1		f	6	Warmblood
door_gate_Germ.	2243		2	2	2	2	5	0	1	1	5	4	1	1	1	1	1	0	1	1	0	1	1	1	2	1		0		0		g	7	Warmblood
door_gate_Germ.	2244		1	1	2	1	4	0	0	0	4	1	1	0	0	1	0	0	1	1	0	1	0	1	3	1		0		0		f	5	Warmblood
door_gate_Germ.	2246		2	1	2	2	4	0	0	0	1	1	1	0	0	1	0	0	1	0	0	0	0	0	2	0		1		0		g	10	Haflinger
door_gate_Germ.	2248		1	1	1	1	4	1	2	1	3	4	1	1	1	1	1	1	1	0	0	0	2	1	1	1		0		0		g	12	Warmblood
door_gate_Germ.	2249		2	2	2	2	2	1	0	0	2	1	0	0	0	0	0	1	1	0	0	0	1	1	3	1	0	0	1	0	1	g	12	Warmblood
door_gate_Germ.	2250		2	2	2	2	2	0	2	1	1	1	0	0	1	0	0	0	0	1	0	1	0	0	1	1		0	1	0	1	g	10	Coldblood
door_gate_Germ.	2252	video	2	1	2	2	5	0	0	0	2	1	1	0	0	1	0	0	0	1	0	0	0	0	1	1		0		1		f	4	Warmblood
door_gate_Germ.	2253		2	1	1	2	2	1	1	1	2	2	1	0	1	1	0	0	1	0	0	0	0	0	1	1		0		0		g	4	NA
door_gate_Germ.	2254		2	1	2	2	2	0	1	1	1	1	1	0	0	1	0	0	0	1	0	0	0	0	1	1	1	0	1	1	0	g	8	Coldblood
door_gate_Germ.	2255		1	1	1	1	2	0	0	0	1	1	1	0	0	1	0	0	1	0	1	0	0	0	1	1	1	0	1	1	0	f	13	Warmblood
door_gate_Germ.	2256		2	2	2	2	4	0	0	0	1	1	0	0	1	0	0	0	0	1	0	1	0	0	2	1	0	0	1	1	0	g	6	Coldblood
door_gate_Germ.	2257		2	2	2	2		0	0	0	1	1	1	0	0	1	0	0	1	0	0	0	0	0	1	1	0	0	1	0	0	g	15	Warmblood
door_gate_Germ.	2258		2	1	2	2	4	2	1	1	2	2	1	1	0	1	0	0	1	0	0	0	1	0	3	1	0	0	1	0	0	g	14	Coldblood
door_gate_Germ.	2262		1	1	1	1	1	0	1	1	1	1	1	0	0	1	0	0	1	0	0	0	0	0	2	0	0	0	1	1	0	g	10	Arabian horse
door_gate_Germ.	2264		2	1	2	2	5	0	0	0	1	1	1	0	0	1	0	0	1	0	0	1	0	0	2	0	0	0	1	0	0	g	20	Arabian horse
door_gate_Germ.	2276		2	1	1	2	3	0	1	1	2	2	1	1	0	1	0	0	1	1	0	0	0	1	1	1	0	0	1	0	1	g	5	Haflinger
door_gate_Germ.	2280		2	2	2	2	4	0	0	0	2	2	1	0	1	1	0	0	1	0	0	0	2	0	3	1	0	0	1	0	1	g	7	Warmblood
door_gate_Germ.	2281		2	1	2	2	4	1	1	1	2	3	1	0	1	1	1	1	0	0	0	0	2	0	2	1	0	0	1	0	0	g	5	Arabian horse
door_gate_Germ.	2284		1	1	2	1	2	0	0	0	1	1	1	0	0	1	0	0	1	0	0	1	0	0	3	1	0	0	1	0	0	f	11	Warmblood
door_gate_Germ.	2285		1	1	2	1	2	1	1	1	1	1	1	0	0	1	0	0	1	0	0	1	0	0	2	1	0	0	1	0	0	g	4	Haflinger
door_gate_Germ.	2286		2	1	2	2	5	0	0	0	1	1	0	0	1	0	0	1	1	0	0	0	0	0	1	1	1	0	1	0	0	g	24	Haflinger
door_gate_Germ.	2287		2	1	2	2	4	0	0	0	3	2	1	0	1	1	0	0	1	1	0	0	1	0	2	1	1	0	1	0	0	f	2	Pony
door_gate_Germ.	2288	picture	1	1	1	1	1	0	0	0	1	1	0	0	1	0	0	0	0	1	0	0	0	0	1	1	1	0	1	0	0	s	24	Haflinger
door_gate_Germ.	2289		1	2		1	4	0	0	0								0							2	1	0	0	1	0	0	g	15	Coldblood
door_gate_Germ.	2290		1	2	1	1	4	0	0	0	2	2	1	0	1	1	0	0	0	1	0	0	0	1	1							g	8	Warmblood
door_gate_Germ.	2291		2	1	2	2	4	0	0	0	3	2	1	1	0	1	0	0	1	0	1	0	1	1	3	1	1	1	1	0	0	g	5	Haflinger
door_gate_Germ.	2292		2	2	2	2	2	1	0	0	1	1	1	0	0	1	0	0	0	0	1	0	0	0	2	1	1	0	1	0	1	f	4	Warmblood
door_gate_Germ.	2293		1	1	1	1	3	0	0	0	1	1	1	0	0	1	0	0	1	0	0	0	0	0	1	1	1	0	0	1	0	g	17	Warmblood
door_gate_Germ.	2294		1	1	1	1	2	0	0	0	2	1	1	0	0	1	0	0	1	0	0	0	0	1	3	0	1	0	1	0	0	g	18	NA
door_gate_Germ.	2295		1	1	1	1	4	0	1	1	2	2	1	0	1	1	0	1	1	0	0	0	2	0	2	1	0	0	1	0	0	g	13	Warmblood
door_gate_Germ.	2295		1	1	1	1	4	1	0	0	1	2	1	1	0	1	0	0	1	0	0	0	0	0	3	1	0	1	1	0	1	g	3	Warmblood
door_gate_Germ.	2296		2	1	1	2	2	0	0	0	1	2	1	0	0	1	1	0	0	1	0	0	0	0	2	1	0	0	1	0	0	g	20	Warmblood
door_gate_Germ.	2298		2	1	2	2	5	0	0	0	2	2	1	1	0	1	0	0	1	1	0	1	0	0	1	1	0	0	1	0	0	g	11	Warmblood
door_gate_Germ.	2300	picture	1	1	2	1	4		2	1	4	3	1	1	1	1	0	1	1	1	0	1	2	0	4	1	0	1	1	0	1	g	15	Arabian horse
door_gate_Germ.	2301		2	1	1	2	4	0	0	0	1	1	1	0	0	1	0	0	1	0	0	0	0	0	4	1	0	1	1	1	1	g	14	Arabian horse
door_gate_Germ.	2302		1	2	1	1	3	0	1	1	1	1	0	0	1	0	0	0	0	1	0	0	0	0	1	1	1	0	1	0	0	f	9	Warmblood
door_gate_Germ.	2304		1	1	2	1	2	0	2	1	1	1	0	0	1	0	0	1	1	0	0	1	0	0	2	1	0	0	1	0	0	f	6	Warmblood
door_gate_Germ.	2305		1	1	1	1	2	0	1	1	1	1	1	0	0	1	0	0	0	0	0	0	0	1	2	1	0	0	1	0	0	g	17	Coldblood
door_gate_Germ.	2306		2	1	2	2	2	0	0	0	1	1	1	0	0	1	0	0	1	0	0	0	0	0	2	1	0	1	1	1	1	g	10	Coldblood
door_gate_Germ.	2307		2	2	2	2	2	0	0	0	2	1	0	0	1	0	0	1	1	0	0	0	2	0	2	1	1	0	1	0	0	f	10	Pony
door_gate_Germ.	2308		1	2	2	1	3	0	1	1	2	2	1	0	0	1	1	0	0	1	0	0	0	0	2	1	1	0	1	0	0	f	10	Pony
door_gate_Germ.	2309		1	2		1	4	0	0	0	5	2	1	1	0	1	0	0	1	1	1	1	1	0	1	1	0		1	0	0	g	6	Warmblood
door_gate_Germ.	2310		2	2	2	2	5	0	0	0	2	2	1	1	0	1	0	0	0	1	0	0	0	0	3	1	1	1	1	0	0	g	4	Warmblood
door_gate_Germ.	2311	picture	1	1	2	1	2	0	1	1	2	1	1	0	0	1	0	0	1	1	0	0	0	0	1						0	f	9	Warmblood
door_gate_Germ.	2314		2	2	2	1	2	0	0	0	1	1	0	0	1	0	0	0	0	1	0	0	0	0	2	1	0	1	1	1	0	f	10	Pony
door_gate_Germ.	2316		2	1	2	2	4	0	0	0	1	1	1	0	0	1	0	0	1	0	0	0	0	0	3	1	0	1	1	1	0	f	18	Coldblood
door_gate_Germ.	2318		1	1	1	1	2	0	0	0	1	0	0	0	0	0	0	1	0	0	0	0	2	0	2	1	1	0	0	0	0	g	1	Warmblood
door_gate_Germ.	2319		1	2	1	1	5	0	0	0	1	1	1	0	0	1	0	0	1	0	0	0	0	0	1	1	0	0	1	0	0	g	10	Warmblood
door_gate_Germ.	2320		2	1	1	2	4	0	0	0	2	1	0	0	1	0	0	0	0	1	0	1	0	0	2	1	0	0	1	0	0	g	14	NA
door_gate_Germ.	2322		2	1	2	2	2	0	0	0	1	1	0	0	1	0	0	0	0	1	0	0	0	0	1	1	0	0	1	0	1	g	17	Warmblood
door_gate_Germ.	2323		1	1	1	1	3	0	0	0	1	1	0	1	0	1	0	0	1	0	0	0	0	0	1	1	0	0	1	0	0	g	8	Warmblood
door_gate_Germ.	2324	picture	1		1	1	3	0	0	0	1	1	1	0	0	1	0	0	1	0	0	0	0	0	1	1	0	0	1	0	0	g	7	Thorough bred
door_gate_Germ.	2329		2	1	1	2	5	0	0	0	2	1	1	0	0	1	0	0	1	0	0	0	0	1	1	1	1	0	1	0	0	f	19	Haflinger
door_gate_Germ.	2332		1	1	2	1	2	0			1	2	1	1	0	1	0	0	1	0	0	0	0	0	4	1	0	1	1	1	1	g	5	NA
door_gate_Germ.	2333		2	1	2	2		1	0	0	1	2	1	1	0	1	0	0	1	0	0	0	0	0	1	1	1	1	1	1	1	f	21	Pony
door_gate_Germ.	2334		1	2	1	1	2	0	0	0	3	3	1	1	0	1	1	0	1	1	0	0	0	0	1	1	0	0	1	0	0	g	6	Warmblood
door_gate_Germ.	2335		1	1	2	1	4	0	0	0	1	1	1	0	0	1	0	0	0	0	1	0	0	0	4	1	0	0	1	0	0	g	9	Coldblood
door_gate_Germ.	2336		2	2	2	2	2		0	0	4	3	1	1	1	1	0	1	1	1	0	0	2	0	2	1	0	0	1	0	0	f	17	Warmblood
door_gate_Germ.	2337		1	2	1	1	1	0	2	1	1	1	1	0	0	1	0	0	1	0	0	0	0	0	1	1	0	1	1	0	1	g	2	Warmblood
door_gate_Germ.	2338	picture	1	2	1	1	2	0	0	0	1	1	1	0	0	1	0	0	0	1	0	0	0	0	2	1	1	0	1	0	0	g	5	Warmblood
door_gate_Germ.	2341	video	2	2	2	2	5	0	0	0	1	2	1	1	0	1	0	0	1	0	0	0	0	0	2	1	0	1	1	1	1	g	8	Warmblood
door_gate_Germ.	2343		1	2	1	1	2	0	0	0	1	1	1	0	0	1	0	0	1	0	0	0	0	0	1	1	0	0	1	0	0	g	9	Warmblood
door_gate_Germ.	2340		2	2	2	2	4	0	0	0	2	2	1	1	0	1	0	0	1	0	0	0	1	0	1	0	1	0	0	0	0	g	7	Warmblood
door_gate_Germ.	2344	picture	1	2	1	1	2	0	0	0	1	1	1	0	0	1	0	0	1	0	0	0	0	0	1	1	0	0	1	0	0	s	3	Warmblood
door_gate_Germ.	2345		2	1	2	2	5	1	0	0	2	2	1	0	0	1	1	0	0	1	0	0	0	0	1	1	0	0	1	0	0	g	5	Warmblood
door_gate_Germ.	2346		2	2	2	2	4	0	0	0	2	3	1	1	1	1	0	0	1	1	0	0	0	0	2	1	1	0	0	0	0	g	5	Warmblood
door_gate_Germ.	2348	picture	2	2	2	2		1	1	1	1	1	1	0	0	1	0	0	1	0	0	0	0	0	2	1	1	0	1	0	0	g	14	Warmblood
door_gate_Germ.	2346		1	1	1	1	5	1	1	1	1	1	1	0	0	1	0	0	1	0	0	0	0	0	1	1	0	0	1	1	0	f	4	Warmblood
door_gate_Germ.	2350		1	1	1	1	2	0	0	0	3	1	1	0	0	1	0	0	1	1	0	0	0	1	4	1	0		1	0	0	g	6	NA
door_gate_Germ.	2352		1	2	1	1	5	0	2	1	1	2	1	0	0	1	1	0	0	0	0	0	0	0	2	1	0	1	1	1	1	g	12	Warmblood
door_gate_Germ.	2354	picture	1	1	1	1	4	1	1	1	1	1	1	0	0	1	0	0	1	0	0	0	0	0	2	1	0	1	1	1	1	g	18	Pony
door_gate_Germ.	2356		2	1	1	2	2	0	0	0	4	3	1	1	1	1	0	0	1	1	0	0	1	0	1	1	0	1	1	0	0	g	11	Pony
door_gate_Germ.	2357		2	1	2	2	2	0	0	0	1	1	1	0	0	1	0	0	1	0	0	0	0	0	1	1	0	0	1	0	0	g	6	Warmblood
door_gate_Germ.	2358		1	1	1	1	3	0	0	0	2	2	1	0	0	1	1	0	1	1	0	0	0	0	1	1	0	1	0	0	1	g	10	Coldblood
door_gate_Germ.	2359		1	1	1	1	1	0	0	0	1	1	0	0	0	0	0	0	0	1	0	0	0	0	3	1	0	0	1	0	0	g	10	Pony
door_gate_Germ.	2361		2	2	2	2	3	0	0	0	1	2	1	1	0	1	0	0	1	0	0	0	0	0	1	1			1	1	0	f	4	Coldblood
door_gate_Germ.	2363		1	1	1	1	4	0	0	0	1	1	1	0	0	1	0	0	1	0	0	0	0	1	2	1	1	0	1	0	1	g	8	Warmblood
door_gate_Germ.	2364		2	2	2	2	5	0	0	0	1	2	1	1	0	1	0	0	0	1	0	0	0	0	2	1			1	0	1	g	12	Warmblood
door_gate_Germ.	2365		2	1	1	2	4	2	0	0	1	1	1	0	0	1	0	0	0	1	0	1	0	0	4	1	0	0	1	0	0	g	28	Arabian horse
door_gate_Germ.	2366		1	2	1	2	2	0	0	0	1	1	1	0	0	1	0	0	1	0	0	0	0	0	1	1	0	0	1	0	0	g	12	Warmblood
door_gate_Germ.	2367		1		1	1	2	0	0	0	2	2	1	1	0	1	0	0	1	1	0	1	0	0	1	0	1	0	0	0	0	g	16	NA
door_gate_Germ.	2370		2	2	2	2	2	0	0	0	1	1	0	1	0	1	0	0	0	0	0	0	1	0	1	1	0	0	1	0	1	g	10	Pony
door_gate_Germ.	2371	picture	2	1	2	2		0	0	0	2	2	1	0	0	1	1	0	1	0	0	0	0	0	2	1	1	1	1	0	1	f	5	Haflinger
door_gate_Germ.	2373		2	2	2	2	2				2	1	1	0	0	1	0	0	1	1	0	0	0	0	2	1	1	1	1	0	1	g	11	Coldblood
door_gate_Germ.	2374		1	1	1	1	2	2	0	0	2	1	1	0	0	1	0	0	1	1	0	0	0	0	1	1	0	0	1	0	0	f	5	Warmblood
door_gate_Germ.	2376		2	2	2	2	4	1	1	1	2	2	1	0	0	1	1	0	1	1	0	1	0	0	2	1	0	0	1	0	0	g	4	Warmblood
door_gate_Germ.	2379	picture	2	1	2	2	2				2	3	1	1	1	1	0	0	1	1	0	0	0	1	4	1	0	0	1	0	0	f	5	Pony
door_gate_Germ.	2381		2	1	2	2	3	0	0	0	1	1	0	0	1	0	0	1	0	0	0	0	2	0	1	1	1	0	1	0	0	g	15	Pony
door_gate_Germ.	2382		2	1	2	2	2		2	1	1	1	0	0	0	0	0	1	0	0	0	0	0	1	1	1	1	0	0	0	0	g	2	Coldblood
door_gate_Germ.	2383		2	1	2	2	2	0	0	0	1	1	1	0	0	1	0	0	1	1	0	0	0	0	1	1	0	0	1	0	0	f	10	Pony
door_gate_Germ.	2384	picture	1	1	1	1	4	0	1	1	2	1	1	0	0	1	0	0	1	1	0	0	0	0	1	1	0	0	1	0	0	g	6	Warmblood
door_gate_Germ.	2385		1	1	2	1	4	0	0	0	2	1	1	0	0	1	0	0	1	1	0	1	0	0	1	1	1	0	1	0	0	g	8	Haflinger
door_gate_Germ.	2386		2	1	2	2	2	0	1	1	1	1	0	0	0	0	0	1	1	0	0	0	0	0	2	1	0	0	1	1	0	g	15	Thorough bred
door_gate_Germ.	2387	picture	2	2	2	2	2	2	2	1	1	1	0	1	0	1	0	0	1	0	0	0	0	0	2	1	0	0	1	0	0	g	10	Coldblood
door_gate_Germ.	2388		2	2	2	2	4	1	1	1	2	2	1	1	0	1	0	0	1	0	1	0	0	0	4	1	0	1	1	1		f	10	Arabian horse
door_gate_Germ.	2389	picture	2	2	2	2	4	0	0	0	4	3	1	0	1	1	1	1	1	0	0	0	2	0	4	1	0	0	1	0	0	g	7	Warmblood
door_gate_Germ.	2390		1	2	1	1	3	0	0	0	3	3	1	1	0	1	1	0	1	0	0	0	1	0	4	1	0	0	1	1	0	g	9	Pony
door_gate_Germ.	2391		2	2	2	2	5	0			1	1	0	0	1	0	0	1								1	1	0	0	1	0	g	13	Coldblood
door_gate_Germ.	2393		1	1	1	1	4	0	0	0	2	1	1	0	0	1	0	0	1	1	0	1	0	0	1	1	0	0	1	0	0	g	3	Warmblood
door_gate_Germ.	2396		1	1	1	1	2	0	0	0	3	3	1	0	1	1	1	0	1	1	0	0	0	0	3	1	0	0	1	0	0	g	8	Haflinger
door_gate_Germ.	2397		1	1	2	2	4	0	0	0	1	1	1	0	0	1	0	0	1	0	0	0	0	0	1	1	1	1	1	1	1	f	5	Pony
door_gate_Germ.	2399		2	2	2	2	5	1	1	1	1	1	1	0	0	1	0	0	1	0	0	0	0	0	1	1	0	0	1	0	1	f	9	Coldblood
door_gate_Germ.	2400		2	1	2	2	5	0	1	1	2	2	1	1	0	1	0	0	1	0	0	1	0	0	2	1	0	0	1	0	0	g	3	NA
door_gate_Germ.	2401		1	2	1	1	5	0	2	1	2	1	1	0	0	1	0	0	1	1	0	0	0	0	2	1	0	1	1	1	1	s	5	Warmblood
door_gate_Germ.	2402		2	1	2	2	5	1	1	1	2	2	1	0	1	1	0	0	1	1	0	1	0	0	3	1	0	1	1	1	1	g	10	Warmblood
door_gate_Germ.	2406		2	1	1	2	5	2	2	1	3	3	1	1	1	1	0	0	1	1	0	1	0	0	3	1	0	1	1	1	1	g	14	Warmblood
door_gate_Germ.	2407	pictures	1	1	1	1	5	0	1	1	2	1	1	0	0	1	0	0	0	1	0	0	0	0	1	0	1	0	0	0	0	f	6	Warmblood
door_gate_Germ.	2408		1	1	1	1	2	0	0	0	1	1	1	0	0	1	0	0	0	1	0	0	0	0	1	0	1	0	0	0	0	f	7	Arabian horse
door_gate_Germ.	2409		1	2	2	2	2	0	0	0	1	1	0	0	1	0	0	0	0	1	0	0	0	0	1	0	1	0	0	0	0	g	7	Warmblood
door_gate_Germ.	2410		1	1	1	1	3	0	0	0	1	1	1	0	0	1	0	0	1	0	0	0	0	1	1	1	0	0	1	0	0	f	10	Warmblood
door_gate_Germ.	2411		2	2	2	2	2	0	0	0	1	1	0	0	1	0	0	0	0	1	0	0	0	0	1	1	0	0	1	0	0	g	5	Arabian horse
door_gate_Germ.	2412		1	1	1	1	2	1	1	1	1	1	0	0	1	0	0	1	0	0	0	0	2	0	1	1	0	0	1	0	0	g	19	Warmblood
door_gate_Germ.	2412a		1	1	1	1	2	1	1	1	1	1	0	0	1	0	0	1	0	0	0	0	2	0	1	0	1	0	0	0	0	NA		NA
door_gate_Germ.	2413		1	2	2	1	2	0	1	1	3	3	1	1	1	1	0	0	1	1	0	1	0	0	4	1	1	0	1	0	0	g	4	NA
door_gate_Germ.	2418		2	2	2	2	2	0	0	0	2	2	1	0	0	1	1	0	0	1	0	0	0	0	2	0	1	0	0	0	0	g	12	Warmblood
door_gate_Germ.	2419		1	1	1	1	2	0	0	0	2	2	1	0	0	1	1	0	0	1	0	0	0	0	2	1	0	0	1	0	0	f	8	Warmblood
door_gate_Germ.	2420		2	2	1	2		0	0	0	1	1	1	0	0	1	0	0	1	0	0	1	0	0	1	0	1	0	1	0	0	g	11	Warmblood
door_gate_Germ.	2421	picture	2	1	2	2	4	0	0	0	2	2	1	1	0	1	0	0	1	1	0	0	0	0	3	1	0	1	1	1	1	g	22	Haflinger
door_gate_Germ.	2422		2	2	2	2	2	0	0	0	1	1	1	0	0	1	0	0	1	0	0	1	0	0	1	1	0	0	1	0	0	g	7	Pony
door_gate_Germ.	2422a		2	2	2	2	2	0	1	1	1	1	1	0	0	1	0	0	1	0	0	1	0	0	1	1	0	0	1	0	0	g		Pony
door_gate_Germ.	2425		1	1	1	1	3				3	1	0	0	1	0	0	1	1	0	0	1	2	0	3	0	1	0	0	0	0	g	3	Warmblood
door_gate_Germ.	2426		2	2	1	2	4	0	0	0	1	1	0	0	1	0	0	1	0	0	0	0	2	0	1	1	0	0	1	0	0	s	5	Warmblood
door_gate_Germ.	2427		1	2	1	1	2	2	2	1	3	2	1	0	1	1	0	0	1	0	1	1	0	0	5	1	0	1	1	1	1	g	9	Warmblood
door_gate_Germ.	2432		2	1	2	2	2	0	0	0	3	3	1	1	0	1	1	0	1	1	0	1	0	0	3	1	0	0	1	0	0	g	13	Pony
door_gate_Germ.	2433		2	2	2	2	2	0	2	1	2	1	1	0	0	1	0	0	1	1	0	0	0	0	2	1	1	0	1	0	0	g	10	Warmblood
door_gate_Germ.	2434		2	1	2	2	5	0	0	0	1	2	0	0	1	0	1	1	0	0	0	0	0	0	1	1	1	0	0	1	0	g	13	Warmblood
door_gate_Germ.	2435		1	2	1	1	2	1	0	0	1	2	1	1	0	1	0	0	0	1	0	1	0	0	2	0	1	0	0	0	1	g	2	Warmblood
door_gate_Germ.	2436		2	2	1	2	4	0	0	0	1	2	1	1	0	1	0	0	1	0	0	0	0	0	2	1	0	0	1	0	1	f	11	Haflinger
door_gate_Germ.	2437		1	1	1	1	5	0	0	0	2	2	1	1	0	1	0	0	0	0	1	0	1	0	3	1	0	1	0	1	1	f	5	Haflinger
door_gate_Germ.	2443		1	1	1	1	2	0	0	0	1	1	1	0	0	1	0	0	1	0	0	1	0	0	3	1	0	0	1	0	0	s	20	Warmblood
door_gate_Germ.	2446		1	1	1	1	4	0	0	0	1	2	1	0	0	1	1	0	0	0	0	0	0	0	1	1	1	0	0	1	0	f	26	Warmblood
door_gate_Germ.	2448		2	1	1	2	2	0	0	0	1	1	1	0	0	1	0	0	1	0	0	0	0	0	1	1	1	0	0	0	0	g	5	Pony
door_gate_Germ.	2449		2	1	1	2	2		0	0	2	2	1	0	1	1	0	0	1	1	0	0	0	0	2	1	0	0	1	0	1	g	13	Pony
door_gate_Germ.	2451		2	1	2	2	4	0	0	0	4	2	1	1	0	1	0	0	1	1	1	1	1	0	4	1	1	1	1	1	1	f	5	Warmblood
door_gate_Germ.	2454		2	2	2	2	4	0	0	0	1	1	0	0	1	0	0	1	0	0	0	0	2	0	1	1	0	0	1	0	0	g	3	Arabian horse
door_gate_Germ.	2456	picture	1	2	2	2	4	1	2	1	5	3	1	1	1	1	0	0	1	1	1	1	0	1	5	1	0	1	1	1	1	f	3	Quarter Horse
door_gate_Germ.	2509	picture	2	1	2	2	3	1	0	0	1	1	1	0	0	1	0	0	0	1	0	0	0	0	1	1			1			f	0,5	Arabian horse
door_gate_Engl.	2245		2	2	2	2	4	0	0	0	3	3	1	1	1	1	0	0	1	1	0	1	1	0	3	1	0	0	1	1	0	g	8	Pony
door_gate_Engl.	2259		1	2	1	1	3	0	0	0	3	2	1	0	1	1	0	0	1	0	0	1	0	1	3	1	1	0	0	0	0	g	5	Warmblood
door_gate_Engl.	2261		1	1	2	1	5	2	1	1	3	3	1	1	0	1	1	0	1	1	1	0	0	0	3	1	0	1	1	1	1	f	2	Arabian horse
door_gate_Engl.	2263		2	2	2	2	5	1	0	0	1	1	0	0	1	0	0	1	0	0	0	0	2	0	1	1	1	0	1	1	0	f	7	Warmblood
door_gate_Engl.	2266		1	2	1	1	3	0	0	0	3	4	1	1	1	1	1	0	0	1	1	0	0	0	3	1	0	0	1	1	0	g	7	Warmblood
door_gate_Engl.	2267	picture	2	1	2	2	3	1	0	0	2	2	1	1	0	1	0	0	1	1	0	0	0	0	2	0	1	0	0	1	0	f	20	Warmblood
door_gate_Engl.	2269		1	2	1	1	5	0	0	0	3	3	1	1	1	1	0	0	1	1	0	1	0	0	3	1	0	1	1	1	1	f	5	Warmblood
door_gate_Engl.	2270		1	2	1	1	3	0	2	1	2	2	1	0	1	1	0	0	1	1	0	0	0	0	2	1	1	0	1	0	0	g	10	Warmblood
door_gate_Engl.	2271	picture	1	1	1	1	5	1	0	0	3	2	1	0	1	1	0	0	1	1	1	0	0	0	3	1	0	0	1	0	1	g	10	Warmblood
door_gate_Engl.	2273		1	2	2	1	4	0	0	0	2	3	1	1	1	1	0	0	1	1	1	0	0	0	3	1	0	0	1	0	1	g	9	Warmblood
door_gate_Engl.	2274		1	1	1	1		0	0	0	2	3	1	1	0	1	1	0	0	1	0	0	0	0	2	1	1	0	1	1	0	f	7	Arabian horse
door_gate_Engl.	2277	video	2	1	2	2	2	0	0	0	1	1	0	0	1	0	0	0	0	1	0	0	0	0	1	1	1	0	0	0	0	f	4	Warmblood
door_gate_Engl.	2278		2	1	2	2	4	0	0	0	3	2	1	1	0	1	0	0	1	0	1	1	0	0	1	0	1	0	1	0	1	f	4	Warmblood
door_gate_Engl.	2317		2	1	2	2	3	0	0	0	2	2	1	0	1	1	0	0	1	0	1	1	0	0	2	1	0	1	1	1	1	g	7	Coldblood
door_gate_Engl.	2321		1	2	2	1	4	0	0	0	1	1	1	0	0	1	0	0	1	0	0	0	0	0	1	1	0	0	1	0	0	NA		NA
door_gate_Engl.	2330		1	2	1	1	3	0	0	0	2	3	1	0	1	1	1	0	0	1	0	1	0	0	2	1	0	1	1	1	1	g	5	Warmblood
door_gate_Engl.	2330a		1	2	1	1	3	0	0	0	1	1	1	0	0	1	0	0	0	0	1	0	0	0	1	1						NA		Pony
door_gate_Engl.	2339		1	1	1	1	3	0	0	0	1	1	1	0	0	1	0	0	1	0	0	0	0	0	1	1	0	0	1	0	0	f	25	Pony
door_gate_Engl.	2347		1	2	2	1	5	0	0	0	2	2	1	1	0	1	0	0	1	1	0	0	0	0	2	1	0	0	1	0	1	g	10	Haflinger
door_gate_Engl.	2351	picture	1	2	1	1	5		1	1	1	1	1	0	0	1	0	0	1	0	0	0	0	0	1	1	0	0	1	0	0	f	20	Warmblood
door_gate_Engl.	2353		1	2	1	1	4	1	0	0	4	2	1	1	0	1	0	0	1	1	1	0	0	1	3	1	0	1	1	1	1	g	28	Haflinger
door_gate_Engl.	2355		1	2	1	1	2	0	0	0	3	1	1	0	0	1	0	0	1	1	1	0	0	0	1	1	0	0	1	0	0	f	7	Thorough bred
door_gate_Engl.	2360		1	1	1	1		0	0	0	2	2	1	0	1	1	0	0	1	1	0	0	0	0	2	1	0	0	1	1	0	g	13	Warmblood
door_gate_Engl.	2362		1	2	1	1	2	0	0	0	2	1	1	0	0	1	0	0	1	0	0	1	0	0	1	1	0	0	1	0	0	g	10	Warmblood
door_gate_Engl.	2369		2	1	1	2	5	0	0	0	1	1	0	0	1	0	0	1	0	0	0	0	2	0	1	1	0	0	1	0	1	g	6	Warmblood
door_gate_Engl.	2380		1	2	1	1	4	0	0	0	1	2	1	1	0	1	0	0	1	0	0	0	0	0	3	1	1	1	1	1	1	f	18	Thorough bred
door_gate_Engl.	2395		2	2	2	2	4	0	0	0	1	2	1	1	0	1	0	0	1	0	0	0	0	0	3	1	1	1	0	1	1	f	16	Warmblood
door_gate_Engl.	2398	picture	1	1	1	1	4	0	0	0	1	1	1	0	0	1	0	0	1	0	0	0	0	0	1	1	1	0	0	0	0	f	3	Pony
door_gate_Engl.	2403		1	2	2	1	2	0	0	0	2	2	1	0	1	1	0	0	0	1	1	0	0	0	2	1	0	0	1	1	0	g	19	Warmblood
door_gate_Engl.	2414	video	1	2	1	1	2	1	0	0	1	1	1	0	0	1	0	0	0	1	0	0	0	0	1	0	1	0	0	0	0	s	21	Arabian horse
door_gate_Engl.	2414a		1	2	1	1	2	0	1	1	1	1	1	0	0	1	0	0	0	1	0	0	0	0	1	0	1	0	0	0	0	f		Arabian horse
door_gate_Engl.	2423		2	2	2	2	2	0	0	0	1	2	1	1	0	1	0	0	0	1	0	0	0	0	3	1	0	1	1	1	1	f	13	Coldblood
door_gate_Engl.	2423a		2	2	2	2	2	0	0	0	1	2	1	1	0	1	0	0	0	1	0	0	0	0	3	1	1	0	1	0	1	g		Warmblood
door_gate_Engl.	2424		1	2	2	1	2	0	0	0	3	1	0	0	1	0	0	1	0	0	1	1	2	0	2	1	0	0	1	1	0	g	8	Warmblood
door_gate_Engl.	2429	video	1	2	1	1	4	0	0	0	2	1	1	0	0	1	0	0	1	1	0	0	0	0	1	1	1	0	1	0	0	g	5	Warmblood
door_gate_Engl.	2430		2	1	1	2	2	0	0	0	1	1	1	0	0	1	0	0	1	0	0	0	0	0	1	1	0	0	1	0	0	g	4	Pony
door_gate_Engl.	2438		2	1	2	2	2	0	0	0	1	1	1	0	0	1	0	0	1	0	0	0	0	1	1	0	1	0	1	0	0	f	2	Arabian horse
door_gate_Engl.	2440		2	2	2	2	5	1	0	0	3	1	0	0	1	0	0	0	0	1	0	1	0	1	3	1	0	1	1	1	1	g	6	Warmblood
door_gate_Engl.	2458		2	2	2	2	4	0	0	0	3	1	0	0	1	0	0	1	1	1	0	1	0	0	3	1	0	0	1	0	1	g	30	Warmblood
door_gate_Engl.	2478	video	1	2	1	1	5	0	0	0	1	1	1	0	0	1	0	0	1	0	0	0	0	0	1	0	1	1	1	1	1	g	14	Arabian horse
door_gate_Engl.	2478a		1	2	1	1	5	0	0	0	1	1	1	0	0	1	0	0	1	0	0	0	0	0	1	0	1	1	1	1	1	f		Arabian horse
door_gate_Engl.	2505	picture	2	1	2	2	4	0	0	0	1	1	0	0	1	0	0	1	1	0	0	0	0	0	3	1	0	1	1	1	1	g	2	Arabian horse
door_gate_Frensh	2459		1	1	1	1	5	0	0	0	1	1	1	0	0	1	0	0	1	0	0	0	0	0	1	1	0		0	0	0	g	7	Warmblood
door_gate_Frensh	2471	picture	1	1	1	1	5	0	0	0	1	2	1	1	0	1	0	0	1	0	0	0	0	0	2	1	1	0	1	0	1	s	6	NA
door_gate_Frensh	2469	picture	1	1	1	1	2				1	2	1	1	0	1	0	0	0	1	0	0	0	0	3	1	0	1	1	1	1	g	17	Pony
door_gate_Frensh	2468		1	2	1	1	4	0	1	1	2	2	1	1	0	1	0	0	1	1	0	0	0	0	2	1	1	0	0	1	0	f	8	Warmblood
door_gate_Frensh	2467	picture	1	1	1	1	4	0	0	0	1	2	1	1	0	1	0	0	1	0	0	0	0	0	3	1	1	1	1	1	1	g	2	Warmblood
door_gate_Frensh	2466		1	1	1	1	3		1	1	2	1	1	0	0	1	0	0	1	1	0	1	0	0	1	1	1	0	0	0	0	f	1	Arabian horse
door_gate_Frensh	2466a		1	1	1	1	3	1	0	0	1	1	1	0	0	1	0	0	1	1	0	0	0	0	1	1	1	0	0	0	0	f	2	Arabian horse
door_gate_Frensh	2465		1	2	1	1	2		0	0	1	2	1	1	0	1	0	0	1	0	0	0	0	0	2	1	1	0	1	1	0	g	14	Warmblood
door_gate_Frensh	2464	picture	1	1	1	1	5		1	1	1	1	1	0	0	1	0	0	1	0	0	1	0	0	1	1	0	0	1	0	0	g	10	Pony
door_gate_Frensh	2463		2	1	2	2	3	0	0	0	2	3	1	1	0	1	1	0	0	1	0	0	0	0	2	0	1	0	0	1	0	g	28	NA
door_gate_Frensh	2462		1	2	1	1	2				1	1	1	0	0	1	0	0	1	0	0	0	0	0	1	1	1	0	1	0	0	g	13	Warmblood
door_gate_Frensh	2461		1	1	1	1	5	0	0	0	2	2	1	1	0	1	0	0	1	0	1	0	0	0	2	1	1	0	0	1	0	g	26	Warmblood
door_gate_Frensh	2460		1	1	1	1	4	0	0	0	1	2	1	1	0	1	0	0	1	0	0	1	0	0	2		1	0	1	1	0	g	8	NA
door_gate_Frensh	2472	picture	1	2	1	1	5	0	0	0	1	1	1	0	0	1	0	0	1	0	0	0	0	0	1	0	1	0	0	0	0	f	8	Pony
door_gate_Frensh	2473		1	1	2	1	5	0	0	0	1	1	1	0	0	1	0	0	1	0	0	0	0	0	1	1	0	0	0	0	0	f		Arabian horse
door_gate_Frensh	2474		2	1	2	2	1				2	2	1	0	0	1	1	0	0	1	0	0	0	0	1							f	6	Warmblood
door_gate_Frensh	2476		2	2	2	2		0	0	0	2	3	1	1	0	1	1	0	0	0	1	0	0	0	2	1	0	1	1	0	0	g	18	Arabian horse
door_gate_Frensh	2477	picture	1	1	1	1		0	0	0	1	2	1	1	0	1	0	0	1	0	0	0	0	0	2	1	0	0	1	0	0	g	7	Warmblood
door_gate_Frensh	2479		1	1	1	1	5	0	0	0	2	2	1	0	0	1	1	0	0	1	0	0	0	0	1	1	1	0	1	0	0	g	13	Warmblood
door_gate_Frensh	2480		2	2	2	2	2	1	1	1	1	2	1	1	0	1	0	0	0	1	0	0	0	0	2	1	0	0	1	1	0	g	24	Haflinger
door_gate_Frensh	2481		1	1	1	1	3		0	0	2	2	1	1	0	1	0	0	1	0	0	0	0	1	2	1	1	1	1	0	1	g	11	Warmblood
door_gate_Frensh	2482		1	2	1	1	1	0	1	1	1	2	1	1	0	1	0	0	1	0	0	0	0	0	2	1	0	0	0	1	0	g		NA
door_gate_Frensh	2483	picture	2	1	1	2	5	1	0	0	3	1	1	0	0	1	0	0	0	0	1	0	1	1	1	1	0	0	0	0	0	f	5	NA
door_gate_Frensh	2483a	picture	2	1	1	2	5	0	1	1	1	2	1	1	0	1	0	0	0	0	0	0	1	0	2	1	0	0	1	0	1	f		Pony
door_gate_Frensh	2482		1	1	1	1	2	2	2	1	1	1	1	0	0	1	0	0	1	0	0	0	0	0	1	1	1	0	1	0	0	f	9	Warmblood
door_gate_Frensh	2485	picture	1	1	1	1	2	0	0	0	1	1	0	0	1	0	0	1	0	0	0	0	2	0	2	1	1	0	1	1	0	g	12	Arabian horse
door_gate_Frensh	2486		1	2	1	1	5	0	0	0	2	2	1	1	0	1	0	0	1	1	0	1	0	0	2	1	1	0	1	1	1	g	4	Warmblood
door_gate_Frensh	2486a		1	2	1	1	5	0	0	0	1	1	0	0	1	0	0	0	0	1	0	0	0	0	1	1	1	0	1	0	0	f		Warmblood
door_gate_Frensh	2487		1	1	1	1	2	0	0	0	2	2	1	1	0	1	0	0	0	1	1	0	0	0	2	1	1	0	1	1	0	g	10	Warmblood
door_gate_Frensh	2488	picture	1	1	1	1	5	0	0	0	1	2	1	1	0	1	0	0	1	0	0	0	0	0	2	1	0	0	1	0	1	f	20	Warmblood
door_gate_Frensh	2489		2	2	2	2	4	0	0	0	1	1	0	1	0	1	0	0	0	0	0	0	1	0	1	0	1	0	0	1	0	f	16	Warmblood
door_gate_Frensh	2490		1	1	1	1	5	0	1	1	2	2	1	1	0	1	0	0	1	0	0	1	0	0	3	1	0	1	1	1	1	f	11	Warmblood
door_gate_Frensh	2491		1	1	1	1	5	1	2	1	2	2	1	1	0	1	0	0	1	0	0	0	1	0	4	1	0	0	1	1	0	g	6	Warmblood
door_gate_Frensh	2493		2	2	2	2	1	0	0	0	1	1	0	0	0	0	0	1	1	0	0	0	0	0	1	0	0	0	0	1	0	g	5	Warmblood
door_gate_Frensh	2494	picture	2	2	2	2	2	0	0	0	1	1	0	0	1	0	0	1	1	0	0	0	0	0	2	1	1	0	1	1	0	f	8	Pony
door_gate_Frensh	2495		1	1	1	1	2	0	0	0	1	1	1	0	0	1	0	0	1	0	0	0	0	0	2	1		0	0	1	0	g	9	Warmblood
door_gate_Frensh	2496		1	1	1	1	5	0	0	0	3	3	1	1	0	1	1	0	1	1	0	0	0	0	3	1	1	0	1	1	0	g	12	Warmblood
door_gate_Frensh	2497		2	2	2	2	4	0	0	0	1	1	0	1	0	1	0	0	0	0	0	0	1	0	1	0	1	0	0	1	0	g	6	Arabian horse
door_gate_Frensh	2501	picture	2	2	2	2	4	0	0	0	1	1	0	0	1	0	0	1	1	0	0	0	0	0	1	1	0	0	1	1	0	f	14	Pony
door_gate_Frensh	2503	picture	2	1	1	2	4	0	0	0	3	3	1	1	1	1	0	1	1	1	0	0	2	0	3	1	0	0	1	0	1	g	8	Warmblood
