# Supplementary material for: Animal behaviour in a human world: A crowdsourcing study on horses that open door and gate mechanisms
Source: PLoS One. 2019 Jun 26;14(6):e0218954. doi: 10.1371/journal.pone.0218954 (PMC6594629; doi:10.1371/journal.pone.0218954)
Supplement: S2 Table — Please enlarge Pdf for viewing the data. (PDF) [file pone.0218954.s006.pdf]

Supporting information, S2 Table. Data videos.
Animal behaviour in a human world: A crowd sourcing study on horses that open door and gate mechanisms
Krueger K, Esch L, Byrne R

Questionaire	sID	single / group stabling	amount of feed	access to pasture	social contact	different mechsanims opened	own door	other doors	gate	sliding doors	bolt sideways	bolt upwards / downwards	twist mechanism	opens security chain/rope	handle	carabina	nr of mechansim positions	goes out of places	stays in the stable	goes into other horses boxes	runs around freely	goes into feed or other rooms	frees other horses	sex	age	breed -type	contact
gen.Germ.	2071	1				1	1	0	0	0	1	0	0	0	0	0	1	1		0		0				Warmblood	Ein schlaues Pferd - YouTube [720p].mp4
gen.Germ.	2075					1	0	1	0	0	1	0	0	0	0	0	1	0		1		0				Warmblood	https://www.youtube.com/watchNAv=YVMej_ApKMw
gen.Germ.	2079	1				1	1	0	0	0	1	0	0	0	0	0	1	1		0		0		g		Warmblood	http://youtu.be/DuleXhBpZKs
gen.Germ.	2080	1				1	1	0	0	0	1	0	0	1	0	0	1	1		0		0		f		Warmblood	http://youtu.be/LS69RAiOOus
gen.Germ.	2081	1				1	1	0	0	0	1	0	0	0	0	0	1	1		0		0		f		Warmblood	http://youtu.be/_UIByc0rIyA
gen.Germ.	2083	1				1	1	0	0	0	1	0	0	0	0	0	1	1		0		0		f		Arabian horse	http://youtu.be/m5uQQyRKubI
gen.Germ.	2084	1				1	1	0	0	0	1	0	0	0	0	0	1	1		0		0		f		Haflinger	http://youtu.be/a1IAVrKHtIA
gen.Germ.	2085	1				1	1	1	0	0	1	0	0	0	0	0	1	1		1		0				Warmblood	http://youtu.be/1XWFjUjNq3E
gen.Germ.	2086					1	1	0	0	0	1	0	0	0	0	0	1	1		0		0		f		Warmblood	http://youtu.be/Ntydqyo6D8o
gen.Germ.	2087					1	1	0	0	0	1	0	0	0	0	0	1	1		0		0		g		Warmblood	http://youtu.be/awuHaxptcgc
gen.Germ.	2088					1	1	0	0	0	1	0	0	0	0	0	1	1		0		0		g		Warmblood	http://youtu.be/8uJ05KN4rhg
gen.Germ.	2090					1	0	0	1	0	1	0	0	0	0	0	1	1		0		0		f		Quarter Horse	http://youtu.be/BLejxIfZkkA
gen.Germ.	2091					1	0	0	1	0	0	1	0	0	0	0	1	1		0		0		g		Warmblood	http://youtu.be/EJDoCMj-WlQ
gen.Germ.	2093					1	0	1	0	0	0	0	0	0	1	0	1	0		0		1				Pony	http://youtu.be/7Zf4lbMe7VM
gen.Germ.	2096					2	1	0	0	1	0	1	0	0	1	0	2	1		0		1				NA	http://youtu.be/90Xqn0uUnaY
gen.Germ.	2103					1	1	0	0	0	1	0	0	0	0	0	1	1		0		0				Arabian horse	http://youtu.be/vFc9bQ0Vs3Q
gen.Germ.	2104					1	0	0	1	0	0	1	0	0	0	0	1	1		0		0		g		Warmblood	http://youtu.be/UnuuukezIUw
gen.Germ.	2106					1	1	0	0	0	1	0	0	0	0	0	1	1		0		0		g		Warmblood	http://youtu.be/0SDpaxiIWfI
gen.Germ.	2107					1	1	0	0	0	1	0	0	0	0	0	1	1		0		0		g		Warmblood	http://youtu.be/nY2owCKXAr4
gen.Germ.	2109					1	0	1	0	0	1	0	0	0	0	0	0	0		1		0		g		Warmblood	http://youtu.be/MTg4M5D3fH4
gen.Germ.	2113					1	1	0	0	0	1	0	0	0	0	0	1	1		0		0				Warmblood	http://youtu.be/M-LvDjityTM
gen.Germ.	2121					0	0	0	1	0	1	0	0	0	0	0	1	1		0		0		g		Warmblood	http://youtu.be/T7wi6Bt8O3g
gen.Germ.	2131					1	1	0	0	0	1	0	0	0	0	0	1	1		0		0		f		Warmblood	http://youtu.be/osbxMbHUNe8
gen.Germ.	2133					2	1	0	0	1	0	1	0	0	0	0	2	1		0		0		g		Arabian horse	http://youtu.be/m2B3_qwwgIM
gen.Germ.	2134					1	1	0	0	0	1	0	0	0	0	0	1	1		0		0				NA	http://youtu.be/jyWR6F-EaeY
gen.Germ.	2135					1	1	0	0	0	1	0	0	0	0	0	1	1		0		0		g		Warmblood	http://youtu.be/AzLToOWyXyk
gen.Germ.	2136					1	0	1	0	0	0	0	0	0	1	0	1	0		0		1		f		Warmblood	http://youtu.be/1yLYMfwFqis
gen.Germ.	2137					1	0	1	0	0	0	0	0	0	1	0	1	0		0		1		g		Pony	http://youtu.be/TipjTiYOgE4
gen.Germ.	2138					1	0	0	1	0	1	0	0	0	0	0	2	1		0		0				NA	http://youtu.be/b0BUqwqLI3wNAt=31s
door_gate_Germ.	2181					1	0	0	1	0	1	0	0	0	0	0	1	1	0	0	1	0	0			Warmblood	https://www.youtube.com/resultsNAq=clever+horse&sp=SCjqAwA%253D
door_gate_Germ.	2179	1				1	1	1	0	0	1	0	0	0	1	0	2	1	0	1	1	1	1	f		Arabian horse	https://www.youtube.com/watchNAv=nDmEs_gRKgQ
door_gate_Germ.	2178	2				1	1	0	0	0	1	0	0	0	0	0	1	1	0	1	1	1		g		Warmblood	https://www.youtube.com/watchNAv=O8M1iKIaXpc
door_gate_Germ.	2176	1				1	1	1	0	0	1	0	0	0	0	0	2			1	1	0		s		Warmblood	https://www.youtube.com/watchNAv=LZ91Ig3yZks
door_gate_Germ.	2177	1				1	1	0	0	0	1	0	0	0	0	0	1	1	0		1			f		Arabian horse	https://www.youtube.com/watchNAv=6pibfNWwVXg
door_gate_Germ.	2204	1				1	1	0	0	0	1	0	0	0	0	0	1	1	0		1					Warmblood	https://www.youtube.com/watchNAv=Y6580YcROyI
door_gate_Germ.	2205					1	0	0	1	0	0	0	0	0	2	0	2									Warmblood	https://www.youtube.com/watchNAv=lxMuQiFQN98
door_gate_Engl.	2206	1				2	1	0	0	1	1	0	0	0	0	0	2							g		Warmblood	https://www.youtube.com/watchNAv=ez6nOMYfcIc
door_gate_Engl.	2207	1				1	1	0	0	0	1	0	0	0	0	0	1							g		Warmblood	https://www.youtube.com/watchNAv=owjvgT8H4g4
door_gate_Engl.	2208	1				1	1	0	0	0	1	0	0	0	0	0	1									Warmblood	https://www.youtube.com/watchNAv=d_xe1INWkq0
door_gate_Engl.	2209	1				2	1	0	0	1	0	1	0	0	0	0	2							f		Arabian horse	https://www.youtube.com/watchNAv=5XUuQ68bbh0
door_gate_Germ.	2512	1	1	1	1	1	1	0	0	1	0	1	0	0	0	0	1	1	1	1	0	0	1			Warmblood	https://www.youtube.com/watchNAv=IXebjTbLT4M
door_gate_Germ.	2513	1		1	1	1	1	0	0	0	1	0	0	0	0	0	1	1	0		1					Warmblood	https://www.youtube.com/watchNAv=-R_e-zd5zr0
door_gate_Germ.	2514	1	1	1	1	1	1	0	0	0	1	0	0	0	0	0	2	1	0	1	1	0	1	g		Haflinger	https://www.youtube.com/watchNAv=krnNwRdlkJo
door_gate_Germ.	2515	1	1	1	1	1	1	0	0	0	1	0	0	0	0	0	1	1	1	0	0	0	0	g	6	Arabian horse	https://www.youtube.com/watchNAv=bo60IBvt5jw
door_gate_Germ.	2516	1	1	1	1	1	1	0	0	0	1	0	0	0	0	0	1	1	1	0	0	0	0	g		Warmblood	https://www.youtube.com/watchNAv=qBjlyl96E3g
door_gate_Germ.	2517					1	0	0	1	0	1	0	0	0	0	0	1	0	0	1			1			pony	https://www.youtube.com/watch?v=rZLbI5xcV9E
door_gate_Germ.	2518	1	1	1	1	1	1	1	0	0	1	0	0	0	0	0	2	1	0	1	0	1	1			Warmblood	https://www.youtube.com/watch?v=W3KnTX0RI5s
door_gate_Germ.	2538	1	1	1	1	1	1	0	0	0	1	0	0	0	0	0	1	1		0			1			Warmblood	https://www.youtube.com/watch?v=8IE1HHGuJ-E
door_gate_Germ.	2539	1		1	1	1	1	0	0	1	0	1	0	0	0	0	2							f		Warmblood	https://www.youtube.com/watch?v=QnVISUkqMAo
door_gate_Germ.	2519	1	1	1	1	1	0	1	0	1	1	0	0	0	0	0	1	1	0	0	0	1	0			Warmblood	https://www.youtube.com/watch?v=0qUFaYN8-Ec
door_gate_Germ.	2520	1		1	1	1	1	0	0	0	0	0	0	0	0	1	1									Warmblood	https://www.youtube.com/watch?v=uI-u7-_wA94
door_gate_Germ.	2521	1	1	1	1	1	1	0	0	0	1	0	0	0	0	0	1	0	0	0	0	0	1			Warmblood	https://www.youtube.com/watch?v=A9V-ePJZfWo
door_gate_Germ.	2522	1	1	1	1	1	0	0	1	0	1	0	0	0	0	0	1	0		1			1			Pony	https://www.youtube.com/watch?v=m3cIQNlGBQ4
door_gate_Germ.	2523					1	1	0	0	0	0	0	0	0	0	1	1								0.5	NA	https://www.youtube.com/watch?v=YUe9gmUVQ6U
door_gate_Germ.	2524	1	1	1	1	1	1	0	0	0	0	0	0	0	0	1	1									Warmblood	https://www.youtube.com/watch?v=Fg-wViXmc1Y
door_gate_Germ.	2525	2			2	1	0	0	1	0	1	0	0	0	0	0	1	0		1			1			Warmblood	https://www.youtube.com/watch?v=VqYcJrye6VU
door_gate_Germ.	2526	1		1	1	1	1	0	0	0	0	1	0	0	0	0	1									Warmblood	https://www.youtube.com/watch?v=g_DzG4vkmHc
door_gate_Germ.	2527	1		1	1	1	1	0	0	0	1	0	0	0	0	0	1							g		Warmblood	https://www.youtube.com/watch?v=zwsm07q3y7M
door_gate_Germ.	2528					1	0	1	0	0	0	0	0	0	1	0	1									pony	https://www.youtube.com/watch?v=EKAROqJvYDs
door_gate_Germ.	2529					1	0	1	0	0	0	0	0	0	1	0	1									pony	https://www.youtube.com/watch?v=Iu8LwIvZqvk
door_gate_Germ.	2530					1	0	0	1	0	0	0	0	1	0	0	1							g		Warmblood	https://www.youtube.com/watch?v=rDU4BlJFj9c
door_gate_Germ.	2531	1		1	1	1	1	0	0	0	1	0	0	0	0	0	1									Warmblood	https://www.youtube.com/watch?v=AUGKbhZGTJw
door_gate_Germ.	2532					1	0	0	1	0	0	1	0	0	0	0	1							f		Pony	https://www.youtube.com/watch?v=8JIrxgDxyZ0
door_gate_Germ.	2533					1	0	1	0	1	0	0	0	0	1	0	1									Pony	https://www.youtube.com/watch?v=G1rqmvbJcrs
door_gate_Germ.	2534	1	1	1	1	1	1	0	0	0	0	1	0	0	0	0	1							g		Warmblood	https://www.youtube.com/watch?v=llwN-PxdTrw
door_gate_Germ.	2535					1	0	1	0	0	0	0	0	0	1	0	1									Pony	https://www.youtube.com/watch?v=3iJMVjydtcM
door_gate_Germ.	2536	1	1	1	1	1	1	0	0	0	1	0	0	0	0	0	1									Warmblood	https://www.youtube.com/watch?v=F-ZWQt7Htfw
door_gate_Germ.	2537					1	0	1	0	0	0	0	0	0	1	0	1									Warmblood	https://www.youtube.com/watch?v=rDMYQbe5Mwk
door_gate_Germ.	2540					3	1	0	0	0	1	1	0	0	0	1	3									warmblood	https://www.youtube.com/watch?v=-nndf7Mhc_o
