# Supplementary material for: Animal behaviour in a human world: A crowdsourcing study on horses that open door and gate mechanisms
Source: PLoS One. 2019 Jun 26;14(6):e0218954. doi: 10.1371/journal.pone.0218954 (PMC6594629; doi:10.1371/journal.pone.0218954)
Supplement: S4 Table — Please enlarge Pdf for viewing the data. (PDF) [file pone.0218954.s008.pdf]

Supporting information, S4 Table. Data mules and donkeys door opening.

Animal behaviour in a human world: A crowd sourcing study on horses that open door and gate mechanisms

Krueger K, Esch L, Byrne R

Data survey

Questiona	sID	pictures / videos attached	single / group stabling	amount of feed	access to pasture	social contact	how often was the behaviour shown	behaviour spreading to other horses	other horses in stable show same behaviour	nr of differnt locks opened	nr of different doors opened	own door	other doors	gate	sliding doors	bolt sideways	bolt upwards / downwards	twist mechanism	opens security chain / rope	handle	carabina	nr of mechanism positions	goes out of places	stays in the stable	goes into other horses boxes	runs around freely	goes into feed or other room	frees other horses	sex	age in years	breed -type
door_gate	2368	NA	2	1	2	2	4	0	0	2	2	1	1	0	0	1	1	0	0	0	0	2	1	0	0	0	1	1	f	21	Donkey
door_gate	2379	picture	2	1	2	2	2	NA	NA	3	3	1	1	1	0	1	1	0	0	1	1	4	1	0	NA	1	1	NA	NA	1	Mule

Data videos

Questionaire	sID	single / group stabling	amount of feed	access to pasture	social contact	nr of different mechanisms opened	own door	other doors	gate	sliding doors	bolt sideways	bolt upwards / downwards	twist mechanism	opens security chain/rope	handle	carabina	nr of mechanismp ositions	goes out of places	stays in the stable	goes into other horses boxes	runs around freely	goes into feed or other rooms	frees other horses	sex	age	breed -type	contact
door_gate_Germ.	2510	2	1	2	2	1	0	1	0	0	0	0	0	0	1	0	1	1	1	0	0	1	0	NA	NA	Donkey	https://www.youtube.com/watchNAv=sa5cgpMO0xA
door_gate_Engl.	2507	2	NA	1	2	1	0	0	1	0	1	0	0	0	0	0	1	1	0	NA	1	NA	1	NA	NA	Donkey	https://www.youtube.com/watchNAv=AN6l0Ehu7O0
door_gate_Engl.	2508	1	NA	2	1	1	0	0	1	0	0	1	0	0	0	0	1	1	NA	NA	1	0	0	NA	NA	Donkey	https://www.youtube.com/watchNAv=xEfotAYK_TE
door_gate_Engl.	2511	1	1	1	1	1	0	0	1	0	1	0	0	0	0	0	1	1	NA	NA	1	NA	0	g	NA	Mule	https://www.youtube.com/watchNAv=QiEYv4fA9vE

Movement counts video

Questiona	sID	nr diff doors / barrier types	nr diff mechanism types	nr mechanism positions	sliding doors	door or gate on hinges	electric fence handle	bolt sideways	bolt upwards / downwards	twist mechanism	security chain / rope	handle	carabina	mechanism nr	mechanism type	gate movement direction (plane)	mechanism movement diretion	head /mouth movement opening	movement mechanism /door	movement mechanism /door simpl	ordered sequence mechanism opening	movements for mechanism / door opening	movements neccessarily needed	total nr of movement sequences	total nr of movements	% of movements neccessary	source
door_gate	2510	1	1	1	0	1	0	0	0	0	0	1	0	1	handle	opposite	orth	linear	vertical	vertical	1	1	no				
															handle	opposite	orth	linear	vertical	vertical	2	2	no				
															handle	opposite	orth	linear	vertical	vertical	3	3	yes				
															hinge door	opposite	0	linear	forward-back	horizontal	3	4	yes	3	4	0,5	https://www.youtube.com/watch?v=owjvgT8H4g4
door_gate	2507	1	1	1	0	1	0	1	0	0	0	0	0	1	bosd / bar	orth	orth	linear	vertical	vertical	1	1	yes	1	1	1	https://www.youtube.com/watch?v=xEfotAYK_TE
door_gate	2508	1	1	1	0	1	0	0	1	0	0	0	0	1	boupdo	same	orth	twist	vertical	vertical	1	1	yes				
															boupdo	same	orth	twist	left-right	horizontal	1	2	yes				
															boupdo	same	orth	twist	vertical	vertical	2	3	no				
															boupdo	same	orth	twist	vertical	vertical	2	4	no				
															boupdo	same	orth	twist	vertical	vertical	2	5	no				
															boupdo	same	orth	twist	vertical	vertical	2	6	no				
															boupdo	same	orth	twist	vertical	vertical	2	7	no				
															boupdo	same	orth	twist	vertical	vertical	2	8	no				
															boupdo	same	orth	twist	vertical	vertical	2	9	no				
															boupdo	same	orth	twist	vertical	vertical	2	10	no				
															boupdo	same	orth	twist	left-right	horizontal	2	11	no				
															hinge door	same	0	linear	forward-back	horizontal	3	12	yes	3	12	0,25	https://www.youtube.com/watch?v=QiEYv4fA9vE
door_gate	2511	1	1	1	0	1	0	1	0	0	0	0	0	1	bosd	opposite	orth	linear	left-right	horizontal	1	1	yes				
															bosd	opposite	orth	linear	left-right	horizontal	2	2	no				
															bosd	opposite	orth	linear	left-right	horizontal	2	3	no				
															hinge door	opposite	0	linear	forward-back	horizontal	3	4	yes				
															hinge door	opposite	0	linear	forward-back	horizontal	4	5	no	4	5	0,4	https://www.youtube.com/watch?v=IXebjTbLT4M
