# Supplementary material for: Animal behaviour in a human world: A crowdsourcing study on horses that open door and gate mechanisms
Source: PLoS One. 2019 Jun 26;14(6):e0218954. doi: 10.1371/journal.pone.0218954 (PMC6594629; doi:10.1371/journal.pone.0218954)
Supplement: S3 Table — Please enlarge Pdf for viewing the data. (PDF) [file pone.0218954.s007.pdf]

Supporting information, S3 Table. Data movement counts videos.
Animal behaviour in a human world: A crowd sourcing study on horses that open door and gate mechanisms
Krueger K, Esch L, Byrne R

Questionaire	sID	nr diff doors / barrier types	nr diff mechasnism types	nr mechanism positions	sliding doors	door or gate on hinges	barred door or gateway	bolt sideways	bolt upwards / downwards	twist mechasnism	security chain / rope	handle	carabina	mechasnim nr	mechsanim type	door / gate movement direction (plane)	mechasnim movement diretion	head /mouth movement opening	movement mechansim /door	movement mechanism /door simpl	ordered sequence mechasnimopening	nr of movements for mechasnim / door opening	Nr of movements neccessarily needed	total nr of movement sequences	total nr of movements	% of movements neccessary
gen.Engl.	2039	1	1	1	0	1	0	1	0	0	0	0	0	1	bosd	same	orth	linear	left-right	horizontal	1	1	yes	3	6	0,33
															bosd	same	orth	linear	left-right	horizontal	1	2	no			
															bosd	same	orth	linear	left-right	horizontal	2	3	no			
															bosd	same	orth	linear	left-right	horizontal	2	4	no			
															bosd	same	orth	linear	left-right	horizontal	2	5	no			
															hinge door/gate	same		linear	forward-back	horizontal	3	6	yes			
gen.Germ.	1988	1	1	3	0	0	1	0	0	0	0	2	0	1	ehandle	orth	orth	linear	left-right	horizontal	1	1	yes	11	15	0,86
															ehandle	orth	orth	linear	left-right	horizontal	1	2	no			
															ehandle	orth	orth	linear	vertical	vertical	1	3	yes			
															ehandle	orth	orth	linear	vertical	vertical	2	4	yes			
															ehandle	orth	orth	linear	left-right	horizontal	3	5	yes			
														2	ehandle	orth	orth	linear	left-right	horizontal	1	1	yes			
															ehandle	orth	orth	linear	vertical	vertical	1	2	yes			
															ehandle	orth	orth	linear	vertical	vertical	2	3	yes			
															ehandle	orth	orth	linear	left-right	horizontal	3	4	yes			
														3	ehandle	orth	orth	linear	vertical	vertical	1	1	no			
															ehandle	orth	orth	linear	vertical	vertical	2	2	no			
															ehandle	orth	orth	linear	left-right	horizontal	3	3	yes			
															ehandle	orth	orth	linear	vertical	vertical	3	4	yes			
															ehandle	orth	orth	linear	vertical	vertical	4	5	yes			
															ehandle	orth	orth	linear	left-right	horizontal	5	6	yes			
gen.Germ.	1990	2	3	6	1	1	0	1	1	0	0	0	0	1	bosd	same	orth	linear	left-right	horizontal	1	1	yes	2	3	0,66
															bosd	same	orth	linear	left-right	horizontal	1	2	no			
															hinge door/gate	same		linear	forward-back	horizontal	2	3	yes			
														2	bosd	opposite	orth	linear	forward-back	horizontal	1	1	yes	2	3	0,66
															bosd	opposite	orth	linear	forward-back	horizontal	1	2	no			
															hinge door/gate	opposite		linear	forward-back	horizontal	2	3	yes			
														3	bosd	opposite	orth	linear	left-right	horizontal	1	1	yes	2	4	0,5
															bosd	opposite	orth	linear	left-right	horizontal	1	2	no			
															bosd	opposite	orth	linear	left-right	horizontal	1	3	no			
															hinge door/gate	opposite		linear	forward-back	horizontal	2	4	yes			
														4	boupdo	orth	orth	linear	left-right	horizontal	1	1	yes	4	8	0,25
															boupdo	orth	orth	linear	vertical	vertical	1	2	no			
															boupdo	orth	orth	linear	vertical	vertical	1	3	no			
															boupdo	orth	orth	linear	vertical	vertical	2	4	no			
															boupdo	orth	orth	linear	vertical	vertical	2	5	no			
															boupdo	orth	orth	linear	vertical	vertical	2	6	no			
															slide door	orth		linear	left-right	horizontal	1	1	yes			
															slide door	orth		linear	left-right	horizontal	2	2	no			
														5	boupdo	orth	orth	twist	vertical	vertical	1	1	yes	4	4	0,75
															slide door	orth		linear	left-right	horizontal	1	1	yes			
															slide door	orth		linear	left-right	horizontal	2	2	no			
															slide door	orth		linear	left-right	horizontal	1	1	yes			
															slide door	orth		linear	left-right	horizontal	2	2	no			
														6	hinge door/gate	opposit		linear	forward-back	horizontal	1	1	yes			
gen.Germ.	2071	1	1	1	0	1	0	1	0	0	0	0	0	1	bosd	same	orth	linear	left-right	horizontal	1	1	no	5	6	0,5
															bosd	same	orth	linear	left-right	horizontal	2	2	no			
															bosd	same	orth	linear	left-right	horizontal	3	3	no			
															bosd	same	orth	linear	vertical	vertical	4	4	yes			
															bosd	same	orth	linear	left-right	horizontal	4	5	yes			
															hinge door/gate	same		linear	forward-back	horizontal	5	6	yes			
gen.Germ.	2075	1	1	1	0	1	0	1	0	0	0	0	0	1	bosd	opposit	orth	linear	left-right	horizontal	1	1	no	5	8	0,375
															bosd	opposit	orth	linear	left-right	horizontal	1	2	no			
															bosd	opposit	orth	linear	vertical	vertical	2	3	no			
															bosd	opposit	orth	linear	left-right	horizontal	2	4	no			
															bosd	opposit	orth	linear	vertical	vertical	3	5	yes			
															bosd	opposit	orth	linear	left-right	horizontal	3	6	yes			
															hinge door/gate	opposit		linear	forward-back	horizontal	4	7	yes			
															hinge door/gate	opposit		linear	forward-back	horizontal	5	8	no			
gen.Germ.	2079	1	1	1	0	1	0	1	0	0	0	0	0	1	boupdo	same	orth	linear	vertical	vertical	1	1	yes	3	7	0,43
															boupdo	same	orth	linear	vertical	vertical	1	2	no			
															boupdo	same	orth	linear	vertical	vertical	1	3	no			
															boupdo	same	orth	linear	vertical	vertical	1	4	no			
															hinge door/gate	same		linear	forward-back	horizontal	2	5	yes			
															hinge door/gate	same		linear	forward-back	horizontal	2	6	no			
															hinge door/gate	same		linear	forward-back	horizontal	3	7	no			
gen.Germ.	2080	1	2	1	0	1	0	1	0	0	0	0	0	1	bosd	same	blankets	linear	vertical	vertical	1	1	yes	10	11	0,9
															bosd	same	blankets	linear	vertical	vertical	2	2	yes			
															bosd	same	blankets	linear	vertical	vertical	3	3	yes			
															bosd	same	blankets	linear	vertical	vertical	4	4	yes			
															bosd	same	blankets	linear	vertical	vertical	5	5	yes			
															bosd	same	blankets	linear	vertical	vertical	6	6	yes			
														2	bosd	same	orth	linear	vertical	vertical	7	7	yes			
															bosd	same	orth	linear	left-right	horizontal	7	8	yes			
															bosd	same	orth	linear	vertical	vertical	8	9	no			
															hinge door/gate	same		linear	forward-back	horizontal	9	10	yes			
															hinge door/gate	same		linear	forward-back	horizontal	10	11	no			
gen.Germ.	2081	1	1	1	0	1	0	1	0	0	0	0	0	1	bosd	same	orth	linear	vertical	vertical	1	1	no	6	8	0,375
															bosd	same	orth	linear	vertical	vertical	2	2	no			
															bosd	same	orth	linear	vertical	vertical	3	3	no			
															bosd	same	orth	linear	vertical	vertical	4	4	no			
															bosd	same	orth	linear	left-right	horizontal	4	5	no			
															bosd	same	orth	linear	vertical	vertical	5	6	yes			
															bosd	same	orth	linear	left-right	horizontal	5	7	yes			
															hinge door/gate	same		linear	forward-back	horizontal	6	8	yes			
gen.Germ.	2083	1	1	1	0	1	0	1	0	0	0	0	0	1	bosd	same	orth	linear	vertical	vertical	1	1	no	3	4	0,75
															bosd	same	orth	linear	vertical	vertical	2	2	yes			
															bosd	same	orth	linear	left-right	horizontal	2	3	yes			
															hinge door/gate	same		linear	forward-back	horizontal	3	4	yes			
gen.Germ.	2084	1	1	1	0	1	0	1	0	0	0	0	0	1	bosd	same	orth	linear	left-right	horizontal	1	1	no	4	5	0,6
															bosd	same	orth	linear	left-right	horizontal	2	2	no			
															bosd	same	orth	linear	vertical	vertical	3	3	yes			
															bosd	same	orth	linear	left-right	horizontal	3	4	yes			
															hinge door/gate	same		linear	forward-back	horizontal	4	5	yes			
gen.Germ.	2085	1	1	2	0	1	0	1	0	0	0	0	0	1	bosd	same	orth	linear	vertical	vertical	1	1	yes	4	7	0,43
															bosd	same	orth	linear	vertical	vertical	1	2	no			
															bosd	same	orth	linear	vertical	vertical	2	3	no			
															bosd	same	orth	linear	vertical	vertical	2	4	no			
															bosd	same	orth	linear	vertical	vertical	2	5	no			
															bosd	same	orth	linear	left-right	horizontal	3	6	yes			
															hinge door/gate	same		linear	forward-back	horizontal	4	7	yes			
														2	bosd	opposite	orth	linear	vertical	vertical	1	1	yes	4	11	0,27
															bosd	opposite	orth	linear	vertical	vertical	1	2	no			
															bosd	opposite	orth	linear	vertical	vertical	1	3	no			
															bosd	opposite	orth	linear	vertical	vertical	1	4	no			
															bosd	opposite	orth	linear	vertical	vertical	2	5	no			
															bosd	opposite	orth	linear	vertical	vertical	2	6	no			
															bosd	opposite	orth	linear	vertical	vertical	2	7	no			
															bosd	opposite	orth	linear	vertical	vertical	3	8	no			
															bosd	opposite	orth	linear	vertical	vertical	3	9	no			
															bosd	opposite	orth	linear	left-right	horizontal	3	10	yes			
															hinge door/gate	opposite		linear	forward-back	horizontal	4	11	yes			
gen.Germ.	2086	1	1	1	0	1	0	1	0	0	0	0	0	1	bosd	same	orth	linear	vertical	vertical	1	1	yes	2	3	1
															bosd	same	orth	linear	left-right	horizontal	1	2	yes			
															hinge door/gate	same		linear	forward-back	horizontal	2	3	yes			
gen.Germ.	2087	1	1	1	0	1	0	1	0	0	0	0	0	1	bosd	same	orth	linear	vertical	vertical	1	1	no	3	4	0,75
															bosd	same	orth	linear	vertical	vertical	2	2	yes			
															bosd	same	orth	linear	left-right	horizontal	2	3	yes			
															hinge door/gate	same		linear	forward-back	horizontal	3	4	yes			
gen.Germ.	2088	1	1	1	0	1	0	1	0	0	0	0	0	1	bosd	opposite	orth	linear	vertical	vertical	1	1	no	3	7	0,43
															bosd	opposite	orth	linear	vertical	vertical	1	2	no			
															bosd	opposite	orth	linear	vertical	vertical	1	3	no			
															bosd	opposite	orth	linear	vertical	vertical	1	4	no			
															bosd	opposite	orth	linear	vertical	vertical	2	5	yes			
															bosd	opposite	orth	linear	left-right	horizontal	2	6	yes			
															hinge door/gate	opposite		linear	forward-back	horizontal	3	7	yes			
gen.Germ.	2090	1	1	1	0	1	0	1	0	0	0	0	0	1	bosd	same	orth	linear	vertical	vertical	1	1	no	3	4	0,75
															bosd	same	orth	linear	vertical	vertical	2	2	yes			
															bosd	same	orth	linear	left-right	horizontal	2	3	yes			
															hinge door/gate	same		linear	forward-back	horizontal	3	4	yes			
gen.Germ.	2091	1	1	1	0	1	0	0	1	0	0	0	0	1	boupdo	same	orth	linear	vertical	vertical	1	1	no	6	7	0,29
															boupdo	same	orth	linear	vertical	vertical	2	2	no			
															boupdo	same	orth	linear	vertical	vertical	3	3	no			
															boupdo	same	orth	linear	vertical	vertical	4	4	no			
															boupdo	same	orth	linear	vertical	vertical	5	5	no			
															boupdo	same	orth	linear	vertical	vertical	5	6	yes			
															hinge door/gate	same		linear	forward-back	horizontal	6	7	yes			
gen.Germ.	2093	1	1	1	0	1	0	0	0	0	0	1	0	1	handle	same	orth	linear	vertical	vertical	1	1	no	4	8	0,25
															handle	same	orth	linear	vertical	vertical	1	2	no			
															handle	same	orth	linear	vertical	vertical	1	3	no			
															handle	same	orth	linear	vertical	vertical	1	4	no			
															handle	same	orth	linear	vertical	vertical	2	5	no			
															handle	same	orth	linear	vertical	vertical	2	6	no			
															handle	same	orth	linear	vertical	vertical	3	7	yes			
															hinge door/gate	same		linear	forward-back	horizontal	4	8	yes			
gen.Germ.	2096	2	2	2	1	1	0	0	1	0	0	1	0	1	boupdo	orth	orth	twist	vertical	vertical	1	1	yes	2	2	1
															slide door	orth		linear	left-right	horizontal	1	1	yes			
														2	handle	opposite	orth	linear	vertical	vertical	1	1	yes	2	2	1
															hinge door/gate	opposite		linear	forward-back	horizontal	2	2	yes			
gen.Germ.	2103	1	1	1	0	1	0	1	0	0	0	0	0	1	bosd	same	orth	linear	vertical	vertical	1	1	no	3	6	0,5
															bosd	same	orth	linear	vertical	vertical	1	2	no			
															bosd	same	orth	linear	vertical	vertical	1	3	no			
															bosd	same	orth	linear	vertical	vertical	2	4	yes			
															bosd	same	orth	linear	left-right	horizontal	2	5	yes			
															hinge door/gate	same		linear	forward-back	horizontal	3	6	yes			
gen.Germ.	2104	1	1	1	0	1	0	0	1	0	0	0	0	1	boupdo	same	orth	linear	vertical	vertical	1	1	no	2	6	0,33
															boupdo	same	orth	linear	vertical	vertical	1	2	no			
															boupdo	same	orth	linear	vertical	vertical	1	3	no			
															boupdo	same	orth	linear	vertical	vertical	1	4	no			
															boupdo	same	orth	linear	vertical	vertical	1	5	yes			
															hinge door/gate	same		linear	forward-back	horizontal	2	6	yes			
gen.Germ.	2106	1	1	1	0	1	0	1	0	0	0	0	0	1	bosd	same	orth	linear	left-right	horizontal	1	1	no	2	3	0,67
															bosd	same	orth	linear	left-right	horizontal	1	2	yes			
															hinge door/gate	same		linear	forward-back	horizontal	2	3	yes			
gen.Germ.	2107	1	1	1	0	1	0	1	0	0	0	0	0	1	bosd	same	orth	linear	left-right	horizontal	1	1	no	4	11	0,27
															bosd	same	orth	linear	left-right	horizontal	1	2	no			
															bosd	same	orth	linear	left-right	horizontal	1	3	no			
															bosd	same	orth	linear	vertical	vertical	1	4	no			
															bosd	same	orth	linear	left-right	horizontal	2	5	no			
															bosd	same	orth	linear	left-right	horizontal	2	6	no			
															bosd	same	orth	linear	left-right	horizontal	3	7	no			
															bosd	same	orth	linear	left-right	horizontal	3	8	no			
															bosd	same	orth	linear	vertical	vertical	3	9	yes			
															bosd	same	orth	linear	left-right	horizontal	3	10	yes			
															hinge door/gate	same		linear	forward-back	horizontal	4	11	yes			
gen.Germ.	2109	1	1	1	0	1	0	1	0	0	0	0	0	1	bosd	opposite	orth	linear	left-right	horizontal	1	1	no	6	7	0,43
															bosd	opposite	orth	linear	left-right	horizontal	2	2	no			
															bosd	opposite	orth	linear	left-right	horizontal	3	3	no			
															bosd	opposite	orth	linear	vertical	vertical	4	4	yes			
															bosd	opposite	orth	linear	left-right	horizontal	4	5	yes			
															bosd	opposite	orth	linear	forward-back	horizontal	5	6	no			
															hinge door/gate	opposite		linear	forward-back	horizontal	6	7	yes			
gen.Germ.	2113	1	1	1	0	1	0	1	0	0	0	0	0	1	bosd	opposite	orth	linear	left-right	horizontal	1	1	no	3	5	0,6
															bosd	opposite	orth	linear	vertical	vertical	1	2	no			
															bosd	opposite	orth	linear	vertical	vertical	2	3	yes			
															bosd	opposite	orth	linear	left-right	horizontal	2	4	yes			
															hinge door/gate	opposite		linear	forward-back	horizontal	3	5	yes			
gen.Germ.	2121	1	1	2	0	0	1	1	0	0	0	0	0	1	bosd / bar at gate	orth	orth	linear	left-right	horizontal	1	1	no	5	5	0,4
															bosd	orth	orth	linear	left-right	horizontal	2	2	yes			
														2	bosd / bar at gate	orth	orth	linear	left-right	horizontal	1	1	no			
															bosd	orth	orth	linear	left-right	horizontal	2	2	no			
															bosd	orth	orth	linear	left-right	horizontal	3	3	yes			
gen.Germ.	2131	1	1	1	0	1	0	1	0	0	0	0	0	1	bosd	same	orth	linear	vertical	vertical	1	1	yes	3	4	0,75
															bosd	same	orth	linear	vertical	vertical	2	2	no			
															bosd	same	orth	linear	left-right	horizontal	2	3	yes			
															hinge door/gate	same		linear	forward-back	horizontal	3	4	yes			
gen.Germ.	2133	1	1	1	1	0	0	0	1	0	0	0	0	1	boupdo	orth	orth	twist	vertical	vertical	1	1	no	6	6	0,33
															boupdo	orth	orth	twist	vertical	vertical	2	2	no			
															boupdo	orth	orth	twist	vertical	vertical	3	3	no			
															boupdo	orth	orth	twist	vertical	vertical	4	4	yes			
															slide door	orth		linear	left-right	horizontal	1	1	no			
															slide door	orth		linear	left-right	horizontal	2	2	yes			
gen.Germ.	2136	1	1	1	0	1	0	0	0	0	0	1	0	1	handle	opposite	orth	linear	vertical	vertical	1	1	yes	2	2	1
															hinge door/gate	opposite		linear	forward-back	horizontal	2	2	yes			
gen.Germ.	2137	1	1	1	0	1	0	0	0	0	0	1	0	1	handle	opposite	orth	linear	vertical	vertical	1	1	yes	2	2	1
															hinge door/gate	opposite		linear	forward-back	horizontal	2	2	yes			
door_gate_Germ.	2179	1	1	2	0	1	0	1	0	0	0	1	0	1	bosd	same	orth	linear	vertical	vertical	1	1	yes	4	4	1
															hinge door/gate	same		linear	left-right	horizontal	2	2	yes			
														2	bosd	opposite	orth	linear	vertical	vertical	1	1	yes			
															hinge door/gate	opposite		linear	left-right	horizontal	2	2	yes			
door_gate_Germ.	2178	1	1	1	0	1	0	1	0	0	0	0	0	1	bosd	opposite	orth	linear	vertical	vertical	1	1	no	4	5	0,6
															bosd	opposite	orth	linear	vertical	vertical	2	2	yes			
															bosd	opposite	orth	linear	left-right	horizontal	2	3	yes			
															hinge door/gate	opposite		linear	forward-back	horizontal	3	4	no			
															hinge door/gate	opposite		linear	forward-back	horizontal	4	5	yes			
door_gate_Germ.	2176	1	1	2	0	1	0	1	0	0	0	0	0	1	bosd	opposite	orth	linear	vertical	vertical	1	1	no	6	8	0,75
															bosd	opposite	orth	linear	vertical	vertical	2	2	no			
															bosd	opposite	orth	linear	vertical	vertical	3	3	yes			
															bosd	opposite	orth	linear	left-right	horizontal	3	4	yes			
															hinge door/gate	opposite		linear	forward-back	horizontal	4	5	yes			
														2	bosd	opposite	orth	linear	vertical	vertical	1	6	yes			
															bosd	opposite	orth	linear	left-right	horizontal	1	7	yes			
															hinge door/gate	opposite		linear	forward-back	horizontal	2	8	yes			
door_gate_Germ.	2177	1	1	1	0	1	0	1	0	0	0	0	0	1	bosd	same	orth	linear	vertical	vertical	1	1	no	4	6	0,5
															bosd	same	orth	linear	vertical	vertical	2	2	no			
															bosd	same	orth	linear	vertical	vertical	3	3	yes			
															bosd	same	orth	linear	left-right	horizontal	3	4	yes			
															bosd	same	orth	linear	left-right	horizontal	3	5	no			
															hinge door/gate	same		linear	forward-back	horizontal	4	6	yes			
door_gate_Germ.	2204	1	1	1	0	1	0	1	0	0	0	0	0	1	bosd	same	orth	linear	vertical	vertical	1	1	no	2	4	0,75
															bosd	same	orth	linear	vertical	vertical	1	2	yes			
															bosd	same	orth	linear	left-right	horizontal	1	3	yes			
															hinge door/gate	same		linear	forward-back	horizontal	2	4	yes			
door_gate_Germ.	2205	1	1	2	0	0	1	0	0	0	0	2	0	1	ehandle	orth	orth	linear	vertical	vertical	1	1	yes	2	5	0,8
															ehandle	orth	orth	linear	left-right	horizontal	1	2	yes			
														2	ehandle	orth	orth	linear	vertical	vertical	2	3	no			
															ehandle	orth	orth	linear	vertical	vertical	2	4	yes			
															ehandle	orth	orth	linear	left-right	horizontal	2	5	yes			
door_gate_Germ.	2253	1	1	1	1	0	0	1	0	0	0	0	0	1	boupdo	orth	orth	linear	vertical	vertical	1	1	yes	2	3	0,67
															slide door	orth		linear	left-right	horizontal	1	2	yes			
															slide door	orth		linear	left-right	horizontal	2	3	no			
door_gate_Germ.	2341	1	1	1	0	1	0	1	0	0	0	0	0	1	bosd	same	orth	linear	forward-back	horizontal	1	1	no	2	4	0,5
															bosd	same	orth	linear	forward-back	horizontal	1	2	no			
															bosd	same	orth	linear	forward-back	horizontal	1	3	yes			
															hinge door/gate	same		linear	forward-back	horizontal	2	4	yes			
door_gate_Engl.	2207	1	1	1	0	1	0	1	0	0	0	0	0	1	bosd	same	orth	linear	forward-back	horizontal	1	1	no	2	6	0,33
															bosd	same	orth	linear	forward-back	horizontal	1	2	no			
															bosd	same	orth	linear	forward-back	horizontal	1	3	no			
															bosd	same	orth	linear	forward-back	horizontal	1	4	no			
															bosd	same	orth	linear	forward-back	horizontal	1	5	yes			
															hinge door/gate	same		linear	forward-back	horizontal	2	6	yes			
door_gate_Engl.	2208	1	1	1	0	1	0	1	0	0	0	0	0	1	bosd	same	orth	linear	vertical	vertical	1	1	yes	2	3	1
															bosd	same	orth	linear	left-right	horizontal	1	2	yes			
															hinge door/gate	same		linear	forward-back	horizontal	2	3	yes			
door_gate_Engl.	2209	1	1	1	0	1	0	0	1	0	0	0	0	1	bosd	same	orth	twist	vertical	vertical	1	1	yes	2	3	1
															bosd	same	orth	twist	left-right	horizontal	1	2	yes			
															hinge door/gate	same		linear	forward-back	horizontal	2	3	yes			
door_gate_Engl.	2277	1	1	1	0	1	0	0	1	0	0	0	0	1	bosd	same	orth	linear	vertical	vertical	1	1	yes	4	5	0,6
															bosd	same	orth	linear	left-right	horizontal	1	2	yes			
															hinge door/gate	same		linear	forward-back	horizontal	2	3	yes			
															hinge door/gate	same		linear	forward-back	horizontal	3	4	no			
															hinge door/gate	same		linear	forward-back	horizontal	4	5	no			
door_gate_Engl.	2414	1	1	1	0	0	1	0	1	0	0	0	0	1	boards of front door	orth	orth	linear	vertical	vertical	1	1	no	8	9	0,88
															boards of front door	orth	orth	linear	vertical	vertical	1	2	yes			
															boards of front door	orth	orth	linear	vertical	vertical	2	3	yes			
															boards of front door	orth	orth	linear	vertical	vertical	3	4	yes			
															boards of front door	orth	orth	linear	vertical	vertical	4	5	yes			
															boards of front door	orth	orth	linear	vertical	vertical	5	6	yes			
															boards of front door	orth	orth	linear	vertical	vertical	6	7	yes			
															boards of front door	orth	orth	linear	vertical	vertical	7	8	yes			
															boards of front door	orth	orth	linear	vertical	vertical	8	9	yes			
door_gate_Engl.	2429	1	1	1	0	1	0	1	1	0	0	0	0	1	bosd	same	orth	twist	forward-back	horizontal	1	1	no	2	10	0,2
															bosd	same	orth	twist	forward-back	horizontal	1	2	no			
															bosd	same	orth	twist	forward-back	horizontal	1	3	no			
															bosd	same	orth	twist	forward-back	horizontal	1	4	no			
															bosd	same	orth	twist	forward-back	horizontal	1	5	no			
															bosd	same	orth	twist	forward-back	horizontal	1	6	no			
															bosd	same	orth	twist	forward-back	horizontal	1	7	no			
															bosd	same	orth	twist	forward-back	horizontal	1	8	yes			
															bosd	same	orth	linear	left-right	horizontal	1	9	no			
															bosd	same	orth	linear	left-right	horizontal	2	10	yes			
															hinge door/gate	same		linear	forward-back	horizontal	3	11	yes	2	7	0,29
door_gate_Engl.	2478	1	1	1	0	1	0	1	0	0	0	0	0	1	bosd	same	orth	twist	vertical	vertical	1	1	no			
															bosd	same	orth	twist	vertical	vertical	1	2	no			
															bosd	same	orth	twist	vertical	vertical	1	3	no			
															bosd	same	orth	twist	vertical	vertical	1	4	no			
															bosd	same	orth	twist	vertical	vertical	1	5	yes			
															bosd	same	orth	twist	left-right	horizontal	1	6	yes			
															hinge door/gate	same	orth	linear	forward-back	horizontal	2	7	yes			
door_gate_Germ.	2512	1	1	1	1	0	0	0	1	0	0	0	0	1	boupdo	orth	orth	linear	vertical	vertical	1	1	no	2	3	0,67
															boupdo	orth	orth	linear	vertical	vertical	2	2	yes			
															slide door	orth		linear	left-right	horizontal	2	3	yes			
door_gate_Germ.	2513	1	1	1	0	1	0	1	0	0	0	0	0	1	bosd	same	orth	linear	forward-back	horizontal	1	1	yes	2	2	1
															hinge door/gate	same		linear	forward-back	horizontal	2	2	yes			
door_gate_Germ.	2514	1	1	2	0	1	0	1	0	0	0	0	0	1	bosd	same	orth	linear	vertical	vertical	1	1	yes	2	3	1
															bosd	same	orth	linear	left-right	horizontal	1	2	yes			
															hinge door/gate	same		linear	forward-back	horizontal	2	3	yes			
														2	bosd	opposite	orth	linear	vertical	vertical	1	1	yes	3	4	0,75
															bosd	opposite	orth	linear	vertical	vertical	2	2	no			
															bosd	opposite	orth	linear	left-right	horizontal	2	3	yes			
															hinge door/gate	opposite		linear	forward-back	horizontal	3	4	yes			
door_gate_Germ.	2515	1	1	1	0	1	0	1	0	0	0	0	0	1	bosd	same	orth	linear	left-right	horizontal	1	1	no	2	3	0,67
															bosd	same	orth	linear	left-right	horizontal	1	2	yes			
															hinge door/gate	same		linear	forward-back	horizontal	2	3	yes			
door_gate_Germ.	2516	1	1	1	0	1	0	1	0	0	0	0	0	1	boupdo	same	orth	linear	vertical	vertical	1	1	no	6	9	0,33
															boupdo	same	orth	linear	vertical	vertical	1	2	no			
															boupdo	same	orth	linear	vertical	vertical	2	3	no			
															boupdo	same	orth	linear	vertical	vertical	3	4	no			
															boupdo	same	orth	linear	vertical	vertical	3	5	no			
															boupdo	same	orth	linear	vertical	vertical	4	6	no			
															boupdo	same	orth	linear	vertical	vertical	5	7	yes			
															boupdo	same	orth	linear	left-right	horizontal	5	8	yes			
															hinge door/gate	same		linear	forward-back	horizontal	6	9	yes			
door_gate_Germ.	2517	1	1	1	0	1	0	0	1	0	0	0	0	1	bosd	same	orth	twist	left-right	horizontal	1	1	no	3	11	0,18
															bosd	same	orth	twist	left-right	horizontal	1	2	no			
															bosd	same	orth	twist	left-right	horizontal	1	3	no			
															bosd	same	orth	twist	left-right	horizontal	1	4	no			
															bosd	same	orth	twist	left-right	horizontal	2	5	no			
															bosd	same	orth	twist	left-right	horizontal	2	6	no			
															bosd	same	orth	twist	left-right	horizontal	2	7	no			
															bosd	same	orth	twist	left-right	horizontal	2	8	no			
															bosd	same	orth	twist	left-right	horizontal	2	9	no			
															bosd	same	orth	twist	left-right	horizontal	2	10	yes			
															hinge door/gate	same		linear	forward-back	horizontal	3	11	yes			
door_gate_Germ.	2518	1	1	2	0	1	0	1	0	0	0	0	0	1	bosd	opposite	orth	linear	left-right	horizontal	1	1	yes	7	9	0,22
															bosd	opposite	orth	linear	left-right	horizontal	2	2	no			
															bosd	opposite	orth	linear	left-right	horizontal	3	3	no			
															bosd	opposite	orth	linear	left-right	horizontal	4	4	no			
															hinge door/gate	opposite		linear	forward-back	horizontal	5	5	yes			
															hinge door/gate	opposite		linear	forward-back	horizontal	5	6	no			
															hinge door/gate	opposite		linear	forward-back	horizontal	5	7	no			
															hinge door/gate	opposite		linear	forward-back	horizontal	6	8	no			
															hinge door/gate	opposite		linear	forward-back	horizontal	7	9	no			
														2	bosd	opposite	orth	linear	left-right	horizontal	1	1	yes	5	8	0,25
															bosd	opposite	orth	linear	left-right	horizontal	1	2	no			
															bosd	opposite	orth	linear	left-right	horizontal	1	3	no			
															bosd	opposite	orth	linear	left-right	horizontal	2	4	no			
															bosd	opposite	orth	linear	left-right	horizontal	2	5	no			
															hinge door/gate	opposite		linear	forward-back	horizontal	3	6	yes			
															hinge door/gate	opposite		linear	forward-back	horizontal	4	7	no			
															hinge door/gate	opposite		linear	forward-back	horizontal	5	8	no			
door_gate_Germ.	2538	1	1	1	0	1	0	1	0	0	0	0	0	1	bosd	same	orth	linear	left-right	horizontal	1	1	yes	3	6	0,33
															bosd	same	orth	linear	left-right	horizontal	2	2	no			
															bosd	same	orth	linear	left-right	horizontal	2	3	no			
															bosd	same	orth	linear	left-right	horizontal	2	4	no			
															bosd	same	orth	linear	left-right	horizontal	2	5	no			
															hinge door/gate	same		linear	forward-back	horizontal	3	6	yes			
door_gate_Germ.	2539	1	1	1	1	0	0	0	1	0	0	0	0	1	boupdo	orth	orth	linear	vertical	vertical	1	1	yes	5	5	0,4
															boupdo	orth	orth	linear	vertical	vertical	2	2	no			
															boupdo	orth	orth	linear	vertical	vertical	3	3	no			
															slide door	orth		linear	left-right	horizontal	4	4	yes			
															slide door	orth		linear	left-right	horizontal	5	5	no			
door_gate_Germ.	2519	1	1	1	1	0	0	1	0	0	0	0	0	1	bosd	orth	same	twist	vertical	vertical	1	1	yes	7	10	0,3
															bosd	orth	same	twist	vertical	vertical	2	2	no			
															bosd	orth	same	twist	vertical	vertical	3	3	no			
															bosd	orth	same	twist	vertical	vertical	3	4	no			
															bosd	orth	same	twist	vertical	vertical	4	5	no			
															bosd	orth	same	twist	vertical	vertical	5	6	no			
															bosd	orth	same	twist	vertical	vertical	6	7	no			
															bosd	orth	same	twist	vertical	vertical	6	8	no			
															slide door	orth		linear	forward-back	horizontal	6	9	yes			
															slide door	orth		linear	forward-back	horizontal	7	10	yes			
door_gate_Germ.	2520	1	1	2	0	1	0	0	0	0	0	0	1	1	hinge door/gate	same		linear	forward-back	horizontal	1	1	yes	6	13	0,38
														2	carabiner	same	orth	linear	vertical	vertical	2	2	yes			
															carabiner	same	orth	twist	twist	twist	3	3	yes			
															carabiner	same	orth	twist	twist	twist	4	4	no			
															carabiner	same	orth	twist	twist	twist	4	5	no			
															carabiner	same	orth	twist	twist	twist	4	6	no			
															carabiner	same	orth	twist	twist	twist	4	7	no			
															carabiner	same	orth	twist	twist	twist	4	8	no			
															carabiner	same	orth	twist	twist	twist	4	9	no			
															carabiner	same	orth	linear	vertical	vertical	5	10	yes			
															carabiner	same	orth	linear	vertical	vertical	5	11	no			
															slide door	orth		linear	left-right	horizontal	6	12	yes			
															slide door	orth		linear	left-right	horizontal	6	13	no			
door_gate_Germ.	2521	1	1	1	0	1	0	1	0	0	0	0	0	1	bosd	same	orth	linear	vertical	vertical	1	1	yes	2	3	1
															bosd	same	orth	linear	left-right	horizontal	1	2	yes			
															hinge door/gate	same		linear	forward-back	horizontal	2	3	yes			
door_gate_Germ.	2522	1	1	1	0	1	0	1	0	0	0	0	0	1	bosd	opposite	orth	linear	vertical	vertical	1	1	no	3	5	0,4
															bosd	opposite	orth	linear	vertical	vertical	1	2	no			
															bosd	opposite	orth	linear	left-right	horizontal	2	3	yes			
															bosd	opposite	orth	linear	left-right	horizontal	2	4	no			
															hinge door/gate	opposite		linear	forward-back	horizontal	3	5	yes			
door_gate_Germ.	2523	1	1	1	0	1	0	0	0	0	0	0	1	1	carabiner	opposite	orth	twist	twist	twist	1	1	yes	3	11	0,18
															carabiner	opposite	orth	twist	twist	twist	1	2	no			
															carabiner	opposite	orth	twist	twist	twist	2	3	no			
															carabiner	opposite	orth	twist	twist	twist	2	4	no			
															carabiner	opposite	orth	twist	twist	twist	2	5	no			
															carabiner	opposite	orth	twist	twist	twist	2	6	no			
															carabiner	opposite	orth	twist	twist	twist	2	7	no			
															carabiner	opposite	orth	twist	twist	twist	2	8	no			
															carabiner	opposite	orth	twist	twist	twist	2	9	no			
															carabiner	opposite	orth	twist	twist	twist	2	10	no			
															hinge door/gate	opposite									
door_gate_Germ.	2524	1	1	1	0	0	1	0	0	0	0	0	1	1	carabiner on chain /	orth	orth	twist	twist	twist	1	1	yes	1	4	0,25
															carabiner	orth	orth	twist	twist	twist	1	2	no			
															carabiner	orth	orth	twist	twist	twist	1	3	no			
															carabiner	orth	orth	twist	twist	twist	1	4	no			
door_gate_Germ.	2525	1	1	1	0	1	0	1	0	0	0	0	0	1	bosd	same	orth	linear	left-right	horizontal	1	1	yes	2	3	0,66
															bosd	same	orth	linear	left-right	horizontal	1	2	no			
															hinge door/gate	same		linear	forward-back	horizontal	2	3	yes			
door_gate_Germ.	2526	1	1	1	0	1	0	0	1	0	0	0	0	1	boupdo	same	orth	linear	vertical	vertical	1	1	yes	2	5	0,4
															boupdo	same	orth	linear	vertical	vertical	1	2	no			
															boupdo	same	orth	linear	vertical	vertical	1	3	no			
															boupdo	same	orth	linear	vertical	vertical	1	4	no			
															hinge door/gate	same							yes			
door_gate_Germ.	2527	1	1	1	0	1	0	1	0	0	0	0	0	1	bosd	same	orth	linear	vertical	vertical	1	1	yes	3	5	0,6
															bosd	same	orth	linear	vertical	vertical	2	2	no			
															bosd	same	orth	linear	vertical	vertical	2	3	no			
															bosd	same	orth	linear	left-right	horizontal	2	4	yes			
															hinge door/gate	same		linear	forward-back	horizontal	3	5	yes			
door_gate_Germ.	2528	1	1	1	0	1	0	0	0	0	0	1	0	1	handle	same	orth	linear	vertical	vertical	1	1	yes	2	3	0,67
															handle	same	orth	linear	vertical	vertical	1	2	no			
															hinge door/gate	same		linear	forward-back	horizontal	2	3	yes			
door_gate_Germ.	2529	1	1	1	0	1	0	0	0	0	0	1	0	1	handle	same	orth	linear	vertical	vertical	1	1	yes	2	3	0,67
															handle	same	orth	linear	vertical	vertical	1	2	no			
															hinge door/gate	same		linear						
door_gate_Germ.	2530	1	1	1	0	1	0	0	0	0	1	0	0	1	secch	same	orth	linear	vertical	vertical	1	1	yes	3	8	0,37
															secch	same	orth	linear	vertical	vertical	1	2	no			
															secch	same	orth	twist	twist	twist	2	4	no			
															secch	same	orth	twist	twist	twist	2	5	no			
															secch	same	orth	twist	twist	twist	2	6	no			
															secch	same	orth	twist	twist	twist	2	7	no			
															hinge door/gate	same							yes			
door_gate_Germ.	2531	1	1	1	0	1	0	1	0	0	0	0	0	1	bosd	same	orth	linear	left-right	horizontal	1	1	yes	2	2	1
															hinge door/gate	same		linear						
door_gate_Germ.	2532	1	1	1	0	1	0	0	1	0	0	0	0	1	boupdo	same	orth	linear	vertical	vertical	1	2	yes	3	8	0,25
															boupdo	same	orth	linear	vertical	vertical	1	3	no			
															boupdo	same	orth	linear	vertical	vertical	2	4	no			
															boupdo	same	orth	linear	vertical	vertical	2	5	no			
															boupdo	same	orth	linear	vertical	vertical	2	6	no			
															boupdo	same	orth	linear	vertical	vertical	2	7	no			
															hinge door/gate	same		linear	forward-back	horizontal	3	8	yes			
door_gate_Germ.	2533	1	1	1	0	1	0	0	0	0	0	1	0	1	handle	same	orth	linear	vertical	vertical	1	1	yes	2	3	0,67
															handle	same	orth	linear	vertical	vertical	1	2	no			
															hinge door/gate	same		linear	forward-back	horizontal	2	3	yes			
door_gate_Germ.	2534	1	1	1	0	1	0	0	1	0	0	0	0	1	boupdo	same	orth	linear	vertical	vertical	1	1	yes	3	7	0,29
															boupdo	same	orth	linear	vertical	vertical	1	2	no			
															boupdo	same	orth	linear	vertical	vertical	1	3	no			
															boupdo	same	orth	linear	vertical	vertical	2	4	no			
															boupdo	same	orth	linear	vertical	vertical	2	5	no			
															boupdo	same	orth	linear	vertical	vertical	2	6	no			
															hinge door/gate	same		linear	forward-back	horizontal	3	7	yes			
door_gate_Germ.	2535	1	1	1	0	1	0	0	0	0	0	1	0	1	handle	same	orth	linear	vertical	vertical	1	1	yes	2	4	0,5
															handle	same	orth	linear	vertical	vertical	1	2	no			
															handle	same	orth	linear	vertical	vertical	1	3	no			
															hinge door/gate	same		linear	forward-back	horizontal	2	4	yes			
door_gate_Germ.	2536	1	1	1	0	1	0	1	0	0	0	0	0	1	bosd	same	orth	linear	vertical	vertical	1	1	yes	3	6	0,5
															bosd	same	orth	linear	vertical	vertical	1	2	no			
															bosd	same	orth	linear	vertical	vertical	1	3	no			
															bosd	same	orth	linear	vertical	vertical	1	4	no			
															bosd	same	orth	linear	left-right	horizontal	2	5	yes			
															hinge door/gate	same		linear	forward-back	horizontal	3	6	yes			
door_gate_Germ.	2537	1	1	1	0	1	0	0	0	0	0	1	0	1	handle	same	orth	linear	vertical	vertical	1	1	yes	1	2	1
															hinge door/gate	same		linear	forward-back	horizontal	2	2	yes			
door_gate_Germ.	2540	1	3	1	0	1	0	1	1	1	0	0	0	1	key	same	orth	linear	twist	twist	1	1	yes	5	12	0,42
															key	same	orth	linear	twist	twist	1	2	no			
															key	same	orth	linear	twist	twist	2	3	no			
															key	same	orth	linear	twist	twist	2	4	no			
															key	same	orth	linear	twist	twist	2	5	no			
															key	same	orth	linear	twist	twist	2	6	no			
															key	same	orth	linear	twist	twist	2	7	no			
															key	same	orth	linear	twist	twist	2	8	no			
														2	padlock/ boupdo	same	orth	linear	vertical	vertical	3	9	yes			
														3	bosd	same	orth	linear	vertical	vertical	4	10	yes			
															bosd	same	orth	linear	left-right	horizontal	4	11	yes			
														4	hinge door/gate	same		linear	forward-back	horizontal	5	12	yes			
